# Pan-cancer analysis of homozygous deletions in primary tumours uncovers rare tumour suppressors

**DOI:** 10.1038/s41467-017-01355-0

**Published:** 2017-10-31

**Authors:** Jiqiu Cheng, Jonas Demeulemeester, David C. Wedge, Hans Kristian M. Vollan, Jason J. Pitt, Hege G. Russnes, Bina P. Pandey, Gro Nilsen, Silje Nord, Graham R. Bignell, Kevin P. White, Anne-Lise Børresen-Dale, Peter J. Campbell, Vessela N. Kristensen, Michael R. Stratton, Ole Christian Lingjærde, Yves Moreau, Peter Van Loo

**Affiliations:** 1 Department of Electrical Engineering (ESAT) and iMinds Future Health Department, University of Leuven, Kasteelpark Arenberg 10, B-3001 Leuven, Belgium; 2 Department of Genetics, Institute for Cancer Research, Oslo University Hospital Radiumhospitalet, N-0310 Oslo, Norway; 3 The Francis Crick Institute, 1 Midland Road, London, NW1 1AT UK; 4 Department of Human Genetics, University of Leuven, Herestraat 49, B-3000 Leuven, Belgium; 5 Wellcome Trust Sanger Institute, Hinxton, Cambridge, CB10 1SA UK; 6 Big Data Institute, University of Oxford, Old Road, Oxford, OX3 7LF UK; 7 Institute for Genomics and Systems Biology, University of Chicago, 900 East 57th Street, Chicago, IL 60637 USA; 8 Committee on Genetics, Genomics, and Systems Biology, University of Chicago, 920 East 58th Street, Chicago, IL 60637 USA; 9 Department of Pathology, Oslo University Hospital Radiumhospitalet, N-0310 Oslo, Norway; 10 Department of Informatics and Centre for Cancer Biomedicine, University of Oslo, N-0424 Oslo, Norway; 11 Department of Ecology and Evolution, University of Chicago, 1101 East 57th Street, Chicago, IL 60637 USA; 12 Department of Human Genetics, University of Chicago, 920 East 58th Street, Chicago, IL 60637 USA; 13 Tempus Labs, Inc., Chicago, IL USA

## Abstract

Homozygous deletions are rare in cancers and often target tumour suppressor genes. Here, we build a compendium of 2218 primary tumours across 12 human cancer types and systematically screen for homozygous deletions, aiming to identify rare tumour suppressors. Our analysis defines 96 genomic regions recurrently targeted by homozygous deletions. These recurrent homozygous deletions occur either over tumour suppressors or over fragile sites, regions of increased genomic instability. We construct a statistical model that separates fragile sites from regions showing signatures of positive selection for homozygous deletions and identify candidate tumour suppressors within those regions. We find 16 established tumour suppressors and propose 27 candidate tumour suppressors. Several of these genes (including *MGMT*, *RAD17*, and *USP44*) show prior evidence of a tumour suppressive function. Other candidate tumour suppressors, such as *MAFTRR*, *KIAA1551*, and *IGF2BP2*, are novel. Our study demonstrates how rare tumour suppressors can be identified through copy number meta-analysis.

## Abstract (web summary)

Homozygous deletions are rare in cancers and often target tumour suppressor genes. Here, the authors conduct pan-cancer analyses and apply statistical modelling to identify 27 candidate tumour suppressors, including *MAFTRR*, *KIAA1551*, and *IGF2BP2*.

## Introduction

The genomes of cancer cells are shaped by somatic mutations, including base substitutions, small insertions and deletions, genomic rearrangements, and copy number changes^1^. Many of these somatic changes are neutral passenger events, yet some confer a clonal selective advantage on cancer cells and drive oncogenesis^1^. The genes involved can be oncogenes, which are frequently hit by activating point mutations or amplifications and behave in a dominant fashion, or tumour suppressor genes, which are recurrently targeted by inactivating mutations such as truncating point mutations or deletions and display a recessive pattern^2–4^.

The most frequently used approach to identify cancer genes is assessing recurrence of non-synonymous somatic point mutations above background level. This is an extensive field that has been highly successful in the past. The power to detect cancer genes varies based on sample size and background mutation frequency: for most tumour types, current sample sizes are inadequate to reliably detect rare cancer genes mutated at ≤ 5% above background^5^.

Homozygous deletions require two independent hits and, in addition, any homozygous deletion covering a gene that carries out an essential function or confers a survival advantage is swiftly eliminated by negative selection. Therefore, homozygous deletions are rare and often focal in cancers. We reasoned that a systematic screen for homozygous deletions in a large series of cancer samples would be a powerful orthogonal way to specifically identify tumour suppressors, hypothesising that some tumour suppressors may be more prone to inactivation by homozygous deletions than by truncating point mutations. As traditional recurrence analysis has identified most or all frequently mutated tumour suppressors, our novel candidates are likely rare cancer genes, inactivated only in specific contexts.

Tumour samples contain both tumour cells and admixed normal cells in unknown proportions, complicating the distinction between homozygous and hemizygous deletions and hampering the discovery of tumour suppressors. Cancer cell lines represent a simplified model system that does not show this normal cell admixture and a comprehensive catalogue of homozygous deletions in cancer cell lines has been constructed^6^. However, this model system has important limitations: cancer cell lines are biased towards late stage cancers and metastases, they have accumulated mutations to adapt to growth in culture, their numbers are limited, and some cancer types do not lend themselves to growth in culture^7^.

The advent of SNP array technologies brought the realisation that it is theoretically possible to infer the fraction of admixed normal cells in primary tumours from array data^8^. This prompted the development of several computational methods and mathematical models, leading to mature approaches that can infer tumour purity and ploidy and separate copy number profiles from tumour cells and admixed normal cells^9–12^, opening novel avenues for studying tumorigenesis and tumour evolution^13^. These methods are able to reliably separate homozygous from hemizygous deletions, and have been able to pinpoint driver homozygous deletions in known and newly identified tumour suppressors^4^.

Here, we perform a systematic screen for homozygous deletions over a compendium of 2218 SNP arrays across 12 cancer types, aiming to identify rare tumour suppressors. We find 96 genomic regions recurrently targeted by homozygous deletions, overlapping 16 established tumour suppressors. However, homozygous deletions also occur frequently over fragile sites, chromosomal regions of increased genomic instability. We therefore construct a novel statistical model to separate fragile sites from regions containing tumour suppressors. This analysis extends the landscape of cancer genes by identifying 27 candidate tumour suppressors, adding to the emerging evidence for several tumour suppressor genes recently proposed in the literature and highlighting several novel candidates.

## Results

### Allele-specific copy number analysis across 12 tumour types

We constructed a compendium of 2218 publically available primary tumour samples hybridised to Affymetrix 250K StyI SNP arrays, encompassing cancers arising in 12 broadly defined tissue types (Supplementary Table 1, Supplementary Data 1). We employed ASCAT^10^ to infer tumour purity and ploidy and derive copy number profiles. ASCAT failed on 81 samples (3.7%) due to excessive noise, and these samples were excluded from further analyses. Of the 2137 cases that passed ASCAT analysis, 273 (12.8%) showed only few or no copy number aberrations, resulting in high variance tumour purity estimates. These samples were therefore excluded from the analysis of tumour purity and ploidy. As expected, the proportion of non-aberrant samples varied considerably between tumour types, with leukaemia showing the highest proportion of non-aberrant cases (55.6%), followed by lymphoma (22.5%) and sarcoma (20.1%) (Supplementary Fig. 1).

**Fig. 1:**
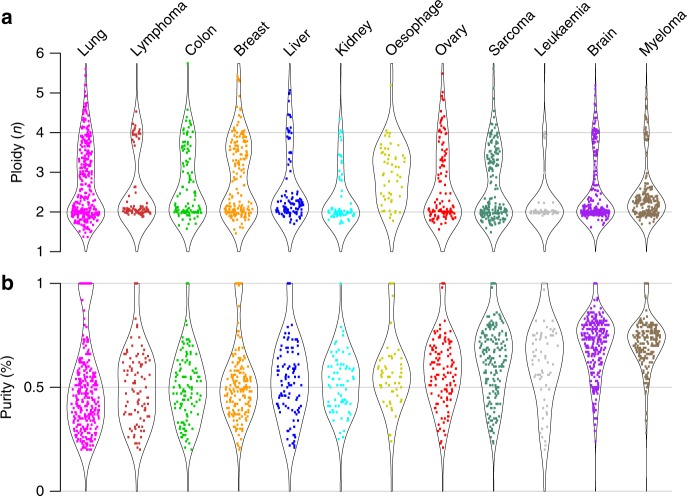
Tumour ploidy and purity across cancer types. A total of 2218 cancer samples hybridised to Affymetrix 250K StyI arrays, encompassing cancers arising in 12 broadly defined tissue types, were subjected to ASCAT analysis. ASCAT estimates of **a** tumour ploidy and **b** tumour purity are shown. Samples that failed ASCAT analysis (81; 3.6%) and samples that showed little to no copy number aberrations (273; 12.8%) and therefore have less accurate purity estimates are not included in these plots

Most cancer types displayed extensive variation in purity and ploidy (Fig. 1). All tumour types showed a substantial fraction of diploid or near-diploid cases, though with a much tighter distribution around the diploid state in some cancer types (notably leukaemias and lymphomas) than in others. Aneuploid cases are present in varying degree and with varying distribution across the 12 cancer types, with leukaemias displaying the lowest and oesophageal carcinomas the highest proportion of aneuploid cases (Fig. 1a). Normal cell admixture differed extensively both within and between cancer types (Fig. 1b). The CD138^+^ tumour cell enriched multiple myelomas showed high purity values. Among the remaining cancer types, the median purity was the lowest in lung cancer (46%) and the highest in brain cancers (70%). These findings are broadly consistent with previous ploidy measurements from chromosome counts^14^, as well as with a previous SNP array study on an overlapping series of samples^12^.

### Landscape of homozygous deletions in primary tumours

We performed a systematic screen for homozygous deletions across our 2137 primary tumours that passed ASCAT analysis. We identified 1865 homozygous deletions in total (median length: 315 kb), involving 826 tumours, each having on average 2.0 Mb (median 524 kb) homozygously deleted (Table 1, Supplementary Data 2). The 10% largest homozygous deletions together encompassed 51% of the total homozygously deleted sequence. In the remaining 1311 tumours, no homozygous deletions were identified. Cancer types showed marked differences in the number of homozygous deletions and the amount of sequence homozygously lost. Homozygous deletions were rarely found in renal cancer and more commonly in oesophageal cancer, lung cancer, and sarcoma (Table 1).

**Table 1:** Distribution of homozygous deletions across cancer types

Cancer type	Number of cases	Number of HDs	Homozygously deleted sequence (Mb)	Number of HDs per case (range)	Homozygously deleted sequence per case (kb)	Median length of HDs (kb)
Breast	193	138	95.28	0.72 (0–8)	494	165
Ovary	136	113	82.79	0.83 (0–6)	609	207
Colon	125	98	48.58	0.78 (0–9)	389	205
Liver	106	80	46.66	0.75 (0–11)	440	116
Kidney	73	25	6.89	0.34 (0–4)	94	97
Lung	402	535	536.89	1.33 (0–49)	1336	404
Brain	309	138	145.43	0.45 (0–6)	471	360
Oesophageal	58	66	74.52	1.14 (0–6)	1285	285
Sarcoma	244	353	425.48	1.45 (0–15)	1744	455
Myeloma	220	94	70.54	0.43 (0–14)	321	351
Leukaemia	151	99	57.76	0.66 (0–10)	382	231
Lymphoma	120	126	95.24	1.05 (0–12)	794	251
All	2137	1865	1686.06	0.87 (0–49)	789	315

*HD*, homozygous deletion

Diploid tumours tended to carry a higher number of homozygous deletions than tetraploid ones (ploidy > 2.7), while the size distribution of the deletions is the same for both (Supplementary Fig. 2a–d). Comparing homozygous deletion rates at a set of known tumour suppressors (see further) in diploid and tetraploid tumours, no differences were detected, except at the *RB1* locus, which was ~7× more frequently lost in tetraploid tumours (*p* = 1.01 × 10^−3^; Fisher–Boschloo’s exact unconditional test; Supplementary Fig. 2e). A positive correlation between Rb inactivation and polyploidy has previously been observed and its role in the cell cycle G_1_/S checkpoint further supports a role in tetraploidisation during tumorigenesis^15^.

**Fig. 2:**
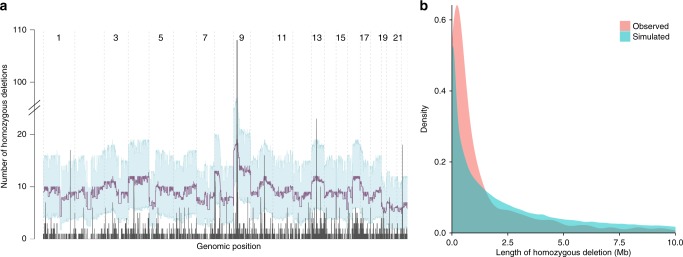
Homozygous deletions are non-randomly distributed across the genome. **a** Genomic distribution of the frequency of homozygous deletions (dark grey). Permutation test results, modelling homozygous deletions as a combination of two independent loss-of-heterozygosity events, are overlaid (mean and 95% confidence intervals in purple and light blue, respectively), indicating that homozygous deletions are strongly depleted across the genome due to negative selection. **b** Size distribution of homozygous deletions as observed and as predicted by the model above, indicating stronger negative selection against large homozygous deletions

**Fig. 3:**
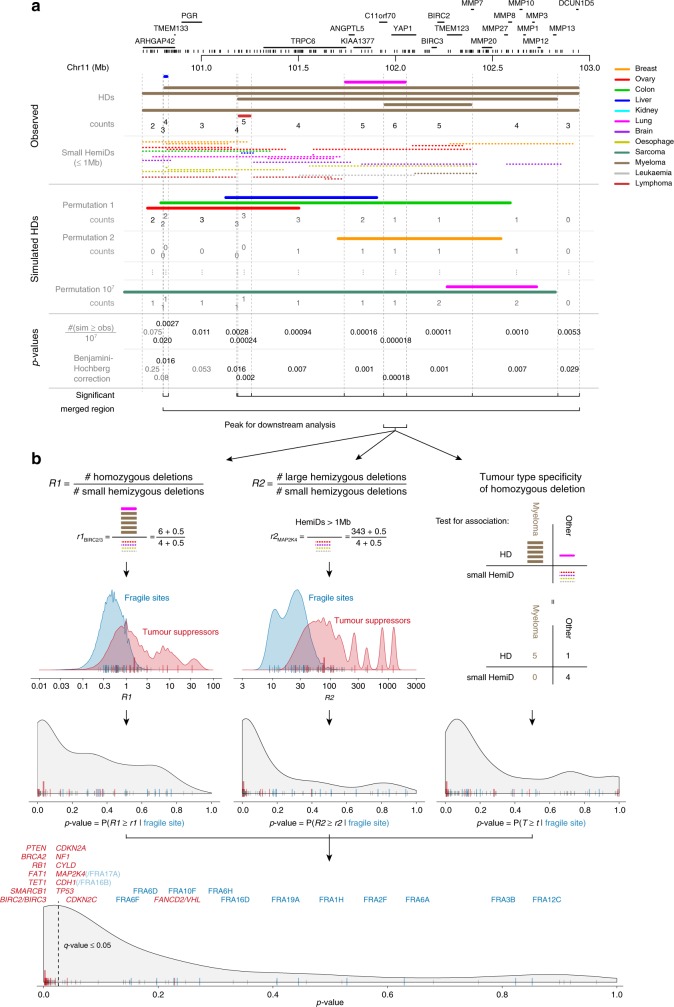
Approach to identify tumour suppressors showing excess homozygous deletions. The approach is illustrated for the *BIRC2/BIRC3* locus. **a** Assessing enrichment of homozygous deletions. The genome is segmented into bins of constant number of observed homozygous deletions by considering all start and end points of every homozygous deletion to be a breakpoint (top). Enrichment is then evaluated over a random model in which the homozygous deletions are shuffled across the genome (permutation strategy 2, middle). For each bin, a *p*-value is calculated as *n*/*M*, where *n* is the number of permutations resulting in at least as many homozygous deletions in the bin as are observed, and *M* the total number of permutations (bottom). *P*-values are adjusted for multiple testing and considered significant when *q* ≤ 0.05. Neighbouring significant bins are merged when they lie within 1 Mb and share >50% of the underlying homozygous deletions. Within each combined region (96 in total), the peak used for downstream analysis is defined as the largest bin with maximal overlap. **b** Statistical model to test for local fragility. Two metrics capture the distinct structural signature of deletions in fragile sites when compared to regions harbouring tumour suppressors: (*R1*) the ratio of homozygous to small (≤1 Mb) hemizygous deletions and (*R2*) the ratio of large to small hemizygous deletions. Note the addition of pseudocounts to avoid zero values in the denominator. Estimated densities of these metrics for fragile sites and tumour suppressors are shown as well as the values obtained for all peaks, except those on the X chromosome (named fragile sites, blue; known tumour suppressors, red; unknown, grey; *BIRC2/BIRC3* large red bar). *P*-values for all peaks are computed under the fragile site null model density. Tumour-type specificity is the third pillar of the model and is tested in a 2 × 2 table of homozygous vs. small hemizygous deletions in the tumour type with the most deletions vs. the other tumour types combined. *P*-values from the three tests are combined and adjusted for multiple comparisons. Local fragility is rejected and the presence of a tumour suppressor considered for peaks with *q* ≤ 0.05

The genomic distribution of homozygous deletions is skewed towards specific regions (Fig. 2a). To further investigate this, two distinct permutation tests were devised. In an initial strategy, we model homozygous deletions as a combination of two independent events, permuting individual loss-of-heterozygosity events in each sample, whereby overlapping regions define homozygous deletions. Based on this test, homozygous deletions, and particularly large homozygous deletions, are strongly depleted across the genome (Fig. 2a, b). This paucity of homozygous deletions is not unexpected, and may be ascribed to negative selection: removal of both copies of any functional gene or other element in the genome likely results in a selective disadvantage for the cell. In a second model, we therefore permuted homozygous deletions as singular events, keeping the total homozygously deleted sequence constant for each sample across permutations. Following this strategy, which is illustrated for the *BIRC2/BIRC3* locus in Fig. 3a, a total of 42.6 Mb of the genome, distributed across 96 distinct regions, was targeted more frequently by homozygous deletions than expected (Supplementary Data 3).

**Fig. 4:**
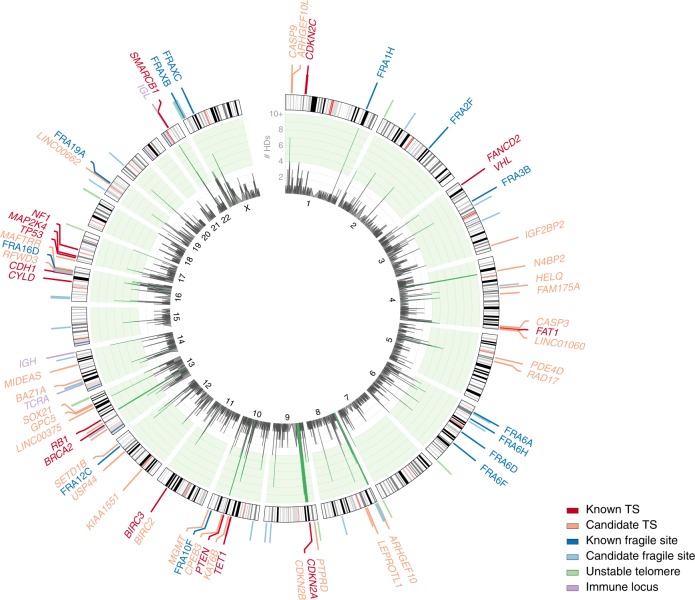
Circos plot of peak regions of homozygous deletions. The inner circle shows the frequency of homozygous deletions, peaks of significant enrichment according to our second permutation model (single event) are coloured green. Assigned peak region classes are colour-coded and indicated on the ideogram (see also Fig. 3). Where applicable, the name of the identified named fragile site, immune locus, or the known or proposed tumour suppressor gene is provided

We observed 15 peaks of homozygous deletions over 16 established tumour suppressors (i.e., genes listed as tumour suppressors in the Cancer Gene Census^16^; Table 2, Fig. 4, Supplementary Fig. 3), a significant enrichment over random expectations (*p* = 0.00103; hypergeometric test). *CDKN2A* was the dominant homozygously deleted tumour suppressor, with 108 homozygous deletions across nine cancer types. Virtually all of these homozygous deletions inactivate the adjacent “backup” tumour suppressor *CDKN2B* as well, rationalising the frequent loss of the combined locus^17^. Homozygous deletions of *CYLD* and *BIRC3* (cIAP2) are found exclusively in multiple myeloma and are both linked to aberrant NF-κB signalling^18^. All *BIRC3* deletions include its adjacent homologue *BIRC2*, consistent with previous observations that only loss of both was shown to induce alternative NF-κB activation in B-cells^19^. Similarly, we observed six homozygous deletions over *VHL*, five of which take out the nearby tumour suppressor *FANCD2* as well. We detected 16 homozygous deletions over *PTEN* in multiple cancer types and 5 homozygous deletions over *NF1*. Homozygous deletions of *RB1* were most frequently found in tetraploid sarcomas. We further observed four homozygous deletions each over *TP53* (three sarcomas), *CDKN2C*, and *FAT1*, and six homozygous deletions each over *MAP2K4* (three breast carcinomas) and *CDH1* (five lung cancers). Homozygous deletions of *SMARCB1* are found only in brain cancers, while homozygous deletions of *TET1* were almost exclusively observed in lung cancer (seven out of eight cases). Interestingly, the four homozygous deletions over *BRCA2* included two sarcomas, a cancer type not previously linked to *BRCA2* mutations.

The 96 regions showing frequent homozygous deletions also include the T-cell receptor alpha locus, as well as both the immunoglobulin heavy and light chain loci (Fig. 4, Supplementary Fig. 4, Supplementary Table 2). Homozygous deletions of these regions are predominantly found in haematological cancers and represent somatic V(D)J recombination events in precursors of normal T-lymphocytes and B-lymphocytes that later developed into tumour cells. These homozygous deletions most likely do not play a role in oncogenesis.

Homozygous deletions also occur frequently over fragile sites, i.e., chromosomal regions showing high rates of breakage. We observed 15 peaks of homozygous deletions over known (named) fragile sites (Fig. 4, Supplementary Fig. 5, Supplementary Table 2).

### Identification of fragile sites and unstable telomeres

Homozygous deletions over tumour suppressors are enriched due to positive selection, whereas homozygous deletions over fragile sites are enriched due to a local increase in genomic instability. Consequently, the structural signature of deletions is distinct in fragile sites, compared to regions harbouring tumour suppressors. Small hemizygous deletions reflect local fragility, as they require two DNA breakage events in close proximity^6^. Large hemizygous deletions, in contrast, are due to several other mechanisms, such as whole-chromosome or whole-arm loss^6^. As a result, fragile sites are characterised by frequent small deletion events, while regions containing tumour suppressors more frequently show large deletion events^6^. This difference was exploited to construct three metrics that discriminate fragile sites from regions containing tumour suppressors (Fig. 3b). The first two capture the structural signature: (*R1*) the ratio of homozygous deletions to small hemizygous deletions over the peak (high for tumour suppressors, low for fragile sites); and (*R2*) the ratio of large hemizygous deletions to small hemizygous deletions (high for tumour suppressors, low for fragile sites). Indeed, both ratios are significantly larger for the identified known tumour suppressors when compared to the known fragile sites (*p* = 3.63 × 10^−3^ and 4.16 × 10^−3^; Fisher–Pitman permutation test). The densities of *R1* and *R2* for fragile sites (or tumour suppressors) can be estimated via a resampling and simulation approach using the known sites, which allows calculation of *p*-values for any peak under a fragile site null model (Methods). In addition, we leveraged tumour-type specificity as a third element in our statistical model. Tumour-type specificity of homozygous deletions can be explained either by selection or by increased genomic instability in the originating tissue or cell type. However, higher genomic instability in a certain tissue or cell type would result in enrichment of both homozygous and small hemizygous deletions in a certain tumour type. Therefore, we can use differences in tumour-type specificity between homozygous and small hemizygous deletions as an indication that tissue-specificity of homozygous deletions is driven by selection (Fig. 3b, right panels). Finally, *p*-values from the three tests are combined through Brown’s method and adjusted for multiple comparisons using Benjamini–Hochberg correction (Fig. 3b, Methods and Supplementary Table 2). Only when the signature of deletions over a peak is unlikely to be explained by local fragility (*q* ≤ 0.05), do we reject the null model and consider the presence of a tumour suppressor.

Our model is able to reject local fragility for 13 of the 15 peaks containing known tumour suppressors (Table 2), *VHL/FANCD2* and *CDKN2C* being the exceptions. Notably, *VHL* shows frequent biallelic inactivation by a hemizygous deletion combined with a point mutation, while homozygous deletions are rare. A possible reason is that for some tumour suppressor genes, homozygous deletions, which often also affect neighbouring genes or regulatory regions, are selected against, whereas point mutations may be better tolerated. Alternatively, a protein may have multiple functions, and while biallelic deletions abolish all functions, point mutations may abolish one while leaving others intact. Two of the established tumour suppressors, *CDH1* and *MAP2K4*, fall within known fragile sites FRA16B and FRA17A, respectively. In both cases, however, our model is able to pick up the signature of positive selection due to a strong signal of tumour-type specificity for *CDH1* and a high ratio of large to small hemizygous deletions for *MAP2K4*.

**Table 2:** Peaks of homozygous deletions over established tumour suppressors and candidate tumour suppressors

Peak region	# of HDs	*p*-value (*q*-value)	Tumour suppressor
*Known tumour suppressors*			
chr1:51.58–53.53	4	4.16 × 10^−2^ (7.42 × 10^−2^)	*CDKN2C*
chr3:10.18–10.20	6	0.229 (0.293)	*FANCD2/VHL*
chr4:187.75–187.90	4	2.57 × 10^−5^ (2.82 × 10^−4^)	*FAT1*
chr9:22.02–22.02	108	1.30 × 10^−3^ (4.38 × 10^−3^)	*CDKN2A(/CDKN2B)* ^a^
chr10:70.05–70.95	8	7.37 × 10^−5^ (6.09 × 10^−4^)	*TET1*
chr10:89.74–89.83	16	6.05 × 10^−9^ (5.51 × 10^−7^)	*PTEN*
chr11:101.95–102.05	6	4.87 × 10^−4^ (1.85 × 10^−3^)	*BIRC3(/BIRC2)* ^a^
chr13:32.92–33.05	4	3.18 × 10^−8^ (1.45 × 10^−6^)	*BRCA2*
chr13:49.04–49.09	23	1.52 × 10^−7^ (4.61 × 10^−6^)	*RB1*
chr16:50.72–50.94	5	2.00 × 10^−3^ (6.11 × 10^−3^)	*CYLD*
chr16:68.64–69.95	6	9.46 × 10^−3^ (2.39 × 10^−2^)	*CDH1*
chr17:7.58–7.58	4	2.08 × 10^−2^ (4.21 × 10^−2^)	*TP53*
chr17:11.96–12.09	6	3.78 × 10^−3^ (1.07 × 10^−2^)	*MAP2K4*
chr17:29.55–29.83	4	2.02 × 10^−3^ (6.11 × 10^−3^)	*NF1*
chr22:24.19–24.48	6	3.29 × 10^−4^ (1.50 × 10^−3^)	*SMARCB1*
*Candidate tumour suppressors*			
chr1:15.90–15.92	7	4.39 × 10^−4^ (1.74 × 10^−3^)	*CASP9*
chr1:17.58–17.63	4	1.99 × 10^−4^ (1.16 × 10^−3^)	*ARHGEF10L*
chr3:185.44–185.53	4	1.85 × 10^−6^ (3.37 × 10^−5^)	*IGF2BP2*
chr4:39.08–39.15	12	8.87 × 10^−4^ (3.23 × 10^−3^)	*N4BP2*
chr4:83.68–83.68	4	1.04 × 10^−6^ (2.36 × 10^−5^)	*HELQ/FAM175A*
chr4:185.60–185.65	4	2.43 × 10^−4^ (1.16 × 10^−3^)	*CASP3*
chr4:189.47–190.50	5	1.04 × 10^−2^ (2.56 × 10^−2^)	*LINC01060*
chr5:58.41–58.41	4	4.00 × 10^−4^ (1.66 × 10^−3^)	*PDE4D* ^b^
chr5:68.40–68.69	6	9.32 × 10^−4^ (3.26 × 10^−3^)	*RAD17*
chr8:1.77–1.94	8	4.68 × 10^−6^ (7.09 × 10^−5^)	*ARHGEF10*
chr8:29.97–29.98	4	1.63 × 10^−2^ (3.72 × 10^−2^)	*LEPROTL1*
chr9:9.42–9.64	5	7.36 × 10^−3^ (1.97 × 10^−2^)	*PTPRD*
chr10:76.72–76.81	5	3.59 × 10^−5^ (3.27 × 10^−4^)	*KAT6B*
chr10:93.99–94.03	5	1.91 × 10^−4^ (1.16 × 10^−3^)	*CPEB3*
chr10:131.42–131.49	5	1.74 × 10^−4^ (1.16 × 10^−3^)	*MGMT*
chr12:32.15–32.24	5	1.29 × 10^−2^ (3.01 × 10^−2^)	*KIAA1551*
chr12:95.88–96.27	4	2.01 × 10^−2^ (4.16 × 10^−2^)	*USP44*
chr12:122.30–122.37	6	2.21 × 10^−5^ (2.82 × 10^−4^)	*SETD1B*
chr13:85.51–85.66	4	1.81 × 10^−2^ (3.97 × 10^−2^)	*LINC00375*
chr13:92.45–92.45	10	2.16 × 10^−4^ (1.16 × 10^−3^)	*GPC5*
chr13:95.39–95.46	4	2.35 × 10^−4^ (1.16 × 10^−3^)	*SOX21*
chr14:35.09–35.32	6	1.94 × 10^−2^ (4.11 × 10^−2^)	*BAZ1A*
chr14:74.07–74.55	4	2.55 × 10^−2^ (4.95 × 10^−2^)	*MIDEAS*
chr16:74.65–74.69	5	4.65 × 10^−3^ (1.28 × 10^−2^)	*RFWD3*
chr16:79.80–79.80	4	7.58 × 10^−3^ (1.97 × 10^−2^)	*MAFTRR*
chr19:28.14–28.15	6	1.18 × 10^−2^ (2.83 × 10^−2^)	*LINC00662*

*Note*: Each region’s genomic position is shown, the number of homozygous deletions (HDs), the combined *p*-value (and multiple testing-corrected *q*-value) indicating the probability that the enrichment in homozygous deletions is due to increased genomic instability (rather than due to positive selection), and the established or candidate tumour suppressor gene identified

^a^*CDKN2B* and *BIRC2* are candidate tumour suppressor genes with a high level of evidence. They are always lost together with *CDKN2A* and *BIRC3*, respectively, and are likely to contribute to positive selection of the homozygous deletions

^b^*PDE4D* shows intragenic homozygous deletions, suggesting that these deletions may be gain-of-function rather than loss-of-function mutations

Among the remaining 65 regions showing recurrent homozygous deletions, our statistical model identified 33 additional fragile sites. These include 24 fragile sites not involving telomeres (Supplementary Fig. 6), a sizable subset of which has been described previously^6^, as well as 9 (sub)telomeres showing enrichment of both homozygous and hemizygous deletions (Supplementary Fig. 7).

**Fig. 5:**
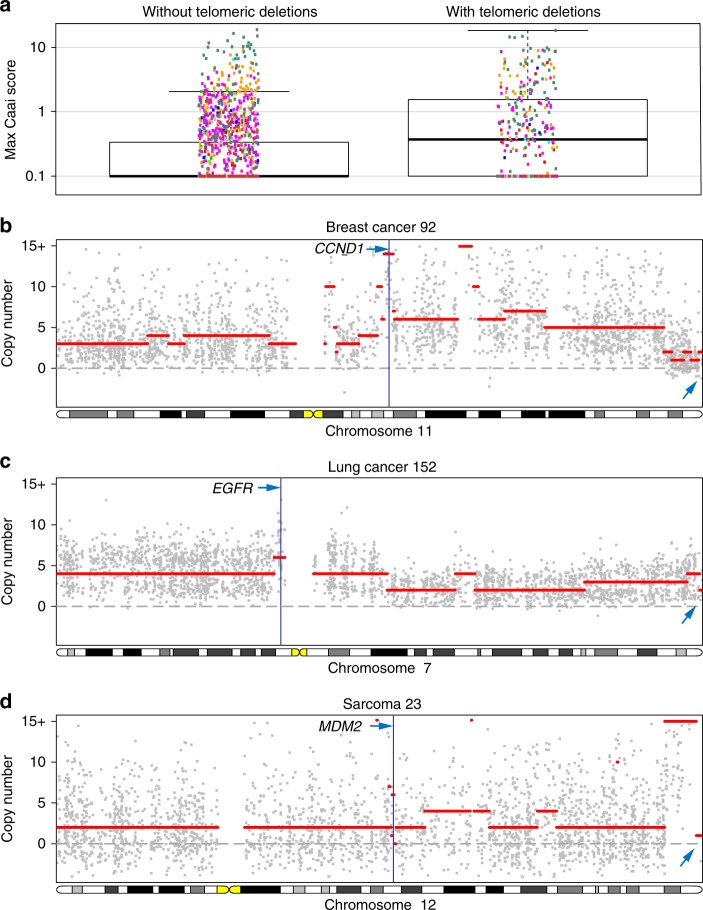
Tumours with telomeric losses show more complex (oncogenic) rearrangements. **a** Maximum CAAI scores, quantifying the presence of regions with complex rearrangements^24^, for tumours with and without (sub)telomeric deletions. The increased genomic complexity in tumours with (hemizygous or homozygous) telomeric deletions is likely the product of breakage-fusion-bridge cycles initiated by these telomeric deletions. **b**–**d** Examples of amplified oncogenes in regions with high CAAI scores on chromosomes with telomeric deletions

Hemizygous and homozygous deletions in these (sub)telomeric regions represent the signature of a prior telomere crisis triggering genomic instability. Ongoing cell division in the absence of telomerase expression leads to telomere attrition and subsequent removal of subtelomeric sequences. The exposed chromosome ends are often resolved by end-to-end chromosome fusions, with further loss of subtelomeric sequences^20^. The resulting dicentric chromosome is mitotically unstable and sparks a number of breakage-fusion-bridge cycles in the subsequent cell divisions or catastrophic events such as chromothripsis and kataegis, creating increasing numbers of subclones^21–23^. These serve as a substrate for natural selection, enabling significant oncogenic steps within a limited time. Although, by themselves, the observed subtelomeric deletions are most likely not oncogenic, they may represent archaeological traces of oncogenic events initiated by telomere loss. Breakage-fusion-bridge cycles often cause complex local copy number alterations. We indeed observed that tumours with hemizygous or homozygous (sub)telomeric losses show more complex rearrangements compared to cases that do not show such losses (*p* < 1 × 10^−15^; Complex Arm-wise Aberration Index (CAAI) scores^24^; Fig. 5a). These complex copy number aberrations often harbour amplified oncogenes likely contributing to oncogenesis (Fig. 5b–d).

### Identification of candidate tumour suppressors

**Fig. 6:**
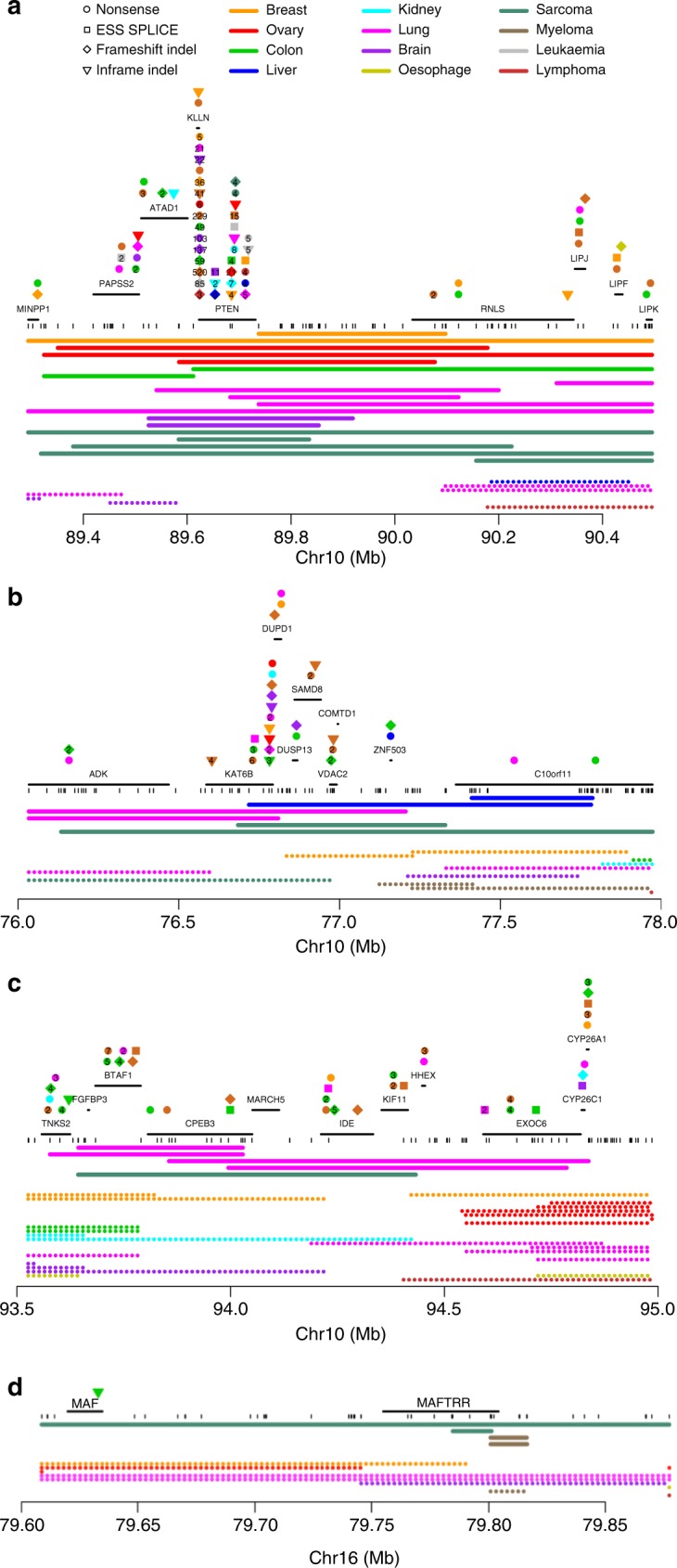
Examples of tumour suppressors targeted by homozygous deletions. **a** Known tumour suppressor *PTEN*; **b**–**d** candidate tumour suppressors identified in this study. **b** *KAT6B*, **c** *CPEB3*, and **d** *MAFTRR*. Positions of genes are indicated as well as truncating mutations annotated in COSMIC^25^, coloured according to tumour type and with symbols showing the mutation type. When multiple somatic mutations in the same tumour type are annotated close together in COSMIC, their numbers are shown. Array probe positions are depicted below the genes. The minimal regions of homozygous deletions are shown as bold lines and small hemizygous deletions as dotted lines, both colour-coded by tumour type. Homozygous deletions are unlikely to extend more than two array probe positions beyond the indicated segments (*p* = 0.015, see Methods)

In addition to the 15 regions containing established tumour suppressors, our model identified 32 regions that are unlikely to be explained by local fragility, showing a signature of positive selection (Table 2, Supplementary Table 2). We overlaid the patterns of homozygous deletions in these regions with mutation data from COSMIC^25^ and with scientific literature, aiming to identify candidate tumour suppressors. Two regions were intergenic, and for four additional regions the targets remain unknown (Supplementary Fig. 8). In each of the remaining 26 regions, we were able to pinpoint at least one candidate tumour suppressor (Fig. 6 and Supplementary Fig. 9). Our proposed tumour suppressors include genes previously suggested to play a role in oncogenesis (e.g., *MGMT* and *USP44*), as well as novel candidates (e.g., *KIAA1551*, *CASP3*, and *MAFTRR*).

Our results further establish several genes proposed in the literature to be tumour suppressors. In lung and kidney cancers, we found homozygous deletions of the *PTPRD* gene, encoding receptor-type tyrosine-protein phosphatase delta, previously suggested to be a tumour suppressor^26^. Another peak of homozygous deletions targeted the ubiquitin-specific protease *USP44*, specifically in lung cancer. Interestingly, USP44 was recently found to regulate the mitotic cell cycle checkpoint and *Usp44* knockout mice spontaneously formed tumours, particularly in the lungs^27^. Homozygous deletions over or near *SOX21* were found in three cases of multiple myeloma, one lymphoma and one breast cancer. SOX21 is a modulator of the effects of the oncogene and pluripotency transcription factor SOX2 on cell identity^28^. *SOX21* was recently proposed to act as a tumour suppressor in glioma, after observations that the SOX2:SOX21 balance determines cellular choice between a stem-like state and differentiation^29^. In addition, we found homozygous deletions targeting the histone H3K23 acetyltransferase *KAT6B* that, combined with a series of truncating mutations^25^, implicate this gene as a tumour suppressor. *KAT6B* was recently proposed as a recurrently homozygously deleted tumour suppressor in small cell lung cancer^30^. Indeed, we observe two homozygous deletions in lung cancer, but also two homozygous deletions each in liver cancer and sarcoma, suggesting *KAT6B* may function as a tumour suppressor in multiple cancer types. Likewise, we identified homozygous deletions within *GPC5*, encoding the heparin sulphate proteoglycan Glypican-5. *GPC5* was recently reported to be an epigenetically silenced tumour suppressor in lung adenocarcinoma, where it binds Wnt3a at the cell surface to inhibit Wnt/β-catenin signalling^31^. Interestingly, we observe homozygous deletions not only in lung cancer, but also in ovarian and liver cancer, supporting a tumour suppressive role in a wider range of cancer types. *BAZ1A* is a component of the ISWI-family chromatin remodelling complexes ACF and CHRAC. It is frequently mutated in gynaecological carcinosarcoma^32^ and to a lesser extent in ovarian cancer^33^. While copy number losses of *BAZ1A* are known to occur in renal papillary carcinoma, we observe homozygous deletions in lung cancer and sarcoma^34^. Another peak of homozygous deletions targets *CPEB3*, mostly in lung cancer. *CPEB3* encodes a sequence-specific RNA-binding protein that was proposed to function as a tumour suppressor by transcriptionally repressing *EGFR*^35,36^. Therefore, our results suggest that, in addition to amplification of *EGFR*, lung cancers can also adopt homozygous deletions of *CPEB3* as an alternative mechanism to overexpress *EGFR*. This raises the possibility that EGFR-targeted therapy may also be effective in patients with homozygous deletions of *CPEB3*.

Our screen identified six genes involved in DNA damage response and repair. *MGMT*, encoding the extensively studied DNA-repair enzyme O^6^-methyl-guanine-DNA methyltransferase, is frequently inactivated in gliomas by promoter methylation, and this is an important marker for therapy response^37^. Homozygous deletion may represent an alternative mechanism to inactivate this DNA repair gene in cancer cells. *RAD17* is involved in DNA damage repair and acts as a cell cycle checkpoint gene. An RNAi screen indicated that Rad17 acts as a haploinsufficient tumour suppressor in a mouse lymphoma model^38^. *N4BP2*, also called *B3BP*, encodes a binding partner of the p300/CBP histone acetyltransferase and the Bcl-3 oncogene. N4BP2 shows 5′-polynucleotide kinase and DNA nicking endonuclease activities and has been proposed to play a role in DNA repair or recombination^39^. *RFWD3* encodes a ubiquitin ligase that acts as a positive regulator of TP53 stability in response to DNA damage^40^ and loss of *RFWD3* results in persistent DNA damage^41^. *HELQ* is a DNA helicase involved in replication-coupled DNA repair that has previous evidence of a tumour suppressor function^42^. Interestingly, the same peak region of homozygous deletions also contains *FAM175A* (also called *ABRAXAS*), a suggested tumour suppressor gene that encodes a protein component of the BRCA1–A complex that leads the BRCA1-BARD1 heterodimer to sites of DNA double-strand breaks, targeting these for homologous recombination^43,44^. We therefore hypothesise that homozygous deletions of this region on chromosome 4 coordinately inactivate two tumour suppressor genes, involved in different DNA damage repair pathways.

We also find candidate tumour suppressors that are related to known cancer genes. We detected homozygous deletions affecting *CASP3* and *CASP9*, or regions close by, in various cancer types. Both genes encode pro-apoptotic and anti-apoptotic splice isoforms of the respective cysteine/aspartic acid proteases^45,46^. *CASP8*, another member of the caspase family, is a tumour suppressor in breast cancer^4^. The pro-apoptotic isoforms of caspase-8 and caspase-9 induce apoptosis through cleavage of caspase-3. It is therefore likely that perturbation of any of these three caspase genes may abrogate apoptosis. We detected a peak of homozygous deletions in lung cancer close to *SETD1B*, a member of a family of histone methyltransferases that also includes *SETD2*, known to be involved in renal carcinoma. SETD1B specifically methylates H3K4, thereby playing a role in epigenetic control of transcription. We identified several large and smaller homozygous deletions targeting *ARHGEF10*, a Rho guanine nucleotide exchange factor regulating mitotic spindle formation that has been proposed as a tumour suppressor^47,48^. Interestingly, we also observed homozygous deletions affecting its closest paralog *ARHGEF10L*, suggesting that both genes may be tumour suppressors.

Some homozygous deletions may drive oncogenesis through other mechanisms than inactivation of a tumour suppressor gene. We observed intragenic deletions of *PDE4D*, encoding phosphodiesterase 4D. *PDE4D* has previously been reported to be targeted by internal microdeletions that are hypothesised to function as tumour-promoting factors^49^. Therefore, these homozygous deletions may represent gain-of-function rather than loss-of-function mutations.

Several peaks of homozygous deletions point to genes not previously implicated as tumour suppressors, e.g., *LEPROTL1*, *KIAA1551*, *MIDEAS*, *MAFTRR*, and *IGF2BP2*. Together with its homologue Leptin Receptor Overlapping Transcript (*LEPROT*), *LEPROTL1* negatively regulates leptin receptor surface expression and thus the response to leptin, a pleiotropic hormone^50,51^. Lung tissues both produce and respond to leptin, and leptin is required for proliferation of various non-small cell lung cancer cell lines, at least in part due to activation of downstream Notch and JAK/STAT signalling^52^. Four homozygous deletions in lung cancer suggest a role for *LEPROTL1* in keeping this feedback loop in check in normal lung cells. MIDEAS is part of a recently discovered class I histone deacetylase complex, dubbed MiDAC (Mitotic Deacetylase Complex)^53^. The complex associates with cyclin A and is upregulated in cells blocked in mitosis^54^. While the co-repressor MIDEAS couples inositol phosphate signalling to activation of histone deacetylation, its precise role in cell cycle regulation is still unknown^55^. Two homozygous deletions each in myeloma and sarcoma targeted *MAFTRR* (MAF transcriptional regulator RNA), a long intergenic noncoding RNA gene. MAFTRR was recently shown to recruit chromatin modifiers LSD1 and EZH2 to the upstream *MAF* oncogene in a long-distance chromatin interaction, downregulating its expression^56^. *MAF* overexpression is a frequent oncogenic event in multiple myeloma, stimulating cell cycle progression and altering bone marrow stromal interactions^57^. Note that one homozygous deletion in sarcoma abolishes both *MAF* and *MAFTRR*, suggesting *MAFTRR* may still have other functions. Aside from *MAFTRR*, we identify three additional lncRNA genes as potential tumour suppressors: *LINC01060*, *LINC00375*, and *LINC00662*. Unfortunately, their precise functions are yet to be elucidated. *IGF2BP2* (also known as *IMP2*) encodes a post-transcriptional modulator implicated in mRNA localisation, stability, and translational control. It is part of the frequently aberrated IGF/PI3K-AKT/mTOR pathway and controls the translation of mitochondrial mRNAs^58,59^. Depletion of *IGF2BP2* decreases oxygen consumption while increasing mitochondrial mRNA translation and possibly mitochondrial biogenesis^58^. Our observation of a peak of homozygous deletions suggests it could act as a rare tumour suppressor, controlling a switch in cancer nutrient and energy metabolism.

## Discussion

In this study, we aimed to identify rare tumour suppressors through a systematic pan-cancer analysis of homozygous deletions in primary tumours. Our screen detected 16 established tumour suppressors, 3 immune regions, 15 known (named) fragile sites, 24 additional intrachromosomal fragile sites, 9 regions of telomeric instability, and 32 regions showing signatures of positive selection for homozygous deletions (Fig. 4). For 26 of the latter regions, we were able to propose at least one candidate tumour suppressor.

We developed a statistical model to test if enrichment of homozygous deletions in a region can be explained by local fragility alone, or whether there may be positive selection (Fig. 3). As illustrated by the identification of *CDH1*/FRA16B and *MAP2K4*/FRA17B, two tumour suppressors located within known fragile sites, these properties are not always biologically separated, yet in both cases, our model was able to discern the signature of selection. For other known tumour suppressor loci, *CDKN2C* and *FANCD2*/*VHL*, our model could not identify this signature. *VHL* is only rarely targeted by homozygous deletions and is typically biallically inactivated by a point mutation combined with loss-of-heterozygosity of the other allele. It is therefore likely that *VHL* shows stronger positive selection for hemizygous deletions than homozygous deletions. As a result, our model cannot detect a signature of positive selection for homozygous deletion. *PTPRD* has been suggested to be a tumour suppressor^26^, while other reports indicate this is a fragile site^6^. Our results are consistent with the former, in large part due to the specificity of homozygous deletions in lung and kidney cancers (absent from the pattern of small hemizygous deletions). Particularly in kidney cancer, homozygous deletions are very rare: we only detect 25 in 73 cases, 3 of which overlap *PTPRD*.

Our model is considerably more conservative on the X chromosome (see Methods) and we do not infer any tumour suppressors on X. Out of eight homozygous deletions affecting *DMD* (encoding dystrophin), we observe seven in sarcomas. Although *DMD* is the largest gene in the human genome and is part of known fragile site FRAXC, this suggests homozygous deletions of *DMD* may play a role in sarcoma. Indeed, *DMD* was recently validated as a tumour suppressor and anti-metastatic factor in myogenic sarcomas^60^ and dystrophin/dysferlin double mutant mice show a high incidence of rhabdomyosarcoma^61^. We also observe a peak of homozygous deletions targeting *MXRA5*, a poorly studied gene that was recently suggested to be frequently mutated in non-small cell lung carcinoma^62^. While we are unable to reject local fragility as the underlying cause of both peaks, this may be due to lack of power, and further studies are needed to conclusively determine if these genes play a tumour suppressive role in some cancers.

A previous pan-cancer study performed GISTIC analysis^63^ to identify recurrent focal gains and losses^64^, some of which could be attributed to oncogenes and tumour suppressors. Due to our strict focus on homozygous deletions rather than focal losses in general, we aim to identify tumour suppressors recurrently targeted by two independent copy number changes. This approach is not well suited to identify haploinsufficient tumour suppressors or tumour suppressors showing mainly recurrent point mutations combined with copy number losses of the other allele, as discussed above for *VHL*. However, both our study (on 2218 cancers) and the Zack et al. study on a larger number of cases (4934) identify the same 13 known tumour suppressors (we additionally identify *TET1* and *BIRC3*), suggesting our focused approach is competitive at identifying tumour suppressors.

Our results provide a view on the landscape of tumour suppressors that is complementary to sequencing screens for recurrent single-nucleotide substitutions and small insertions and deletions. Many genes frequently targeted by inactivating mutations in cancers do not seem to be frequently targeted by homozygous deletions. This is exemplified by *TP53*, found mutated in a very high proportion of cases across cancer types, but homozygously deleted in less than 0.2% of cancers in this study. Other known tumour suppressors, such as *RB1* and *PTEN*, can readily be inactivated by both homozygous deletions and inactivating point mutations (often in combination with loss-of-heterozygosity of the other allele). Adjacent tumour suppressor pairs (e.g., *CDKN2A*/*CDKN2B*, *BIRC2*/*BIRC3*, *HELQ/FAM175A*, …) may be especially rewarding targets for homozygous deletion in some cases, as two mutation events can abolish all tumour suppressor activity. We conjecture that this study identifies a class of predominantly rare tumour suppressors, such as *CPEB3* and *MGMT*, that are more prone to be inactivated by homozygous deletions than point mutations, a proportion of which therefore may not be readily identifiable through mutation analysis given current sample sizes.

## Methods

### Copy number analysis of 2218 primary tumour samples

SNP array studies were selected from the CaSNP database^65^, the MetaCGH database^66^, and from literature searches, with the aim of collecting all studies of primary tumours performed on Affymetrix 250K StyI arrays with publically available raw data. All pre-cancerous lesions and metastases were excluded. Pairwise comparison of all samples with Pearson correlation was used to identify duplicate samples (Pearson correlation above 0.8), in which case duplicates were removed. Samples were stratified into 12 broadly defined tumour types: breast cancer, ovarian cancer, colorectal cancer, hepatocellular carcinoma, renal cancer, lung cancer, cancers of the brain and nervous system, oesophageal cancer, sarcoma, multiple myeloma, leukaemia and lymphoma, each containing >50 samples. Other tumour types with fewer samples available were excluded. Our final data set included 2218 unique primary tumour samples from 27 different studies (Supplementary Table 1, Supplementary Data 1). As the samples from each cancer type in our compendium are derived from multiple centres, cross-type comparisons are less likely to be affected by local ascertainment biases and referral procedures.

Total copy number (LogR) and B allele frequency (BAF) values were obtained from CEL files using PennCNV-Affy^67^. GRCh37/hg19 probe annotation (version 32) was obtained from the Affymetrix website (http://www.affymetrix.com). Only probes that mapped to unique locations of the genome were retained. GC wave correction was performed as previously described^68^ and as implemented in ASCAT 2.2^10^. Copy number analysis was performed using ASCAT^10^ version 2.2, with default parameters for tumour samples without matched normal data. The default value *γ* = 0.55 was used in ASCAT and has previously been shown to fit the Affymetrix data well. In order to remove any adverse influence of germline copy number variants (CNVs), all SNPs within known germline CNVs were removed prior to ASCAT analysis. Positions of known germline CNVs were obtained from the Database of Genomic Variants^69^, version hg19.v10, from which all CNVs identified on Affymetrix 500K and SNP6 platforms were selected. This reduced the number of analysed probes from 228,586 to 208,786.

### Identification of regions with candidate tumour suppressors

*Step 1: Identification of hotspots of homozygous deletions*: After ASCAT analysis, homozygous deletions were straightforwardly identified as regions having zero copies of both alleles in the tumour cells, each region extending from the position of the first probe being homozygously deleted to the last probe being homozygously deleted. Therefore, the reported positions of a homozygous deletion can be considered the minimal region that is homozygously deleted, as supported by the SNP array data of that sample. The genome was then segmented into bins of constant number of observed homozygous deletions by considering all start and end points of every homozygous deletion to be a breakpoint. Each bin thus defined was then associated with the total number of homozygous deletions over that region observed across all samples (Fig. 3a).

To determine the significance of the number of homozygous deletions in each bin, a permutation test was performed. We initially reasoned that each observed homozygous deletion is the result of two distinct hemizygous deletion events, and we therefore treated both events separately in the simulations. We identified within each parental chromosome and within each sample all hemizygously and homozygously deleted regions. Each such region represents the result of a hemizygous deletion event, and a homozygous deletion occurs when two such regions overlap within a sample. Deletions were classified into two categories, “small” and “large”, and the positions of the small deletions were randomly assigned to a new position in the genome (treating all chromosomes as one unit, but ensuring that each deletion fits within the borders of one chromosome), while the large deletions were kept fixed in their original positions. For parental chromosomal deletions being part of a homozygous deletion, the small and large deletions are readily identified by comparing their sizes. For parental chromosomal deletions not being part of a homozygous deletions (i.e., the true hemizygous deletions), a classifier was applied to determine whether the deletion was small or large. This classifier was a 2-component mixture model trained on the small and large deletions being a part of a homozygous deletion, and resulted in a probability of being a small deletion which was then used to decide whether the position of the deletion should be kept fixed or randomly drawn in the permutation. Finally, a *p*-value was calculated for each bin as *n*/*M*, where *n* was the number of permutations resulting in at least as many homozygous deletions in the bin as were observed, and *M* the total number of permutations (in our study, *M* = 10^7^).

Even with the larger deletions in fixed positions, this permutation strategy indicated that the incidence of simulated homozygous deletions was much higher than the observed rate of homozygous deletions across the whole genome (Fig. 2a). In addition, the size distribution of homozygous deletions showed a longer tail of large homozygous deletions than those actually observed (Fig. 2b). This indicates that in most regions of the genome of tumour (and normal) cells, there is strong negative selection against homozygous deletions: for many genes, removal of both copies results in a selective disadvantage of the cell in which this occurs. For that reason, the simulations above were deemed unrealistic, and a second permutation strategy was devised.

In our second permutation strategy (Fig. 3a), we aimed to model the rate of homozygous deletions according to the observed rate, as well as keep the size distribution equal to that observed. In each permutation, the homozygous deletions in every sample were randomly assigned to a new position in the genome (treating all chromosomes as one unit, but ensuring that each homozygous deletion fits within the borders of one chromosome). For each bin, a *p*-value was calculated as *n*/*M*, where *n* was the number of permutations resulting in at least as many homozygous deletions in the target bin as were observed, and *M* was the total number of permutations (in our study, *M* = 10^7^). *P*-values were adjusted for multiple comparisons by applying the Benjamini–Hochberg procedure. Bins were considered significant if their false discovery rate-adjusted *p*-value (i.e., *q*-value) ≤0.05. Neighbouring bins with a significant *q*-value were merged when they lay within 1 Mb of one another and when they had more than half of their underlying homozygous deletions in common (e.g., when four out of five HDs were shared). When they were separate peaks (e.g., overlaps of 5-4-3-2-3-4-5) they were kept as such. In line with this rule, non-adjacent bins were merged in the following regions: chr4:39.08–39.15, chr4:182.34–182.70, chr9:133.39–133.62, chr11:101.95–102.05 (Fig. 3a), chr14:26.40–28.04, chr16:6.74–6.76, chr18:76.71–77.80, and chrX:3.22–4.12. In all other cases, only directly adjacent significant bins were merged. Within each of the resulting 96 combined regions enriched in homozygous deletions, the position of the homozygous deletion peak was defined as the largest region of maximal overlap. Only the deletions overlapping this peak are used as input to the models in Step 2.

To assess whether the list of genes affected by recurrent homozygous deletions is enriched for known tumour suppressors, we performed the following hypergeometric test. Considering there are 168 known tumour suppressors (Cancer Gene Census v79), in a total of 30,382 protein-coding/lincRNA/miRNA genes (ensemble GRCh37 release 85), we computed the probability of observing at least as many known tumour suppressors as we do (≥16) among the total number of genes falling within a 1 Mb window around the centre of an enrichment peak (1197).

*Step 2: Identifying peaks containing tumour suppressors*: A statistical model was constructed quantifying the probability that the observed signature of deletions in a given peak (i.e., the peak in a significant homozygous deletion region from step 1) can be ascribed to local fragility alone (Fig. 3b). The approach involves the calculation of *p*-values using three different metrics under the same overall hypothesis of local fragility (*H*_0_). Each metric captures a largely orthogonal structural or biological feature of the peak, and the three *p*-values are combined into one final meta-analysis *p*-value (Fig. 3b). Note that throughout this model, small hemizygous deletions were defined as regions of loss of heterozygosity ≤1 Mb.

The first two metrics capture the structural signature. They are: *R1*, the ratio of homozygous deletions to small hemizygous deletions, and *R2*, the ratio of large (>1 Mb) to small hemizygous deletions over the peak (Fig. 3b). The densities of random variables *R1* and *R2* for fragile sites (or tumour suppressors) are estimated via a resampling and simulation approach in which we sample 10^7^ times with replacement from the known sites and simulate a number of homozygous, small and large hemizygous deletions according to a multinomial distribution ${\cal M}\left( {n,\, \vec{\bf{ p}}} \right)$ with *n* the cohort size and $\vec{\bf{ p}} = \left( {p_{{\rm{obs}}}^{{\rm{HD}}},\,p_{{\rm{obs}}}^{{\rm{hemiD}}\, \le \,{\rm{1Mb}}},\,p_{{\rm{obs}}}^{{\rm{hemiD}}\,{\rm{ >}}\,{\rm{1Mb}}},\,p_{{\rm{obs}}}^{{\rm{noD}}}} \right)$ the vector of the observed rates of each type at the sampled site (homozygous, small and large hemizygous deletions, and no deletions; note that $p_{{\rm{obs}}}^{{\rm{HD}}} + p_{{\rm{obs}}}^{{\rm{hemiD}}\, \le \,{\rm{1Mb}}} + p_{{\rm{obs}}}^{{\rm{hemiD}}\,{\rm{ >}}\,{\rm{1Mb}}} + p_{{\rm{obs}}}^{{\rm{noD}}} = 1$). *P*-values for any given peak can then be calculated as *P*(*R1* ≥ *r1*) and *P*(*R2* ≥ *r2*) using the estimated fragile site density as a null model (*r1* and *r2* refer to the values of *R1* and *R2* observed for that peak). As a third metric, we leveraged tumour-type specificity of homozygous deletions vs. small hemizygous deletions (Fig. 3b). A one-sided Fisher–Boschloo exact unconditional test for association is performed on a 2 × 2 table of the number of homozygous against hemizygous deletions and the tumour type showing the highest number of deletions (at least one homozygous) against the other tumour types combined.

The metrics above generated three *p*-values for each homozygous deletion peak. An empirical adaptation of Brown’s method (an extension of Fisher’s method which takes correlation between the test statistics into account) was then used to combine these partially correlated *p*-values into a single *p*-value for each homozygous deletion peak^70^ and the Benjamini–Hochberg method was used for multiple testing correction. Only when the signature of deletions in a peak is unlikely to be explained by local fragility (*q* ≤ 0.05) do we consider the presence of a tumour suppressor in the region.

To avoid biases, the two structural models (*R1* and *R2*) were not applied to the X chromosome and tumour-type specificity was assayed only on the cancers in females. Therefore, power to separate fragile sites from tumour suppressors is considerably reduced on X. FRA16B and FRA17A were excluded for density estimation of the fragile site null model as they contain tumour suppressors *CDH1* and *MAP2K4*, respectively. To avoid infinite values of *R1* and *R2*, a pseudocount of 0.5 was added to each of the observed counts.

*Step 3: Annotation and identification of tumour suppressors*: Known mutations in the genes up to 1 Mb around the peak regions were obtained from COSMIC^25^, version 62. Only mutations that can give rise to a truncated protein were selected: nonsense mutations, essential splice site mutations, frameshift and in-frame insertions and deletions. Established tumour suppressors were obtained from the Cancer Gene Census v79, where they are annotated as tumour suppressor genes and/or recessive cancer genes^16^. In each homozygous deletion region showing a signature of selection as inferred from step 2, genes up to 1 Mb around the window were evaluated as candidate tumour suppressors based on the patterns of homozygous deletions, literature support, and the annotated COSMIC mutations. When assessing the pattern of homozygous deletions, we employed a conservative estimate of the maximal size of the deletions. We assume ASCAT has identified the optimal segmentation given the data. However, random (Gaussian) noise in the signal may result in “misclassification” of a homozygously deleted array probe as being part of the adjacent segment. We computed this probability as follows. For each homozygously deleted segment and its adjacent segments on the same chromosome, we obtained robust estimates of the mean and standard deviation of the LogR signal (LogR was preferred as it is more informative than BAF in homozygously deleted regions). The probability of an array probe on the homozygously deleted segment to be wrongly assigned to the adjacent segment was then calculated as the corresponding overlap between the normal distributions of the signal of both segments. Finally, the probability of the homozygously deleted segment extending 1, 2, 3, …, array probe positions into the adjacent segments was computed using a negative binomial distribution. Results show that homozygous deletions are unlikely to extend more than two array probe positions beyond the identified minimal segments (*p* = 0.015).

### Code availability

The R code that was used to run simulations, compute statistics, and generate figures is publically available at https://github.com/jdemeul/HomDels.

### Data availability

The Affymetrix 250K StyI array data that support the findings of this study are described in the studies listed in Supplementary Table 1. They are available in the repositories and with the identifiers indicated in Supplementary Data 1.

## Electronic supplementary material

Supplementary Information

Description of Additional Supplementary Files

Supplementary Data 1

Supplementary Data 2

Supplementary Data 3
