# Author Correction: Pan-cancer analysis of homozygous deletions in primary tumours uncovers rare tumour suppressors

**DOI:** 10.1038/s41467-019-08512-7

**Published:** 2019-01-28

**Authors:** Jiqiu Cheng, Jonas Demeulemeester, David C. Wedge, Hans Kristian M. Vollan, Jason J. Pitt, Hege G. Russnes, Bina P. Pandey, Gro Nilsen, Silje Nord, Graham R. Bignell, Kevin P. White, Anne-Lise Børresen-Dale, Peter J. Campbell, Vessela N. Kristensen, Michael R. Stratton, Ole Christian Lingjærde, Yves Moreau, Peter Van Loo

**Affiliations:** 1 Department of Electrical Engineering (ESAT) and iMinds Future Health Department, University of Leuven, Kasteelpark Arenberg 10, B-3001 Leuven, Belgium; 2 Department of Genetics, Institute for Cancer Research, Oslo University Hospital Radiumhospitalet, N-0310 Oslo, Norway; 3 The Francis Crick Institute, 1 Midland Road, NW1 1AT London, UK; 4 Department of Human Genetics, University of Leuven, Herestraat 49, B-3000 Leuven, Belgium; 5 Wellcome Trust Sanger Institute, Hinxton, CB10 1SA Cambridge, UK; 6 Big Data Institute, University of Oxford, Old Road, OX3 7LF Oxford, UK; 7 Institute for Genomics and Systems Biology, University of Chicago, 900 East 57th Street, 60637 Chicago, IL USA; 8 Committee on Genetics, Genomics, and Systems Biology, University of Chicago, 920 East 58th Street, 60637 Chicago, IL USA; 9 Department of Pathology, Oslo University Hospital Radiumhospitalet, N-0310 Oslo, Norway; 10 Department of Informatics and Centre for Cancer Biomedicine, University of Oslo, N-0424 Oslo, Norway; 11 Department of Ecology and Evolution, University of Chicago, 1101 East 57th Street, 60637 Chicago, IL USA; 12 Department of Human Genetics, University of Chicago, 920 East 58th Street, 60637 Chicago, IL USA; 13 Tempus Labs, Inc, Chicago, IL USA

Correction to: *Nature Communications* 10.1038/s41467-017-01355-0; published online: 31 October 2017.

The original version of this Article omitted a declaration from the competing interests statement, which should have included the following: ‘K.P.W. is President of Tempus Lab, Inc., Chicago, IL, USA’. This has now been corrected in both the PDF and HTML versions of the Article.

