# Supplementary material for: Pan-cancer analysis of homozygous deletions in primary tumours uncovers rare tumour suppressors
Source: Nat Commun. 2017 Oct 31;8:1221. doi: 10.1038/s41467-017-01355-0 (PMC5663922; doi:10.1038/s41467-017-01355-0)
Supplement: Supplementary file 1 — Supplementary Information [file 41467_2017_1355_MOESM1_ESM.pdf]

Supplementary Fig. 1: Overview of ASCAT analysis results

Aberrant and nonaberrant samples after ASCAT analysis across 12 cancer types, and cases that failed ASCAT analysis. 81 samples (3.6%) failed ASCAT analysis and are not included in this figure and in any subsequent analyses. Of the 2,137 cases that passed ASCAT analysis, 273 (12.8%) showed little to no copy number aberrations, and therefore purity estimates can be considered less accurate. These samples are therefore not included in these plots. However, as we observed clear driver homozygous deletions in known tumour suppressor CDKN2A in 6 of these cases, they are included in all further analyses.

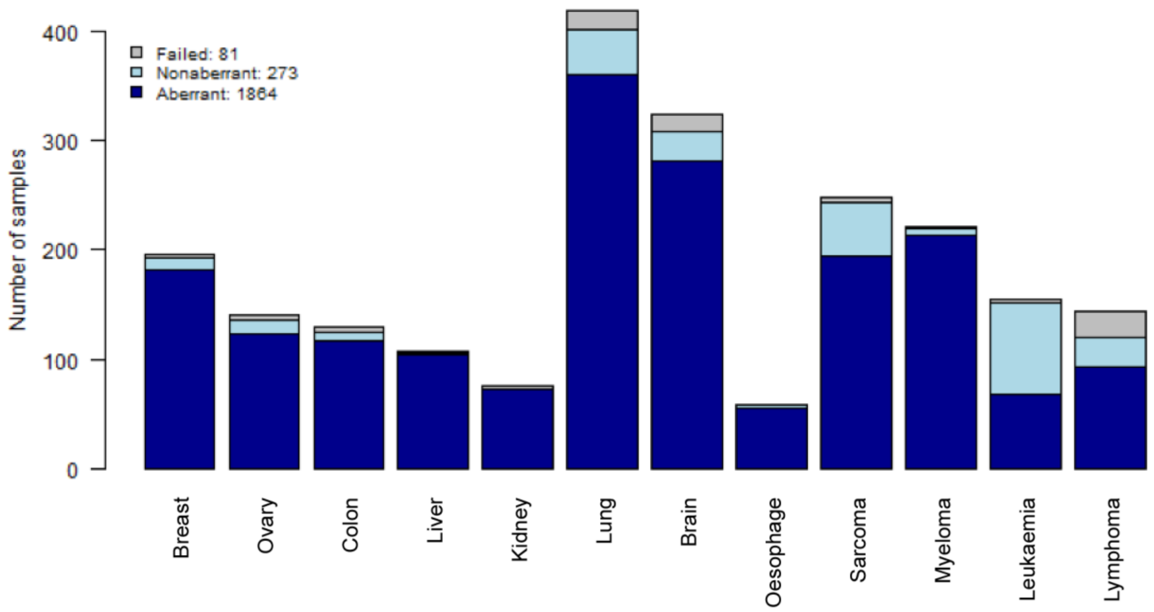

Supplementary Fig. 2: Tumour ploidy and homozygous deletion rate

Scatter plots of tumour ploidy versus (**a**) the number of homozygous deletions or (**b**) the length of homozygous deletions in the tumour, as inferred by ASCAT. Samples were considered tetraploid when they had a ploidy > 2.7 and diploid otherwise (dashed line in (**a**, **c**)). (**c**) Diploid tumours carry a higher number of homozygous deletions than tetraploid ones (Fisher-Pitman permutation test, p = 4.49 x 10^{-3}), while the size distribution (**d**) of the deletions is the same for both (Mann–Whitney *U* test, p = 0.675). (**e**) The frequencies of homozygous deletion at known tumour suppressor loci were not significantly different between diploid and tetraploid tumours, except for *RB1* (Fisher-Boschloo exact unconditional test, p = 1.01 x 10^{-3}).

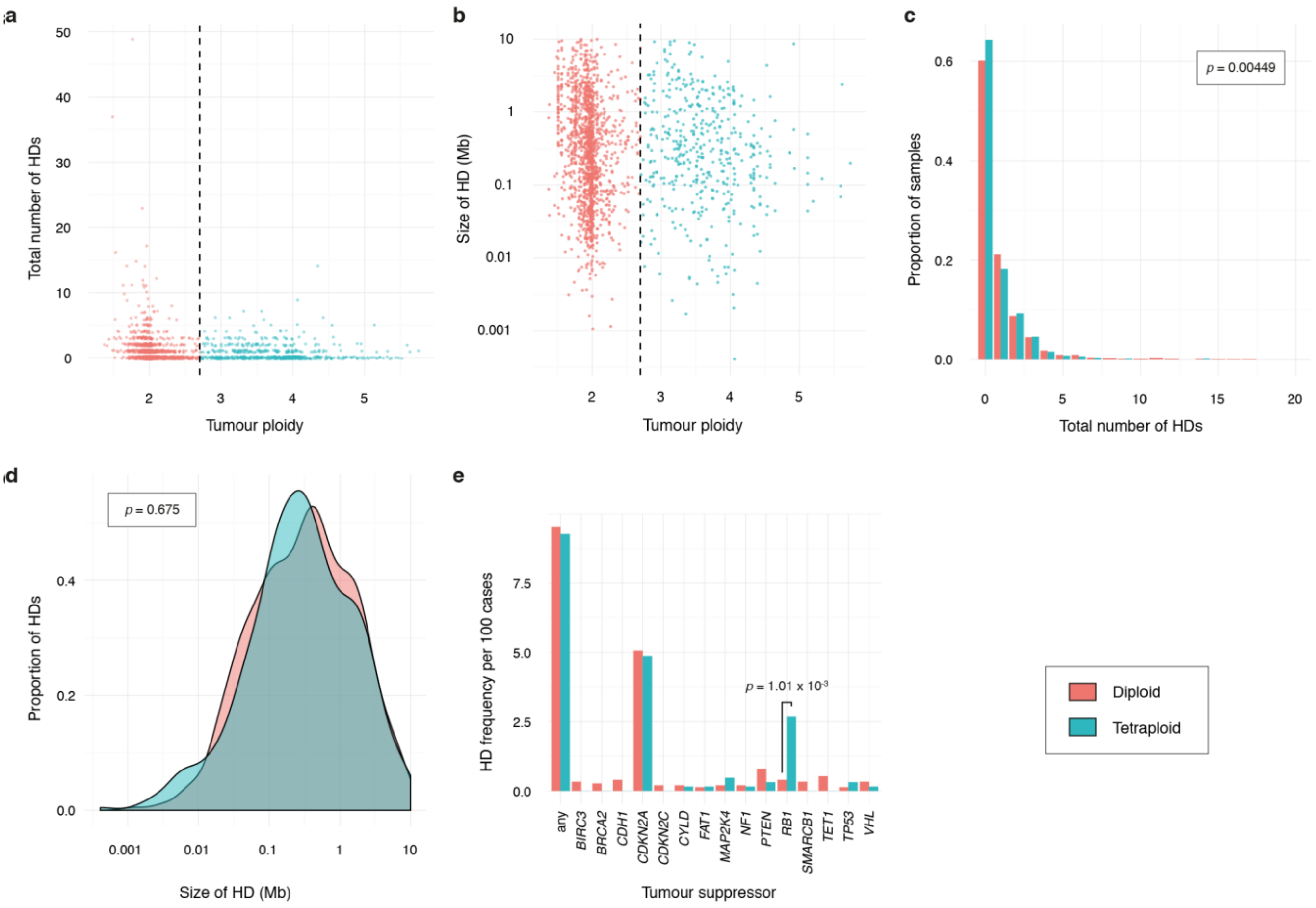

Supplementary Fig. 3: Homozygous deletions targeting known tumour suppressors

Positions of genes are indicated (black lines), as well as truncating mutations annotated in COSMIC, coloured according to tumour type and with symbols according to mutation type (nonsense, essential splice site, frame-shift insertion or deletion, in-frame insertion or deletion). When multiple somatic mutations in the same tumour type are annotated close together in COSMIC, their numbers are shown. Array probe positions are depicted below the genes. Homozygous deletions are shown as bold lines and small hemizygous deletions as dotted lines, both coloured according to tumour type. (**a**) *CDKN2C*, (**b**) *FANCD2* and *VHL*, two known tumour suppressors located close together, (**c**) *FAT1*, (**d**) *CDKN2A* (and *CDKN2B*), evidently the dominant homozygously deleted tumour suppressors, with homozygous deletions across 9 cancer types, (**e**) *TET1*, (**f**) *PTEN*, (**g**) *BIRC3* (and *BIRC2*), (**h**) *BRCA2*, (**i**) *RB1*, predominantly found homozygously deleted in sarcoma, (**j**) *CYLD*, homozygously deleted specifically in multiple myelomas, (**k**) *CDH1*, (**l**) *TP53*, (**m**) *MAP2K4*, (**n**) *NF1* and (**o**) *SMARCB1*, homozygously deleted specifically in brain tumours.

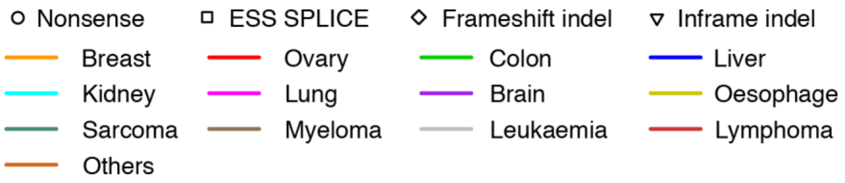

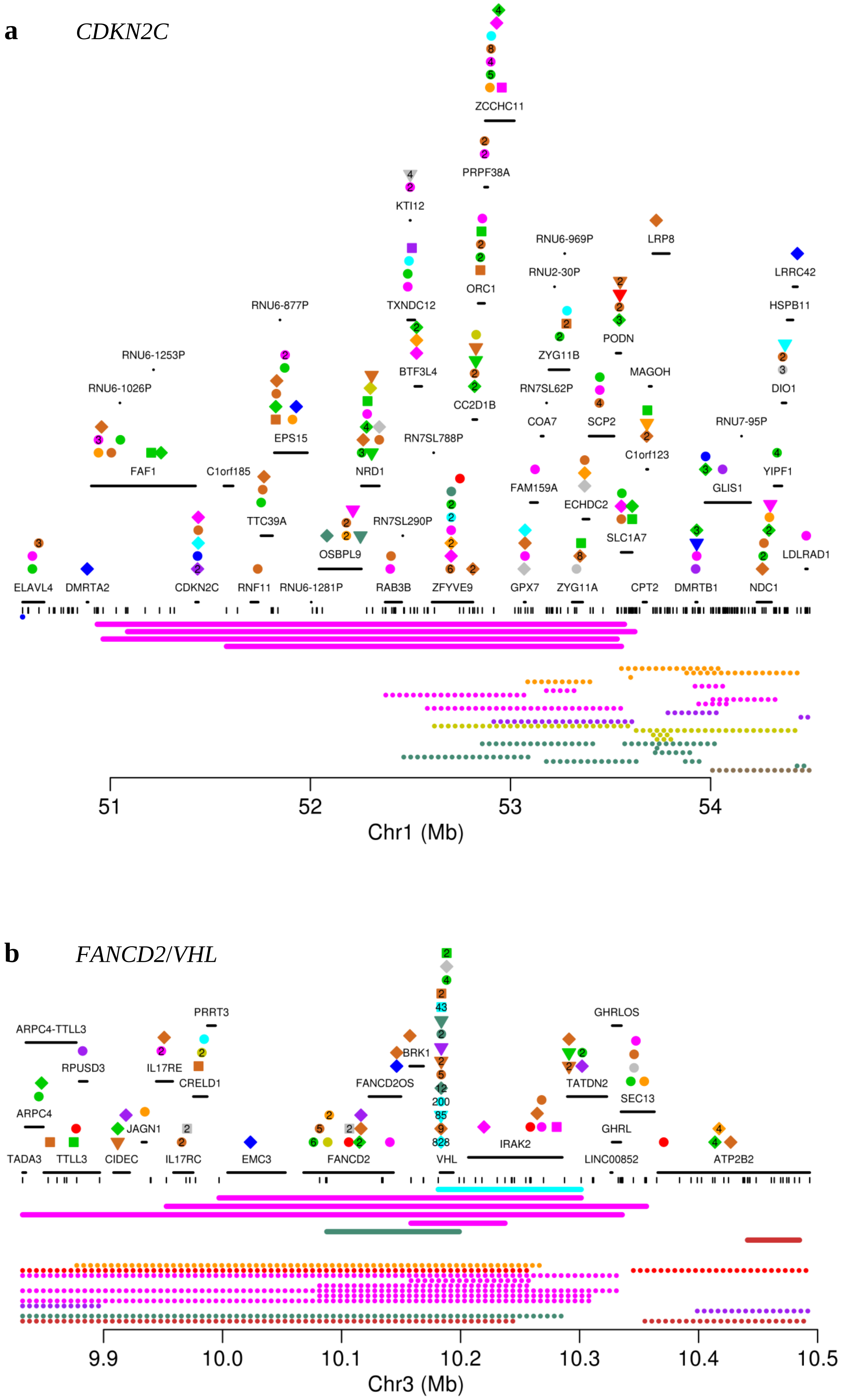
a
CDKN2C
ZCCHC11
PRPF38A
KTI12
TXNDC12
ORC1
RNU6-969P
LRP8
RNU2-30P
LRRC42
HSPB11
RNU6-877P
PODN
RNU6-1253P
ZYG11B
BTF3L4
MAGOH
RNU6-1026P
RN7SL62P
DIO1
CC2D1B
EPS15
COA7
SCP2
RNU7-95P
RN7SL788P
C1orf123
FAF1
C1orf185
NRD1
YIPF1
FAM159A
GLIS1
ECHDC2
TTC39A
RN7SL290P
SLC1A7
OSBPL9
LDLRAD1
ELAVL4
DMRTA2
CDKN2C
RNF11
RNU6-1281P
RAB3B
ZFYVE9
GPX7
ZYG11A
CPT2
DMRTB1
NDC1
51
52
53
54
Chr1 (Mb)
b
FANCD2/VHL
GHRLOS
PRRT3
ARPC4-TTLL3
RPUSD3
IL17RE
BRK1
CRELD1
FANCD2OS
TATDN2
ARPC4
SEC13
JAGN1
GHRL
IRAK2
TADA3
TTLL3
CIDEC
IL17RC
EMC3
FANCD2
VHL
LINC00852
ATP2B2
9.9
10.0
10.1
10.2
10.3
10.4
10.5
Chr3 (Mb)

c *FAT1*

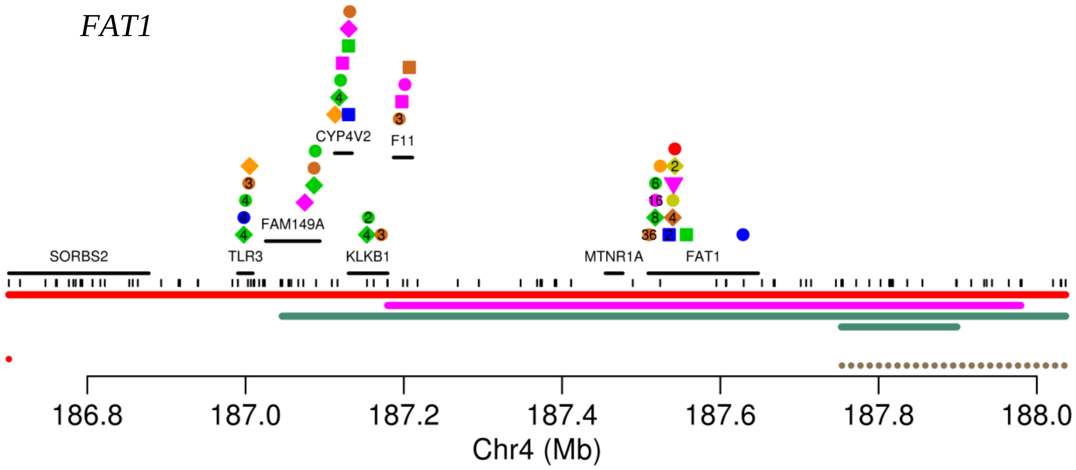

CYP4V2
F11
FAM149A
SORBS2
TLR3
KLKB1
MTNR1A
FAT1
186.8
187.0
187.2
187.4
187.6
187.8
188.0
Chr4 (Mb)

d *CDKN2A* (/*CDKN2B*)

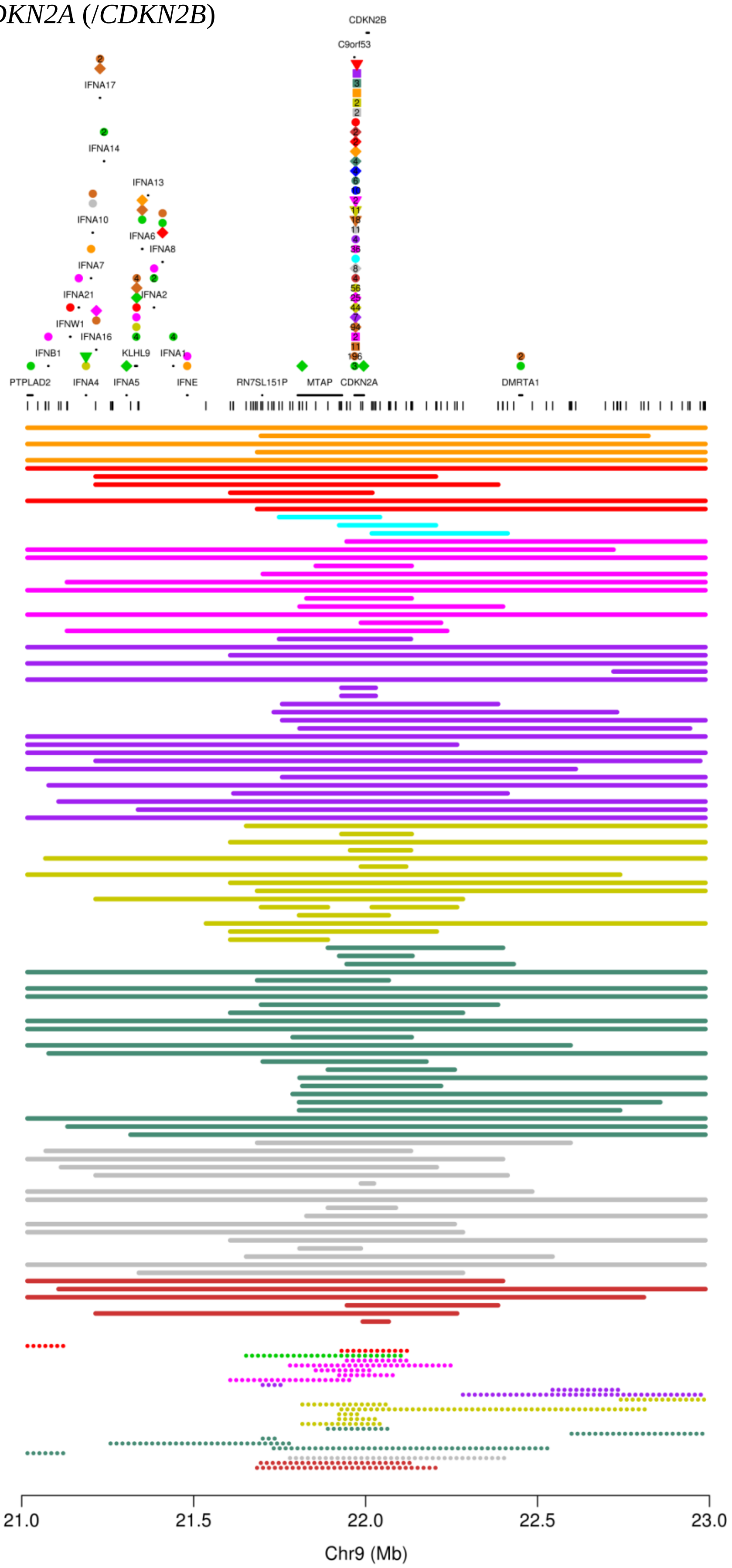

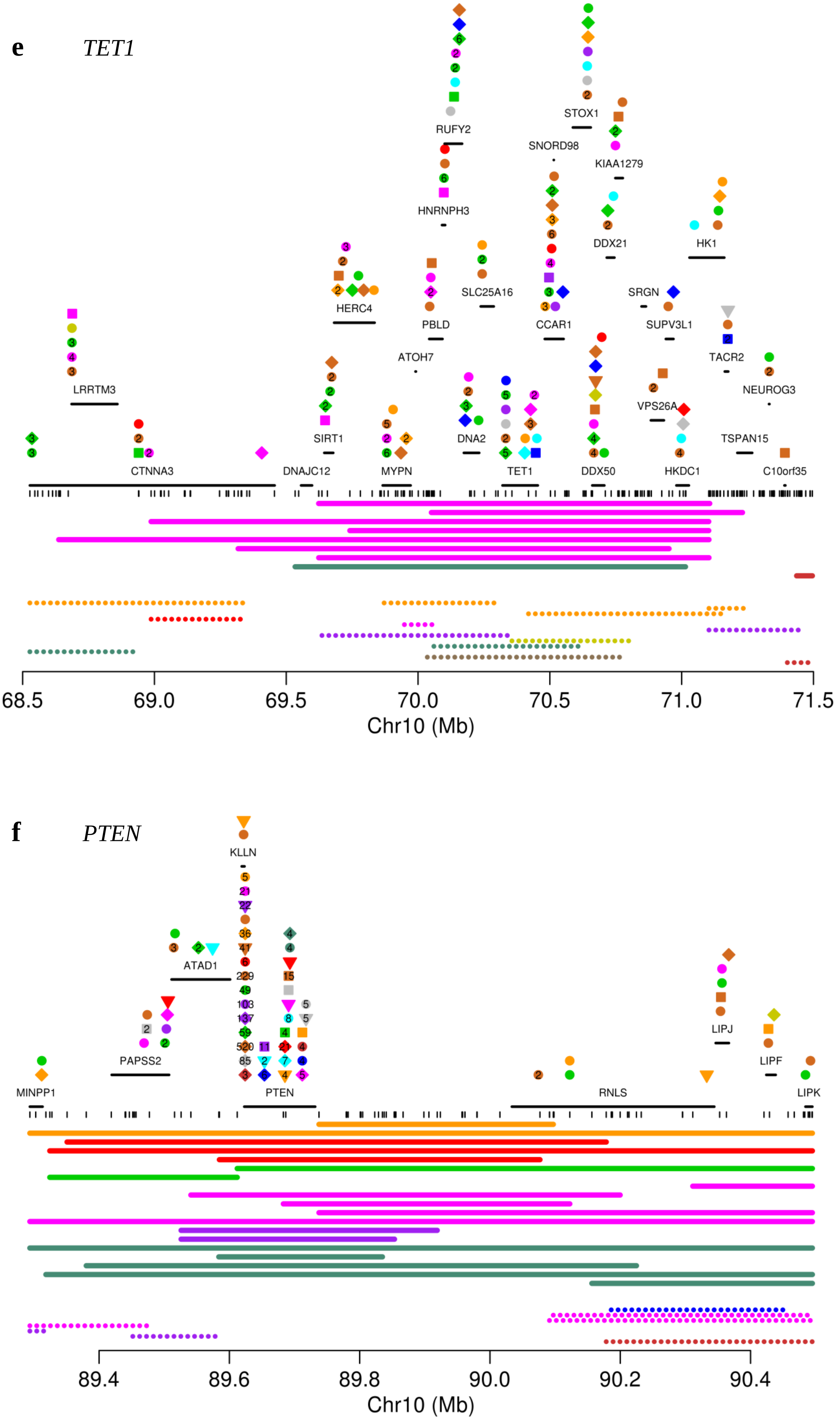
e
TET1
RUFY2
STOX1
SNORD98
KIAA1279
HNRNPH3
DDX21
HK1
HERC4
PBLD
SLC25A16
CCAR1
SRGN
SUPV3L1
TACR2
ATOH7
NEUROG3
LRRTM3
VPS26A
SIRT1
DNA2
MYPN
TET1
DDX50
HKDC1
TSPAN15
CTNNA3
DNAJC12
C10orf35
68.5
69.0
69.5
70.0
70.5
71.0
71.5
Chr10 (Mb)
f
PTEN
KLLN
ATAD1
PAPSS2
MINPP1
PTEN
RNLS
LIPJ
LIPF
LIPK
89.4
89.6
89.8
90.0
90.2
90.4
Chr10 (Mb)

g *BIRC2 (/BIRC3)*

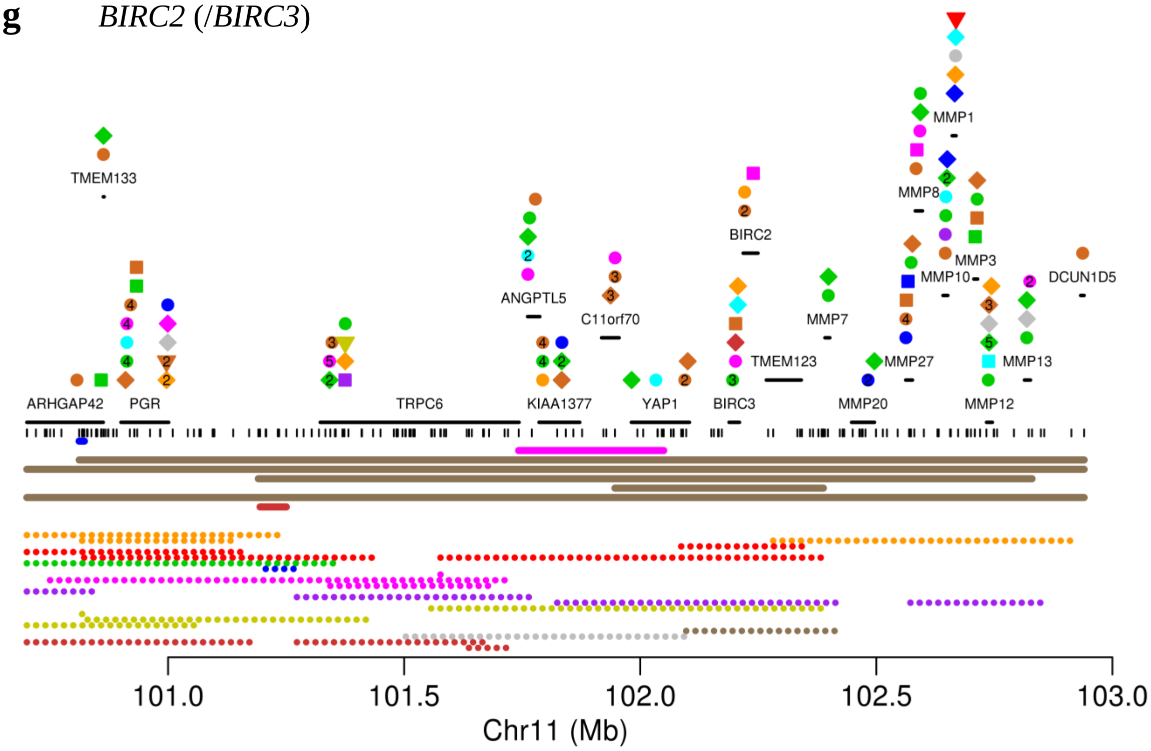

h *BRCA2*

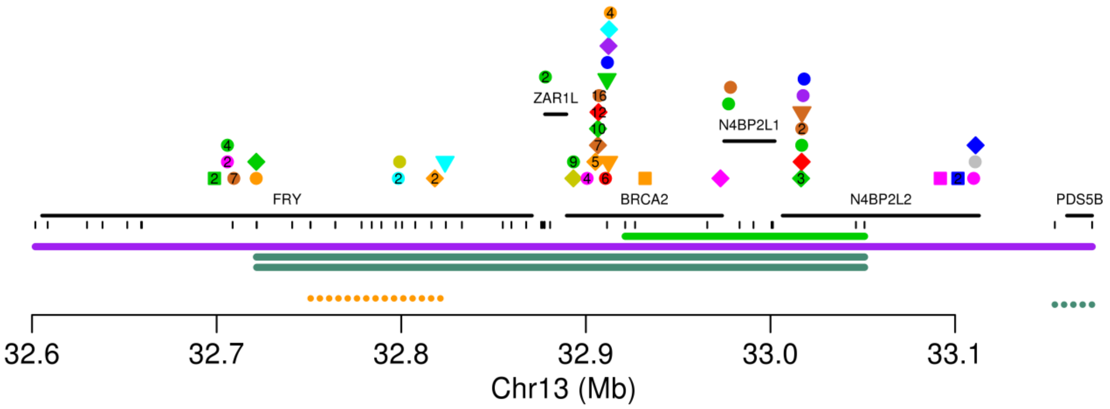

i *RB1*

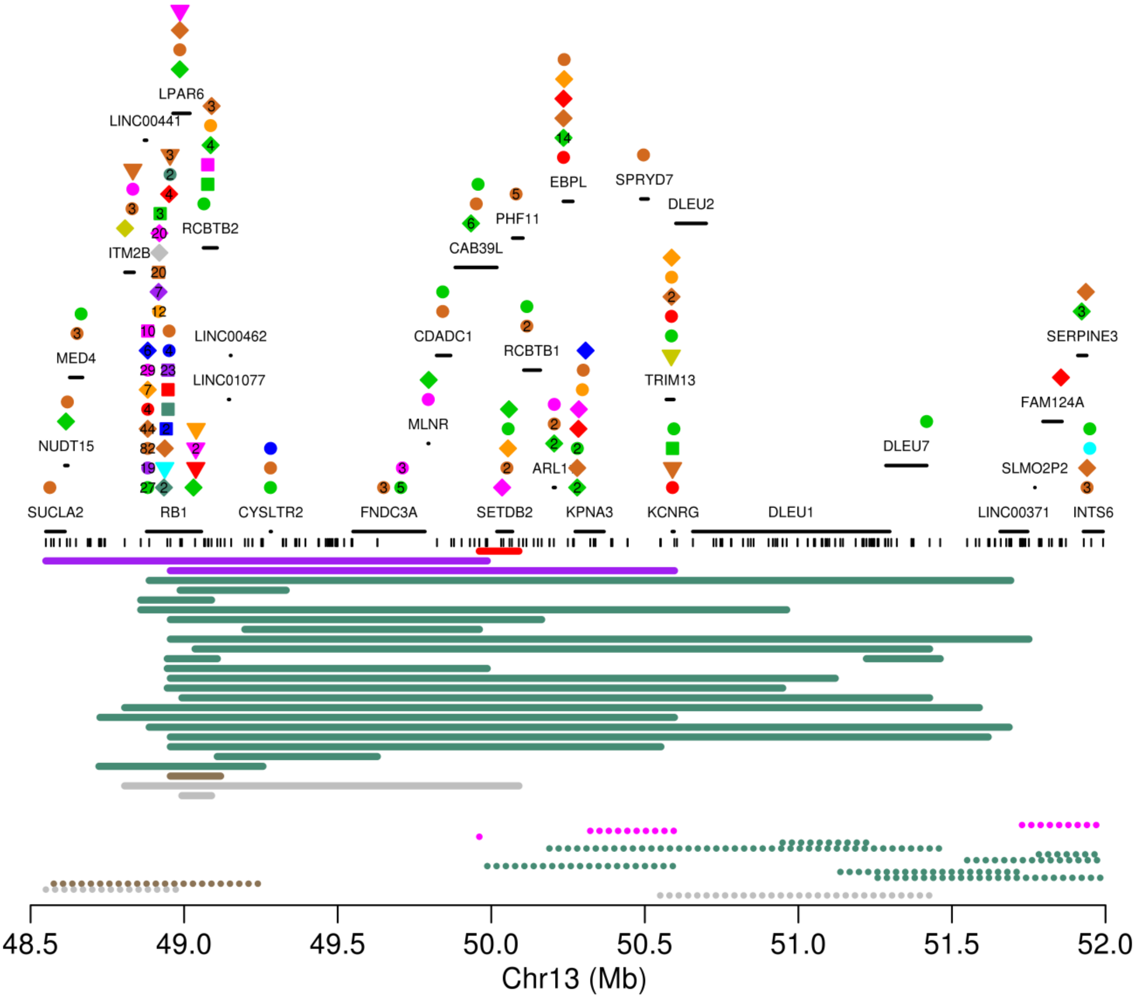
LPAR6
LINC00441
RCBTB2
ITM2B
MED4
NUDT15
SUCLA2
RB1
LINC00462
LINC01077
CYSLTR2
FNDC3A
MLNR
CDADC1
CAB39L
PHF11
SETDB2
RCBTB1
ARL1
KPNA3
EBPL
SPRYD7
DLEU2
TRIM13
KCNRG
DLEU1
DLEU7
SERPINE3
FAM124A
SLMO2P2
LINC00371
INTS6
48.5
49.0
49.5
50.0
50.5
51.0
51.5
52.0
Chr13 (Mb)

j *CYLD*

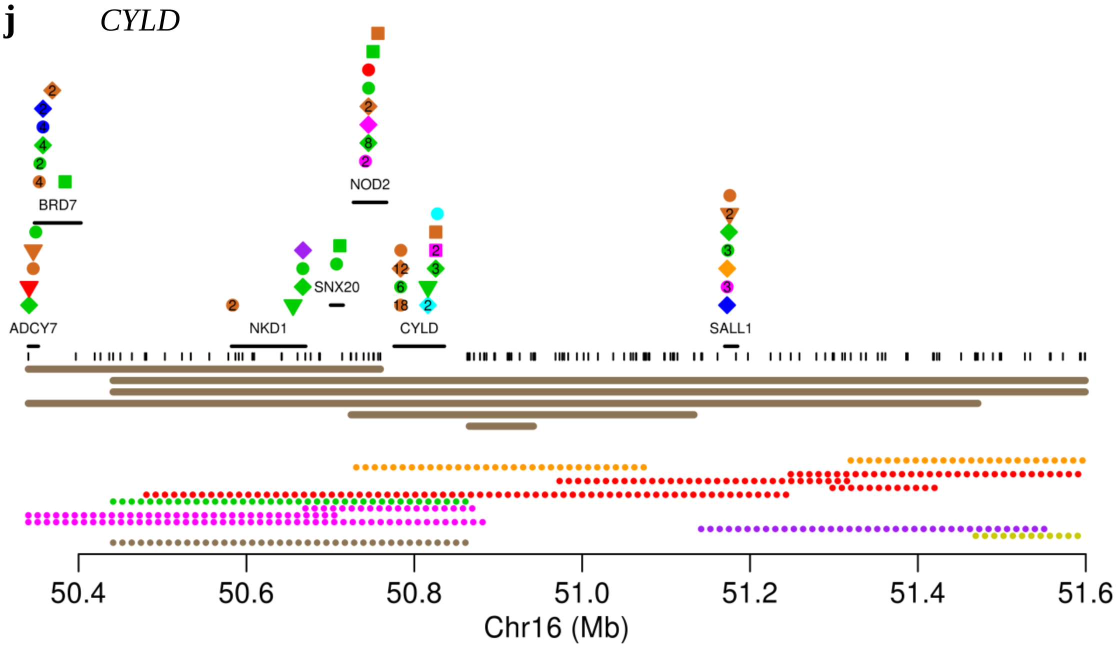
BRD7
ADCY7
NKD1
SNX20
NOD2
CYLD
SALL1
50.4
50.6
50.8
51.0
51.2
51.4
51.6
Chr16 (Mb)

k *CDH1*

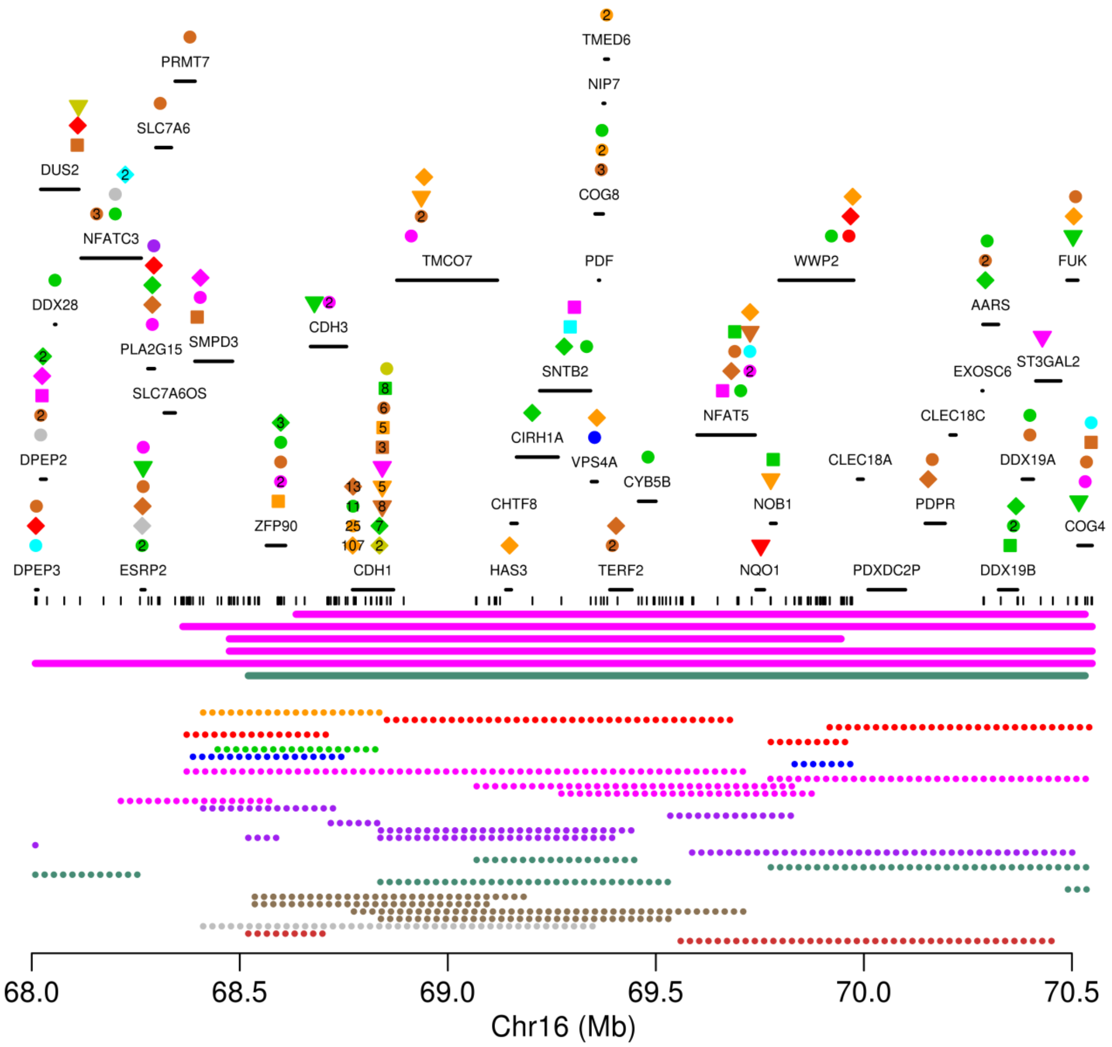
TMED6
PRMT7
NIP7
SLC7A6
DUS2
COG8
NFATC3
TMCO7
PDF
WWP2
FUK
DDX28
PLA2G15
SMPD3
CDH3
AARS
SLC7A6OS
SNTB2
EXOSC6
ST3GAL2
DPEP2
CIRH1A
VPS4A
NFAT5
CLEC18C
CYB5B
CLEC18A
DDX19A
ZFP90
CHTF8
NOB1
PDPR
COG4
DPEP3
ESRP2
CDH1
HAS3
TERF2
NQO1
PDXDC2P
DDX19B
68.0
68.5
69.0
69.5
70.0
70.5
Chr16 (Mb)

l *TP53*

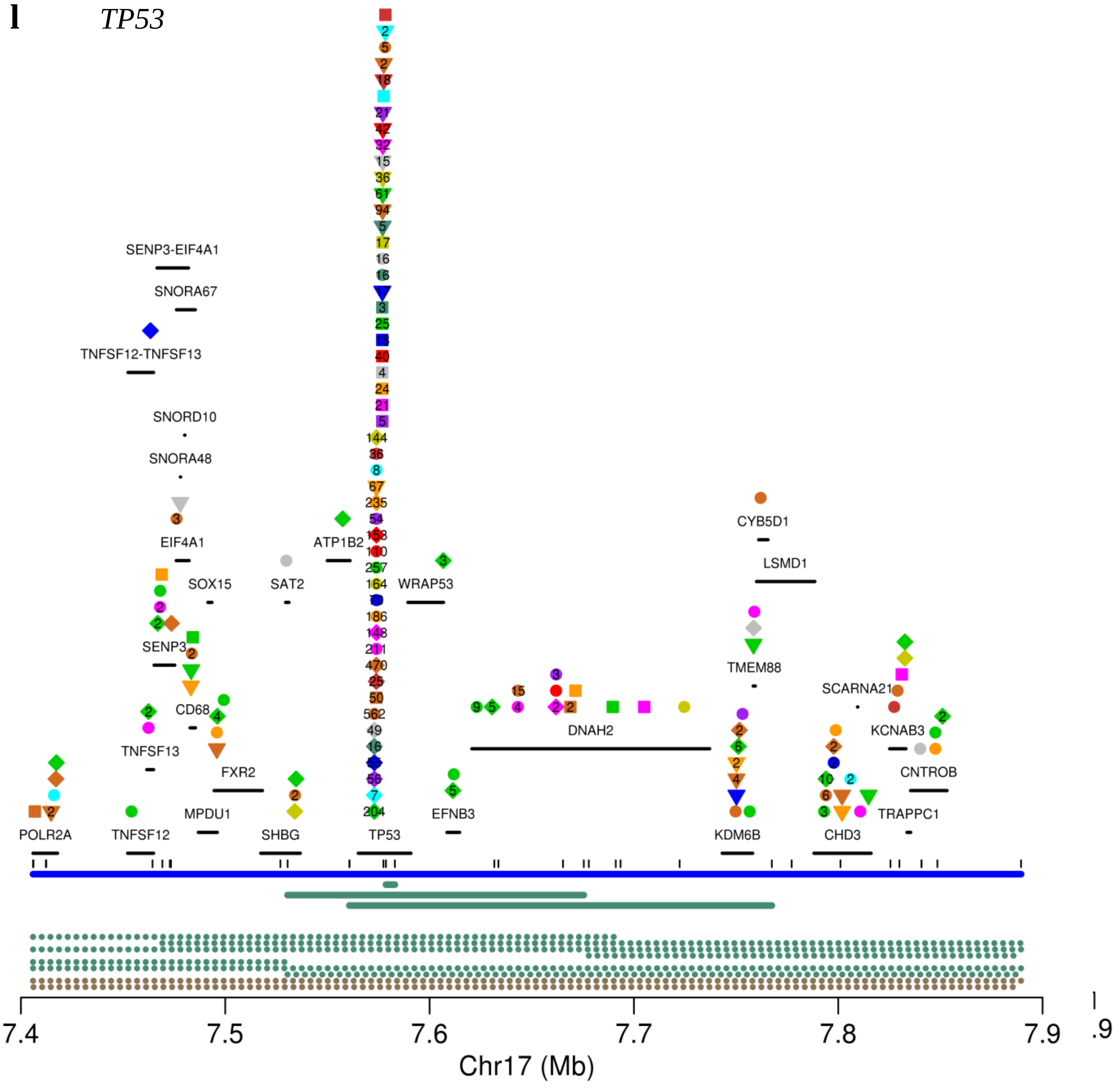

m *MAP2K4*

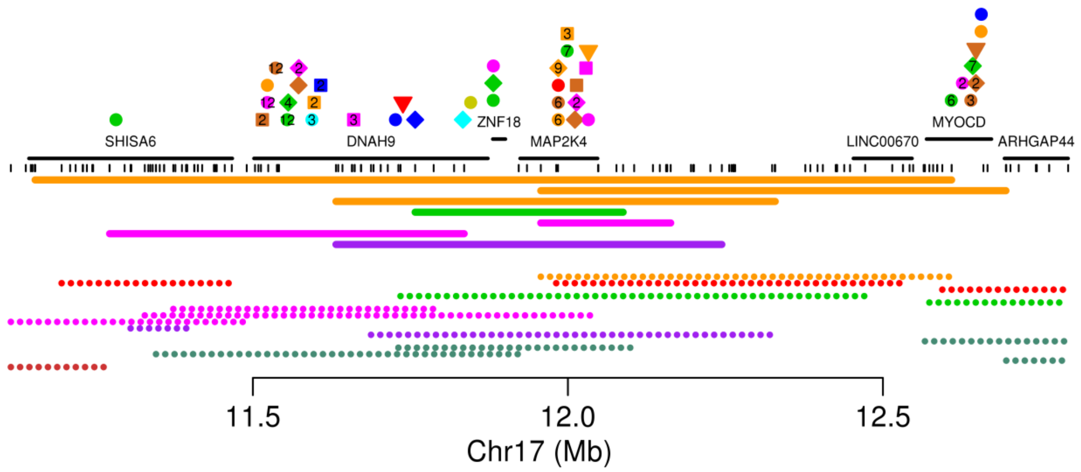

n *NF1*

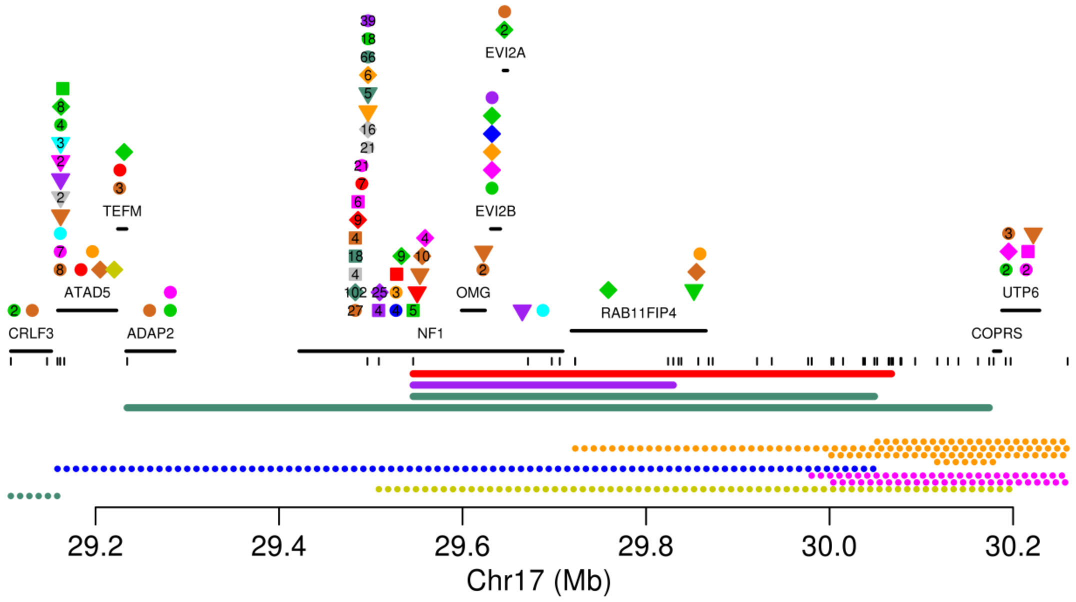

o *SMARCB1*

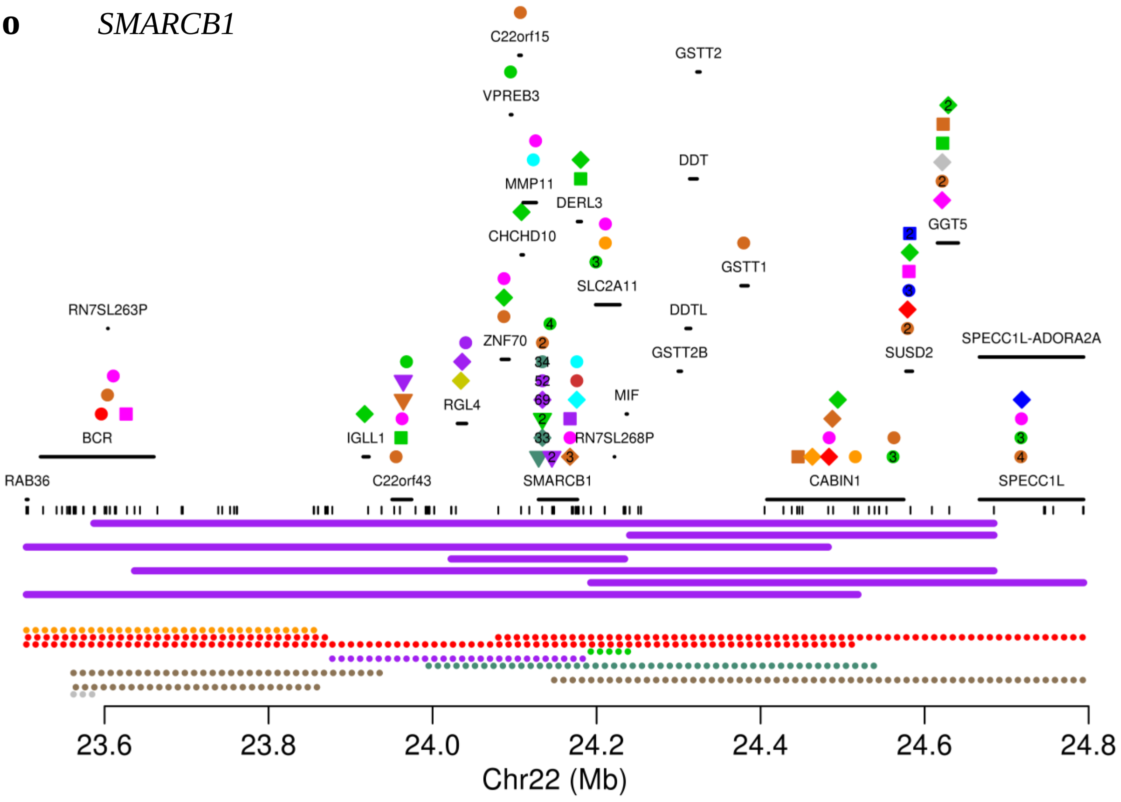

Supplementary Fig. 4: Homozygous deletions targeting T-cell receptor and immunoglobulin loci

Positions of genes are indicated (black lines), as well as truncating mutations annotated in COSMIC, coloured according to tumour type and with symbols according to mutation type (nonsense, essential splice site, frame-shift insertion or deletion, in-frame insertion or deletion). When multiple somatic mutations in the same tumour type are annotated close together in COSMIC, their numbers are shown. Array probe positions are depicted below the genes. Homozygous deletions are shown as bold lines and small hemizygous deletions as dotted lines, both coloured according to tumour type. (**a**) T cell receptor alpha locus, (**b**) immunoglobulin heavy chain locus and (**c**) immunoglobulin light chain locus. Homozygous deletions identified in these loci represent somatic homozygous losses in precursors of normal T and B lymphocytes that later developed into tumour cells. These homozygous deletions *per se* most likely do not play a role in oncogenesis.

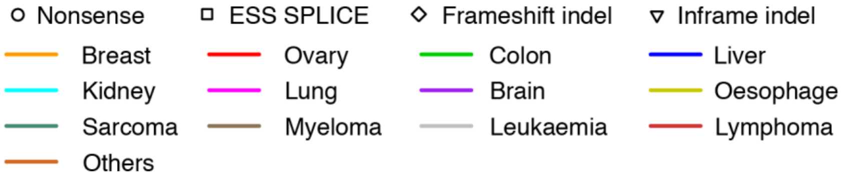

a *TCRA*

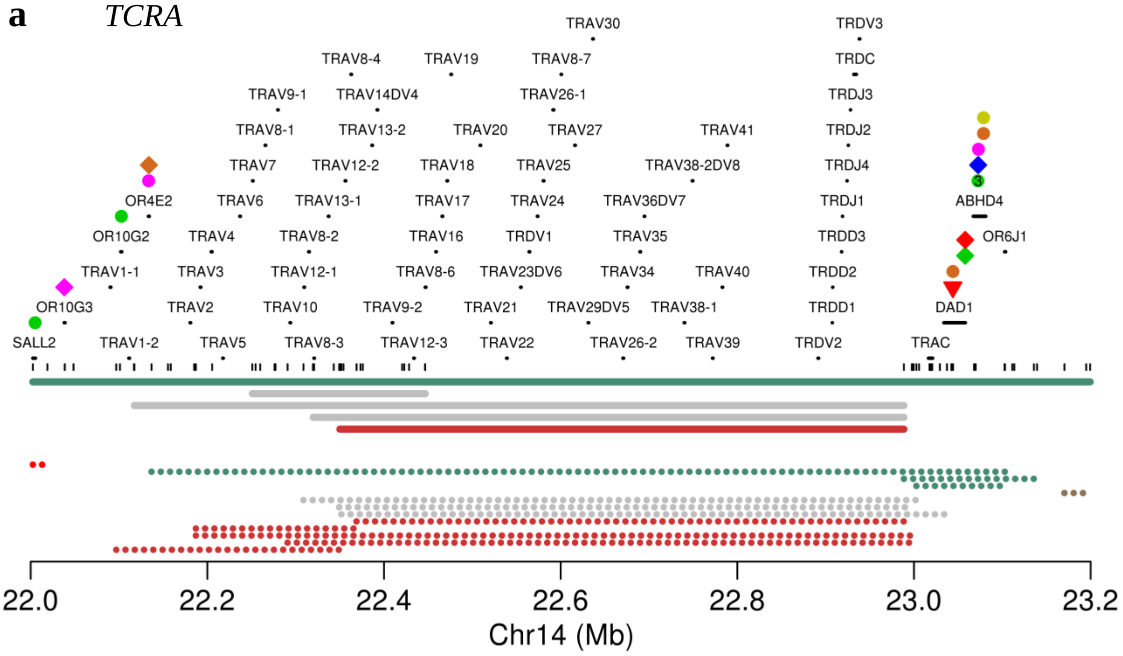
TRAV30
TRDV3
TRAV8-4
TRAV19
TRAV8-7
TRDC
TRAV9-1
TRAV14DV4
TRAV26-1
TRDJ3
TRAV8-1
TRAV13-2
TRAV20
TRAV27
TRAV41
TRDJ2
TRAV7
TRAV12-2
TRAV18
TRAV25
TRAV38-2DV8
TRDJ4
OR4E2
TRAV6
TRAV13-1
TRAV17
TRAV24
TRAV36DV7
TRDJ1
ABHD4
OR10G2
TRAV4
TRAV8-2
TRAV16
TRDV1
TRAV35
TRDD3
OR6J1
TRAV1-1
TRAV3
TRAV12-1
TRAV8-6
TRAV23DV6
TRAV34
TRAV40
TRDD2
OR10G3
TRAV2
TRAV10
TRAV9-2
TRAV21
TRAV29DV5
TRAV38-1
TRDD1
DAD1
SALL2
TRAV1-2
TRAV5
TRAV8-3
TRAV12-3
TRAV22
TRAV26-2
TRAV39
TRDV2
TRAC
22.0
22.2
22.4
22.6
22.8
23.0
23.2
Chr14 (Mb)

b *IGH*

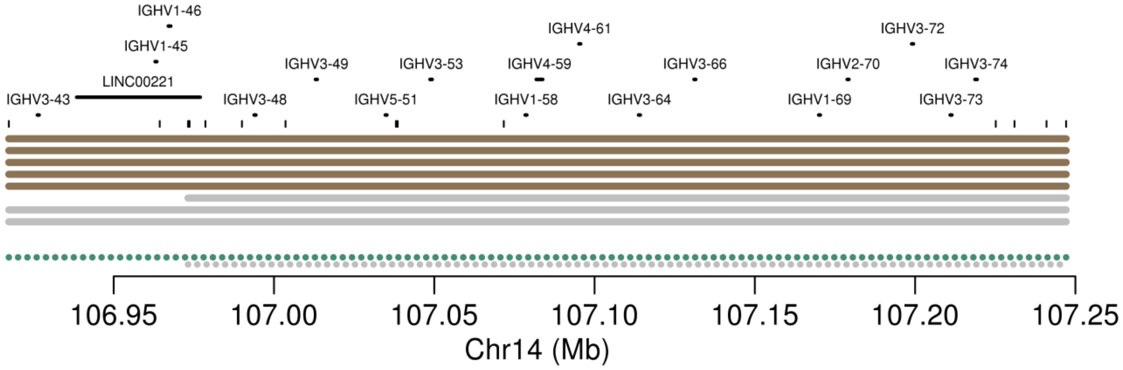
IGHV1-46
IGHV1-45
IGHV4-61
IGHV3-72
IGHV3-49
IGHV3-53
IGHV4-59
IGHV3-66
IGHV2-70
IGHV3-74
LINC00221
IGHV3-43
IGHV3-48
IGHV5-51
IGHV1-58
IGHV3-64
IGHV1-69
IGHV3-73
106.95
107.00
107.05
107.10
107.15
107.20
107.25
Chr14 (Mb)

c *IGV*

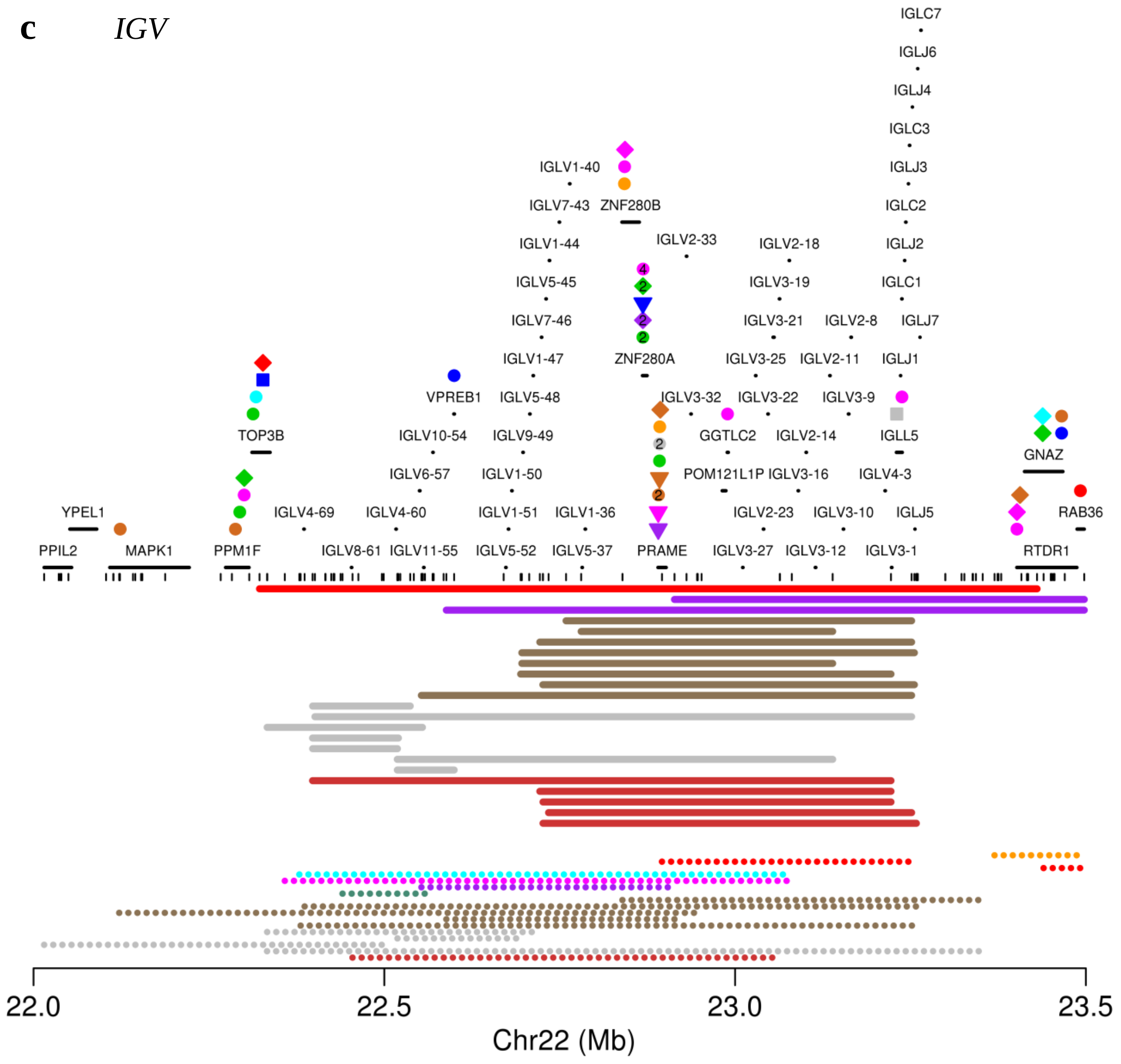
IGLC7
IGLJ6
IGLJ4
IGLC3
IGLJ3
IGLC2
IGLJ2
IGLC1
IGLJ7
IGLJ1
IGLL5
IGLJ5
IGLV1-40
IGLV7-43
ZNF280B
IGLV1-44
IGLV2-33
IGLV2-18
IGLV5-45
IGLV3-19
IGLV7-46
IGLV3-21
IGLV2-8
IGLV1-47
ZNF280A
IGLV3-25
IGLV2-11
VPREB1
IGLV5-48
IGLV3-32
IGLV3-22
IGLV3-9
TOP3B
IGLV10-54
IGLV9-49
GGTLC2
IGLV2-14
GNAZ
IGLV6-57
IGLV1-50
POM121L1P
IGLV3-16
IGLV4-3
YPEL1
IGLV4-69
IGLV4-60
IGLV1-51
IGLV1-36
IGLV2-23
IGLV3-10
RAB36
PPIL2
MAPK1
PPM1F
IGLV8-61
IGLV11-55
IGLV5-52
IGLV5-37
PRAME
IGLV3-27
IGLV3-12
IGLV3-1
RTDR1
22.0
22.5
23.0
23.5
Chr22 (Mb)

Supplementary Fig. 5: Homozygous deletions targeting 15 known fragile sites

Positions of genes are indicated (black lines), as well as truncating mutations annotated in COSMIC, coloured according to tumour type and with symbols according to mutation type (nonsense, essential splice site, frame-shift insertion or deletion, in-frame insertion or deletion). When multiple somatic mutations in the same tumour type are annotated close together in COSMIC, their numbers are shown. Array probe positions are depicted below the genes. Homozygous deletions are shown as bold lines and small hemizygous deletions as dotted lines, both coloured according to tumour type. (**a**) FRA1H, (**b**) FRA2F, (**c**) FRA3B, (**d**) FRA6A, (**e**) FRA6H, (**f**) FRA6D, (**g**) FRA6F, (**h**) FRA10F, (**i**) FRA12C, (**j**) FRA16B, (**k**) FRA16D, (**l**) FRA17A, (**m**) FRA19A, (**n**) FRAXB, (**o**) FRAXC. FRA16B contains known tumour suppressor *CDH1* (**Supplementary Fig. 2h**), specifically homozygously deleted in lung cancer, and FRA17A contains known tumour suppressor *MAP2K4* (**Supplementary Fig. 2j**), most often homozygously deleted in breast cancer.

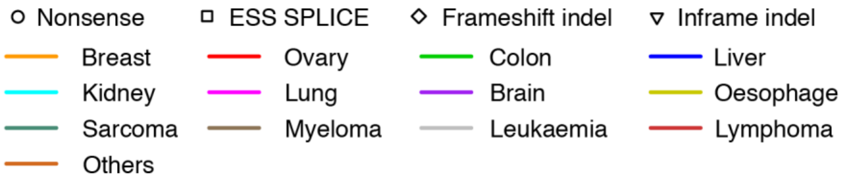

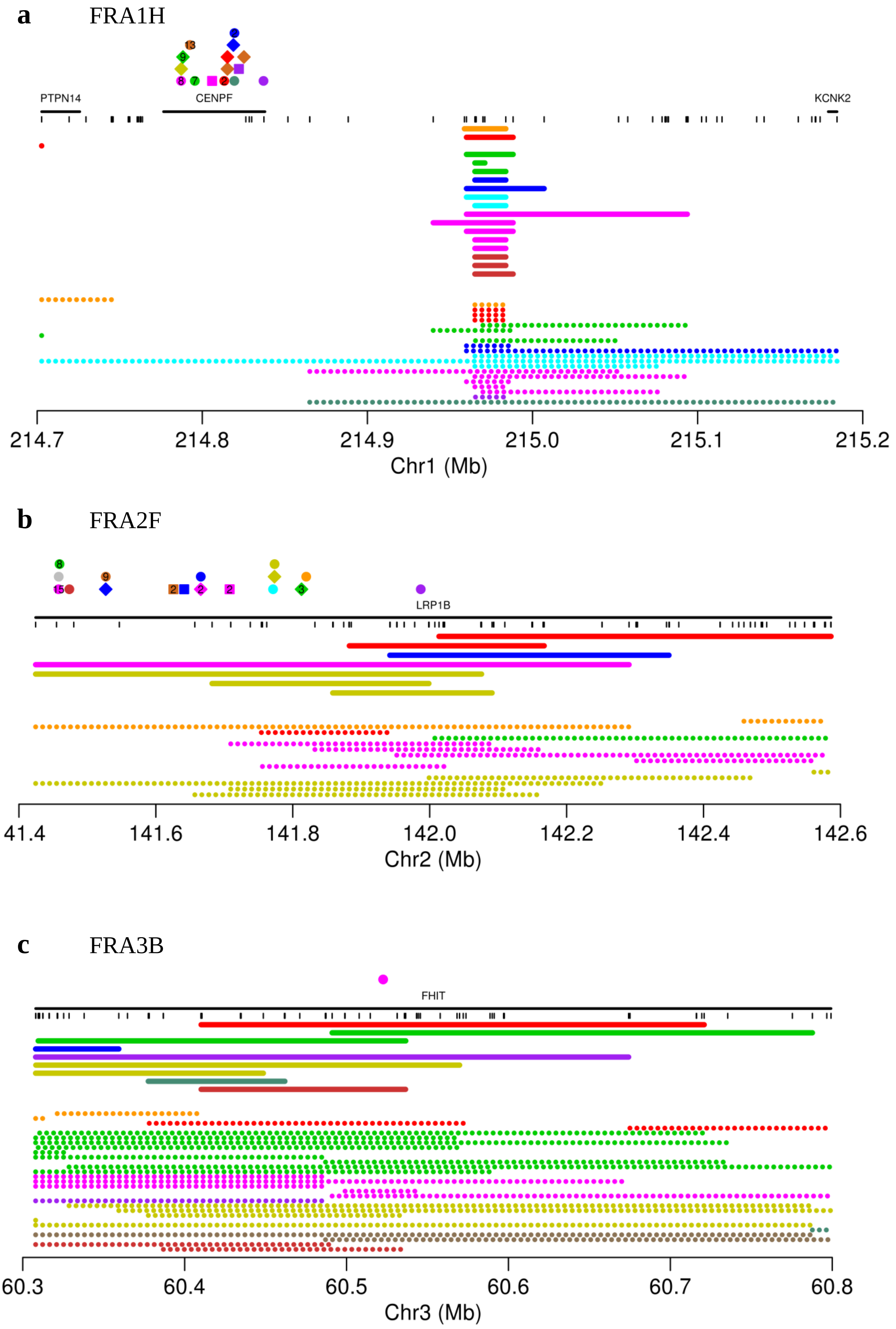
a
FRA1H
PTPN14
CENPF
KCNK2
214.7
214.8
214.9
215.0
215.1
215.2
Chr1 (Mb)
b
FRA2F
LRP1B
141.4
141.6
141.8
142.0
142.2
142.4
142.6
Chr2 (Mb)
c
FRA3B
FHIT
60.3
60.4
60.5
60.6
60.7
60.8
Chr3 (Mb)

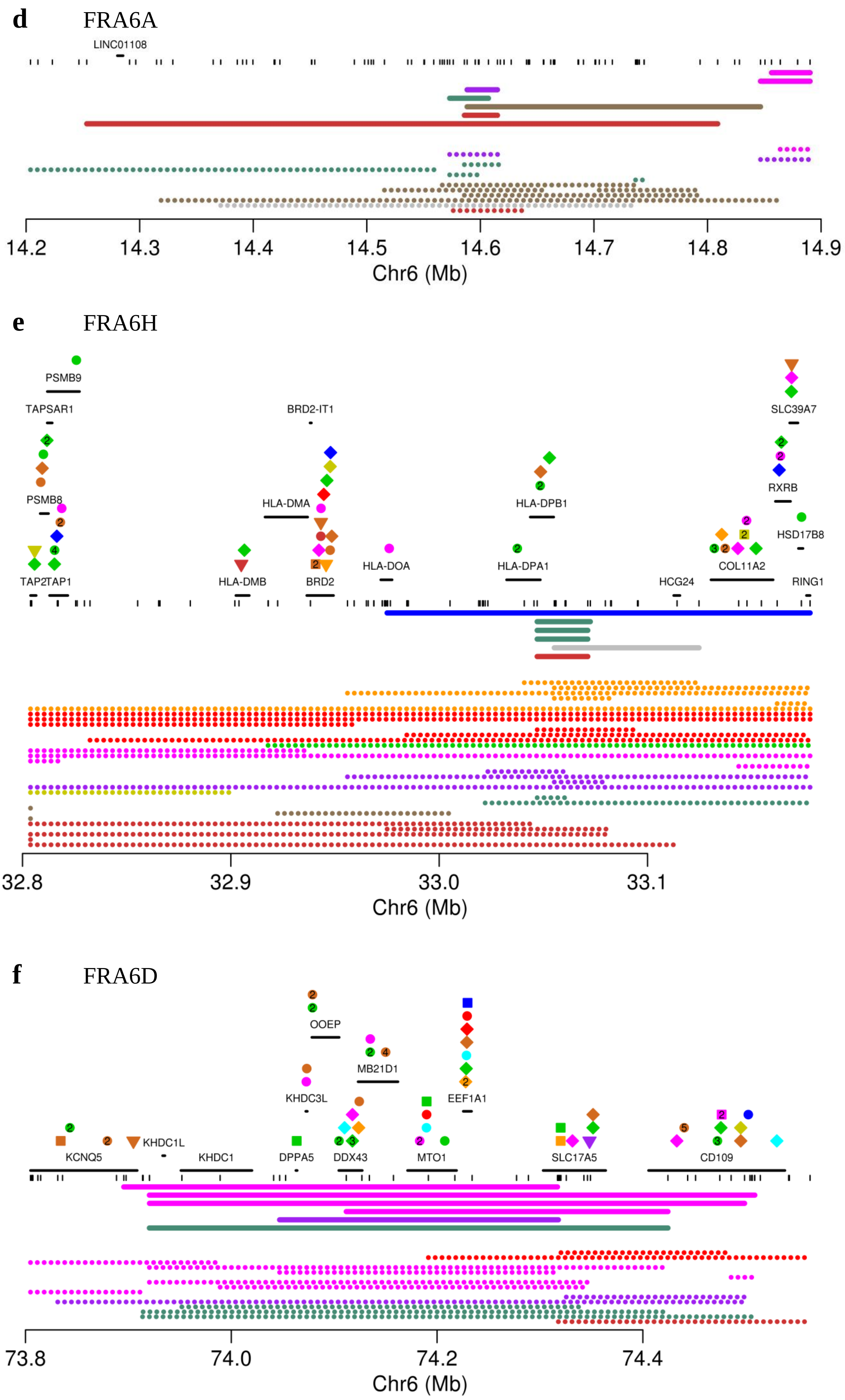
d
FRA6A
LINC01108
14.2
14.3
14.4
14.5
14.6
14.7
14.8
14.9
Chr6 (Mb)
e
FRA6H
PSMB9
TAPSAR1
BRD2-IT1
SLC39A7
PSMB8
HLA-DMA
HLA-DPB1
RXRB
HSD17B8
TAP2
TAP1
HLA-DMB
BRD2
HLA-DOA
HLA-DPA1
HCG24
COL11A2
RING1
32.8
32.9
33.0
33.1
Chr6 (Mb)
f
FRA6D
OOEP
MB21D1
KHDC3L
EEF1A1
KHDC1L
KCNQ5
KHDC1
DPPA5
DDX43
MTO1
SLC17A5
CD109
73.8
74.0
74.2
74.4
Chr6 (Mb)

g FRA6F

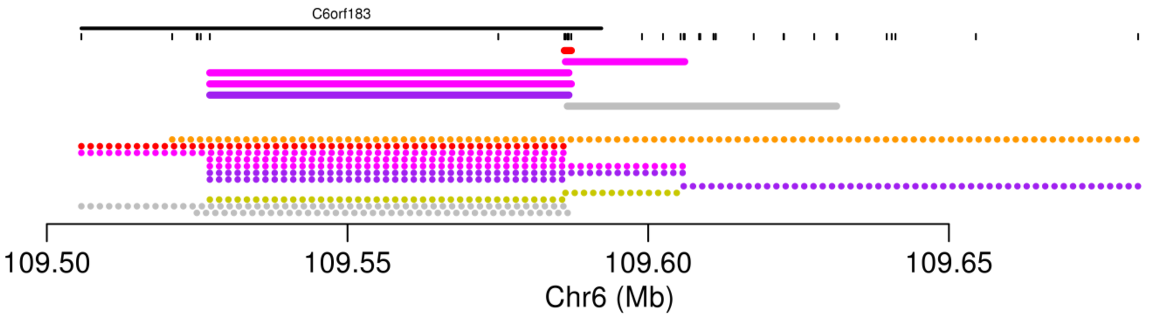

h FRA10F

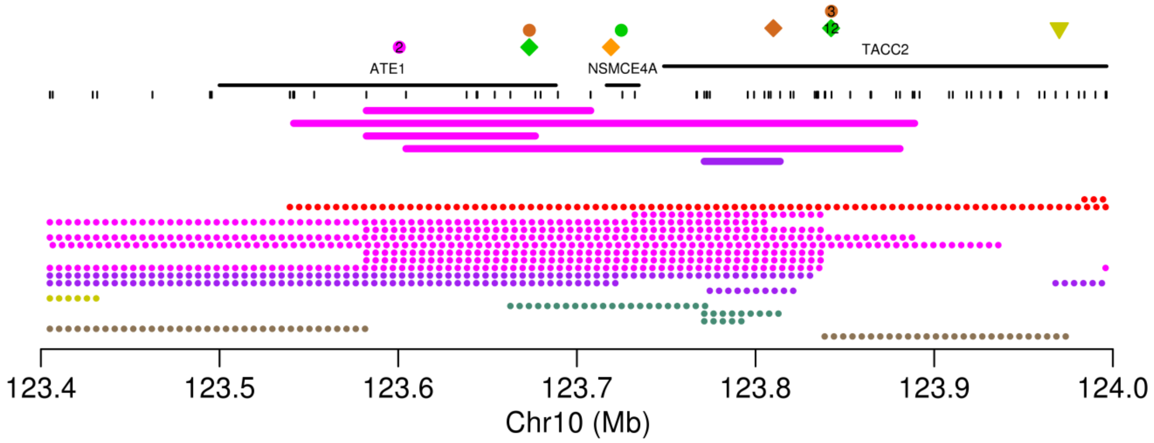

i FRA12C

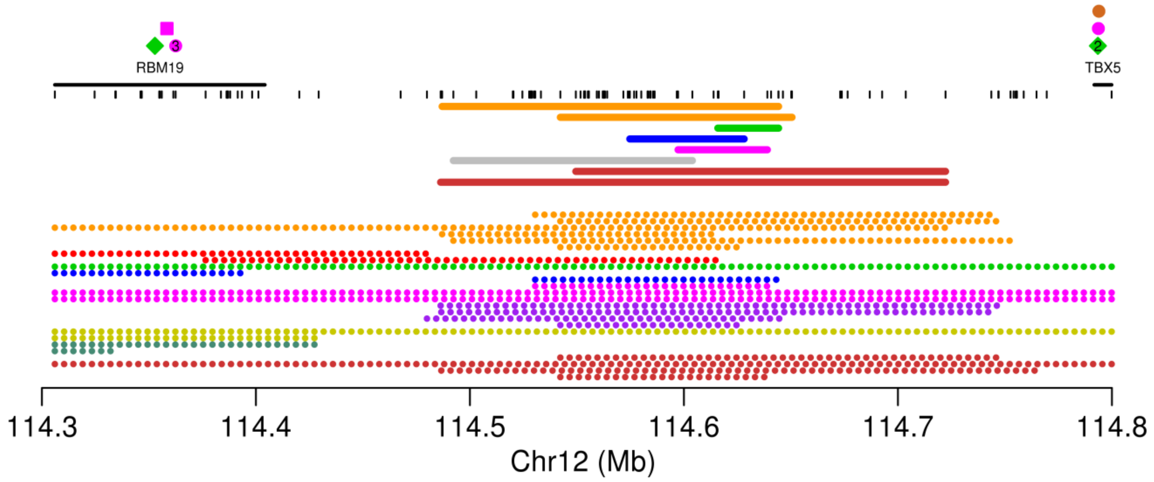

j FRA16B

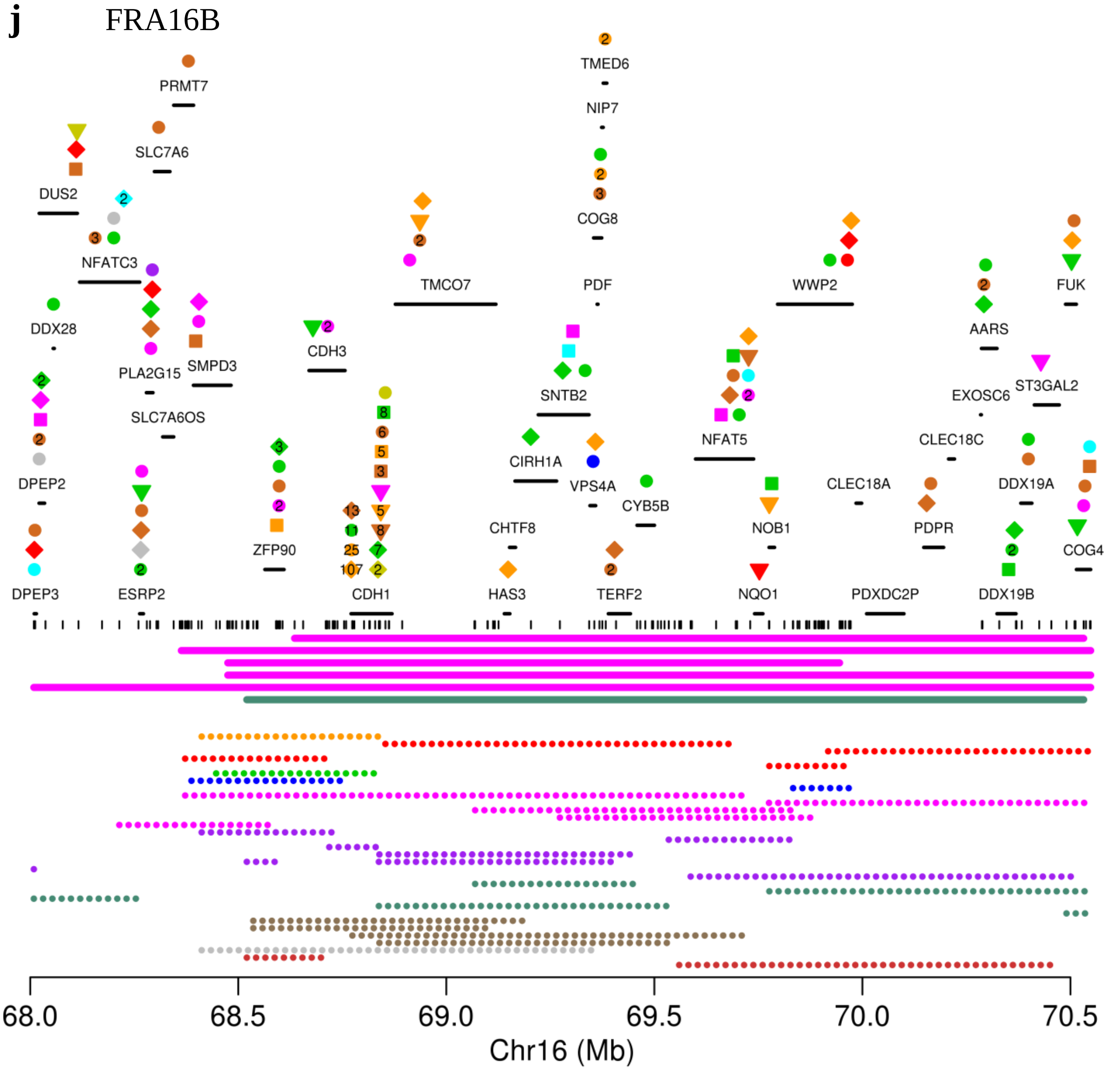

k FRA16D

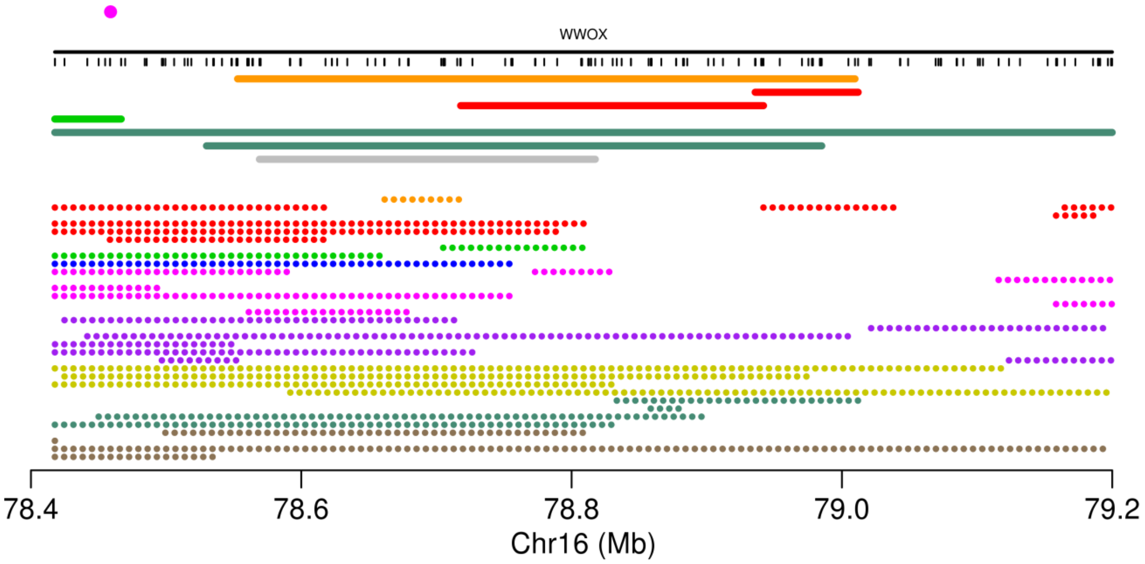

l FRA17A

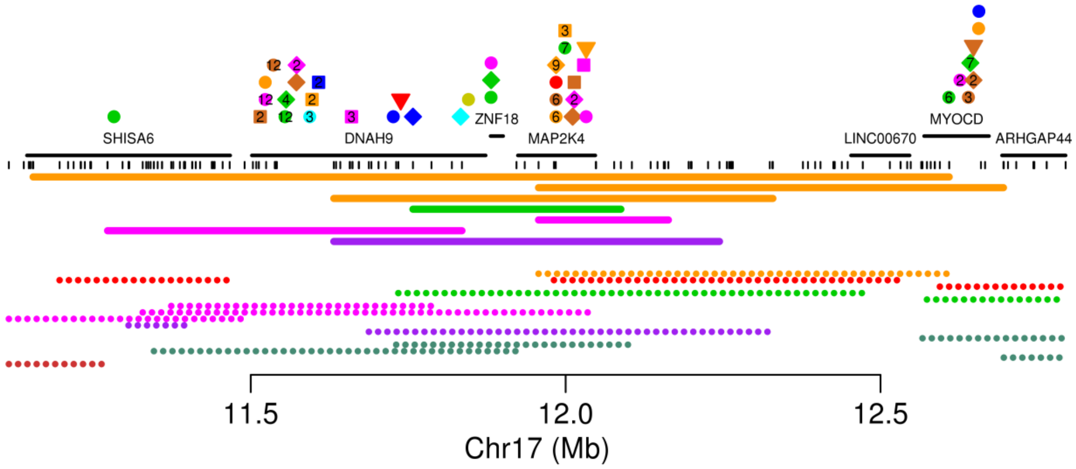

m FRA19A

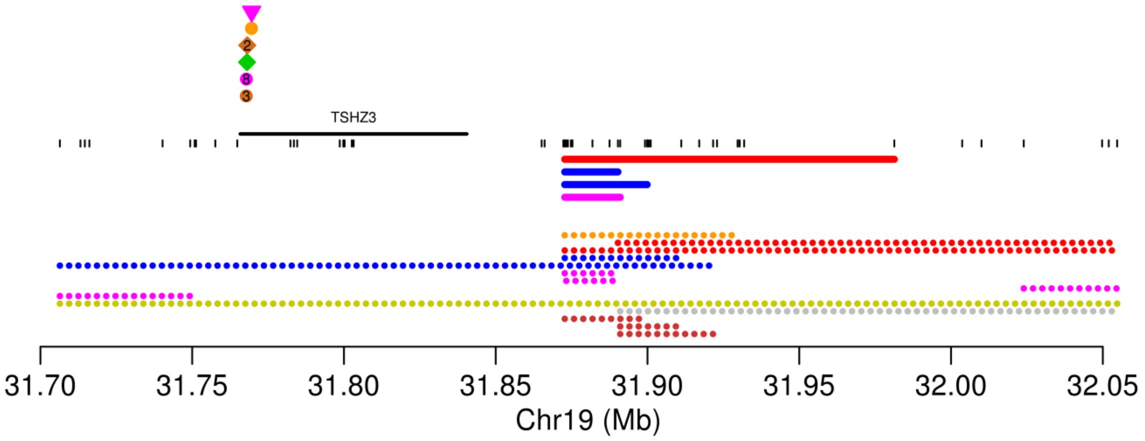

n FRAXB

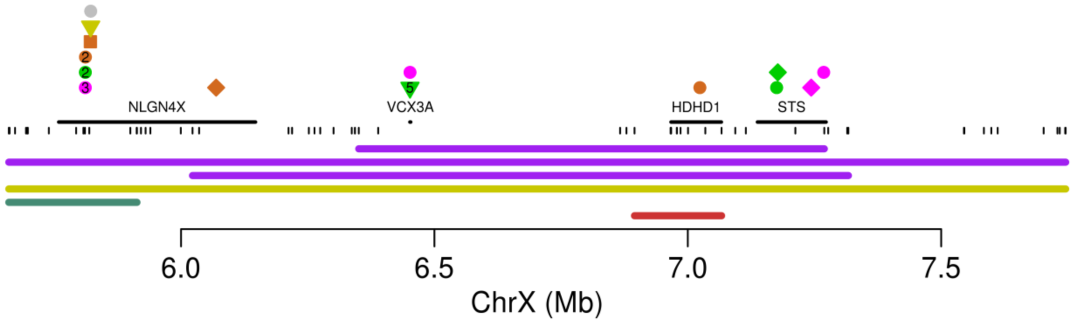

o FRAXC

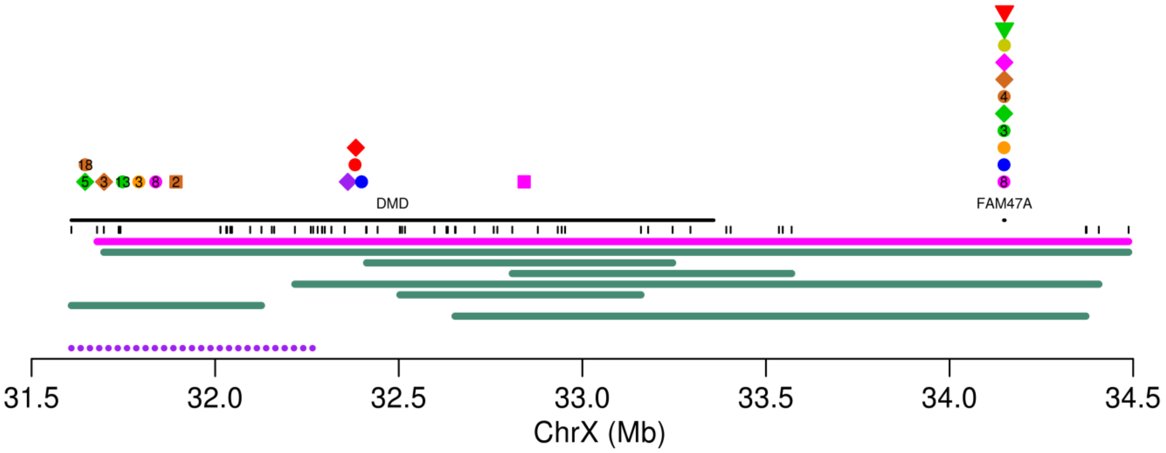

Supplementary Fig. 6: Homozygous deletions targeting 24 predicted fragile sites

Positions of genes are indicated (black lines), as well as truncating mutations annotated in COSMIC, coloured according to tumour type and with symbols according to mutation type (nonsense, essential splice site, frame-shift insertion or deletion, in-frame insertion or deletion). When multiple somatic mutations in the same tumour type are annotated close together in COSMIC, their numbers are shown. Array probe positions are depicted below the genes. Homozygous deletions are shown as bold lines and small hemizygous deletions as dotted lines, both coloured according to tumour type. (**a**) chr2:61.83-61.96, (**b**) chr3:73.00-73.04, (**c**) chr3:116.69-116.83, (**d**) chr4:78.17-78.21, (**e**) chr6:18.17-18.21, (**f**) chr6:40.56-40.61, (**g**) chr8:3.32-3.39, (**h**) chr8:6.38-6.43, (**i**) chr8:98.84-98.85, (**j**) chr9:121.64-121.65, (**k**) chr9:133.39-133.62, (**l**) chr10:12.21-12.46, (**m**) chr13:28.25-28.26, (**n**) chr14:26.40-28.04, (**o**) chr14:96.22-96.22, (**p**) chr15:50.66-51.11, (**q**) chr16:6.74-6.76, (**r**) chr16:9.40-9.40, (**s**) chr16:10.07-10.08, (**t**) chr16:70.81-71.58, (**u**) chr19:9.06-9.07, (**v**) chr20:15.08-15.11, (**w**) chrX:3.22-4.12, (**x**) chrX:9.10-10.97.

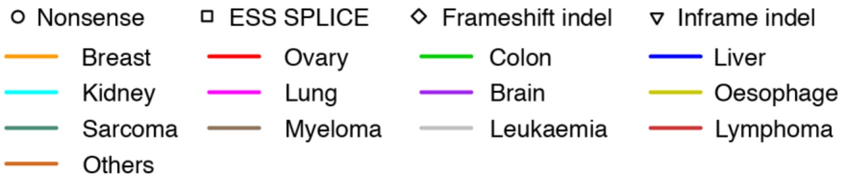

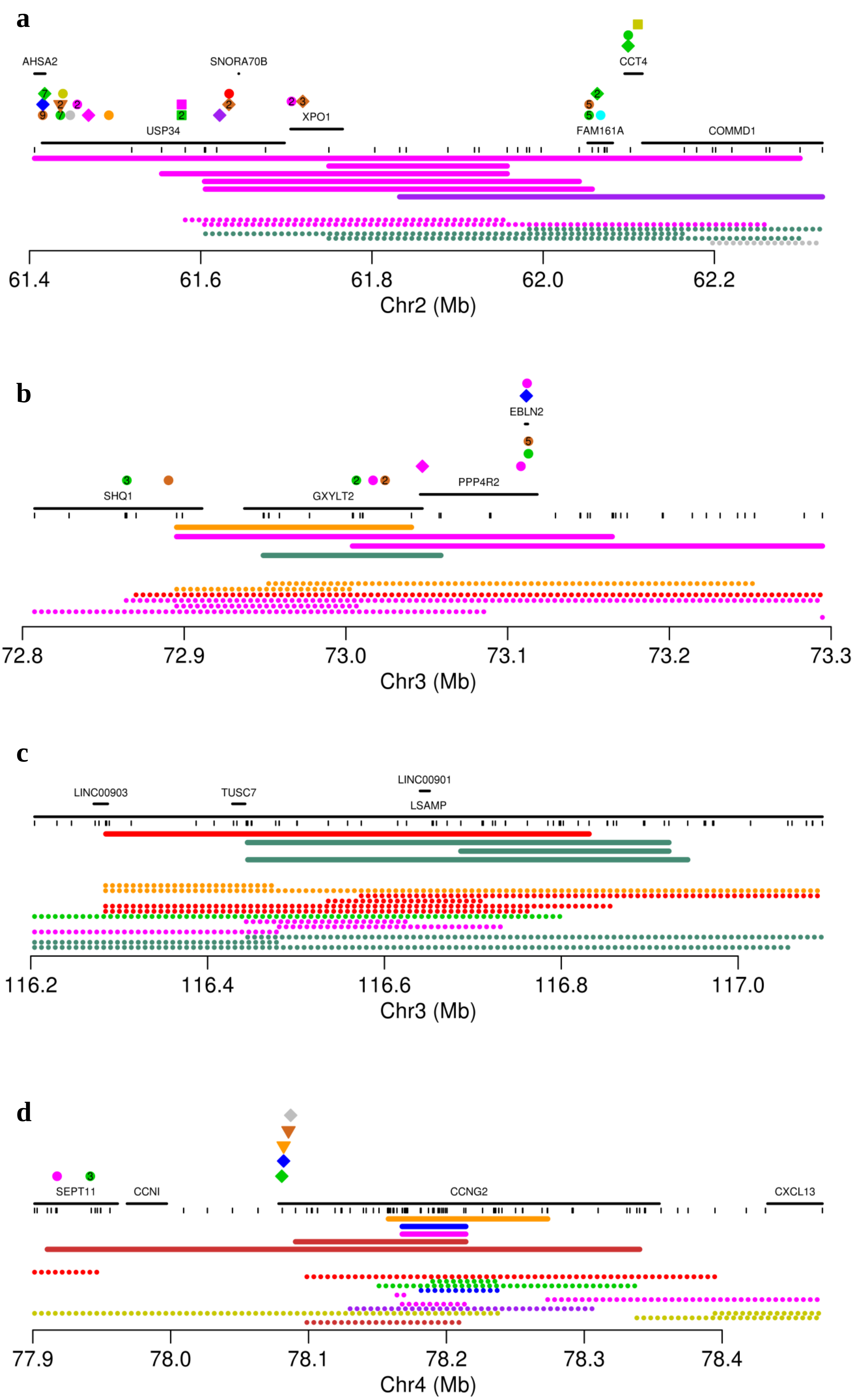
a
AHSA2
SNORA70B
CCT4
USP34
XPO1
FAM161A
COMMD1
61.4
61.6
61.8
62.0
62.2
Chr2 (Mb)
b
EBLN2
SHQ1
GXYLT2
PPP4R2
72.8
72.9
73.0
73.1
73.2
73.3
Chr3 (Mb)
c
LINC00901
LINC00903
TUSC7
LSAMP
116.2
116.4
116.6
116.8
117.0
Chr3 (Mb)
d
SEPT11
CCNI
CCNG2
CXCL13
77.9
78.0
78.1
78.2
78.3
78.4
Chr4 (Mb)

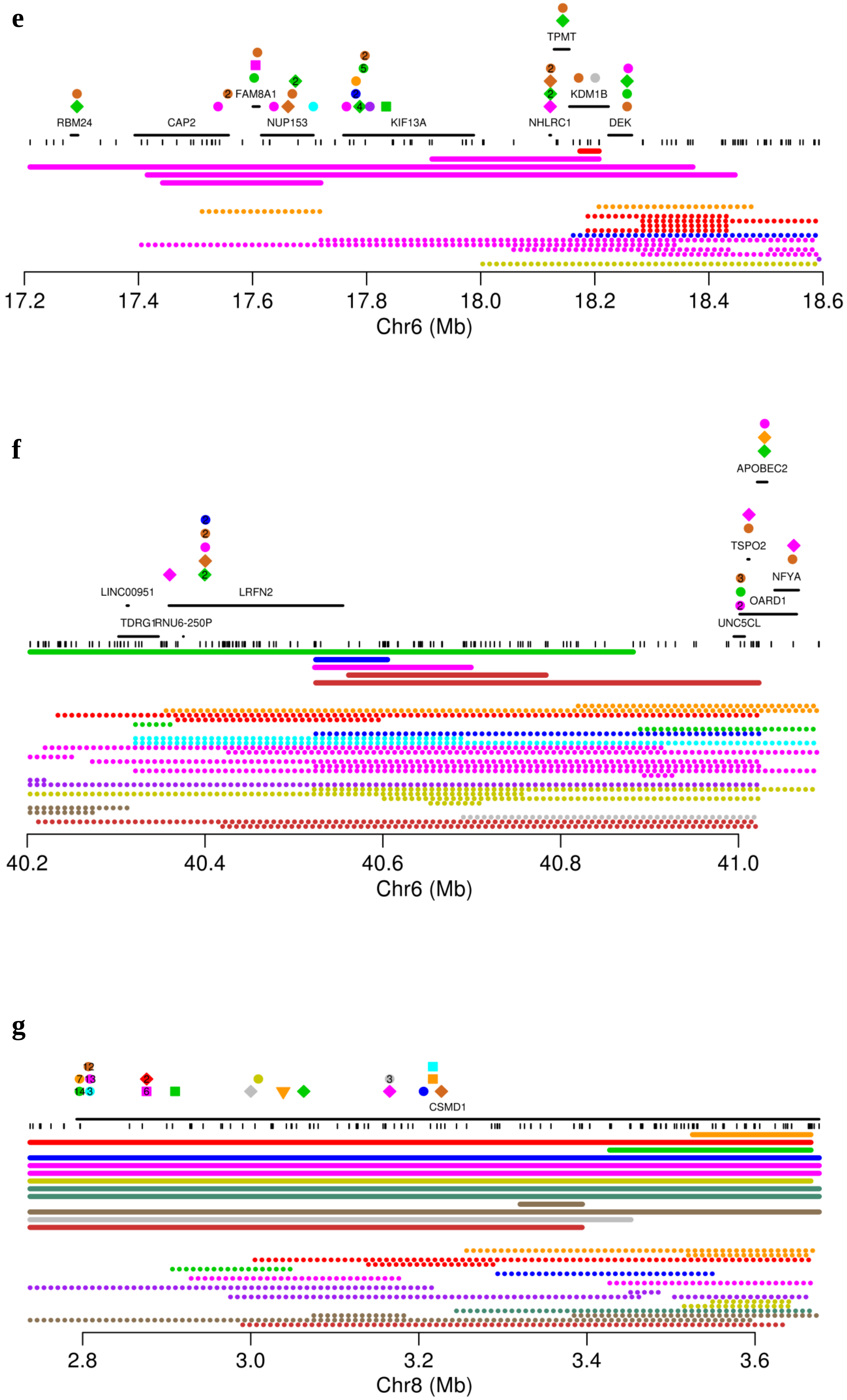
e
TPMT
FAM8A1
KDM1B
RBM24
CAP2
NUP153
KIF13A
NHLRC1
DEK
17.2
17.4
17.6
17.8
18.0
18.2
18.4
18.6
Chr6 (Mb)
f
APOBEC2
TSPO2
NFYA
LINC00951
LRFN2
OARD1
TDRG1
RNU6-250P
UNC5CL
40.2
40.4
40.6
40.8
41.0
Chr6 (Mb)
g
CSMD1
2.8
3.0
3.2
3.4
3.6
Chr8 (Mb)

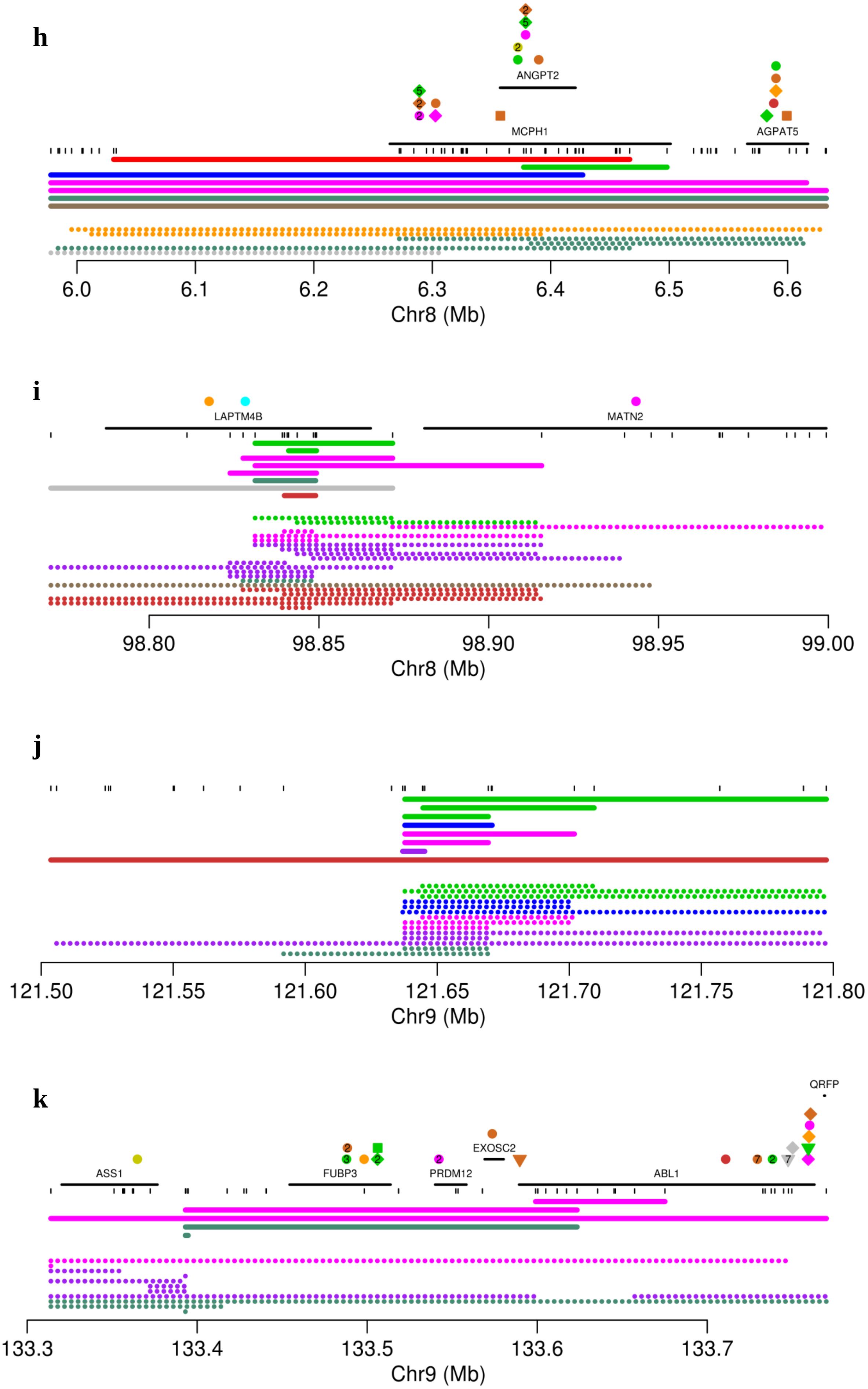
h
ANGPT2
MCPH1
AGPAT5
6.0
6.1
6.2
6.3
6.4
6.5
6.6
Chr8 (Mb)
i
LAPTM4B
MATN2
98.80
98.85
98.90
98.95
99.00
Chr8 (Mb)
j
121.50
121.55
121.60
121.65
121.70
121.75
121.80
Chr9 (Mb)
k
QRFP
EXOSC2
ASS1
FUBP3
PRDM12
ABL1
133.3
133.4
133.5
133.6
133.7
Chr9 (Mb)

l

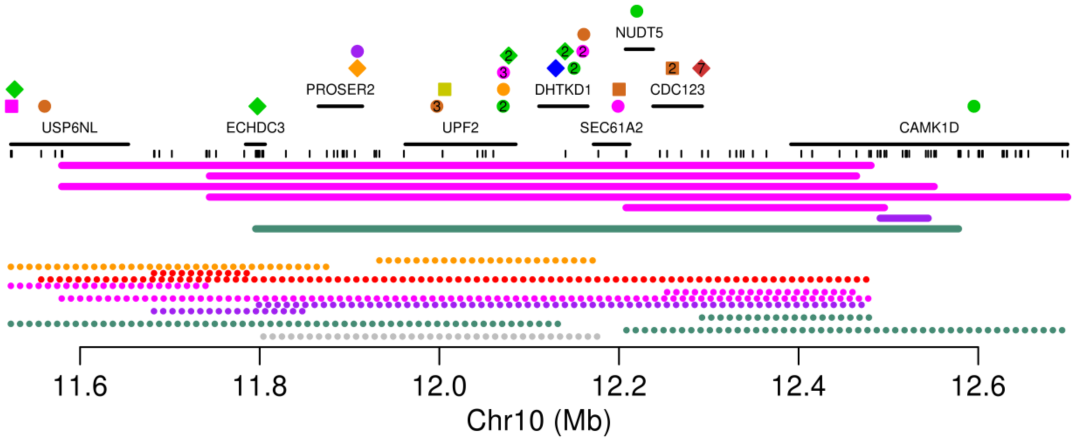
NUDT5
PROSER2
DHTKD1
CDC123
USP6NL
ECHDC3
UPF2
SEC61A2
CAMK1D
11.6
11.8
12.0
12.2
12.4
12.6
Chr10 (Mb)

m

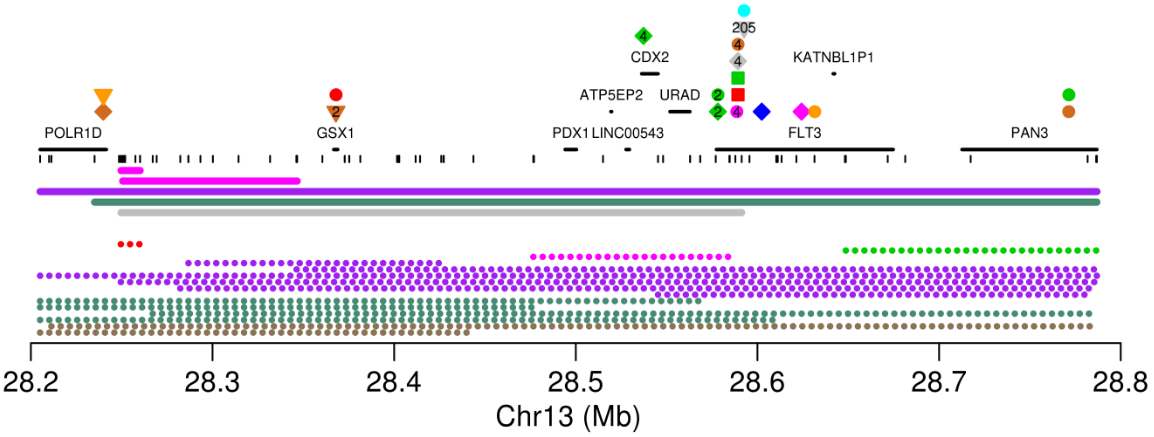
205
CDX2
KATNBL1P1
ATP5EP2
URAD
POLR1D
GSX1
PDX1
LINC00543
FLT3
PAN3
28.2
28.3
28.4
28.5
28.6
28.7
28.8
Chr13 (Mb)

n

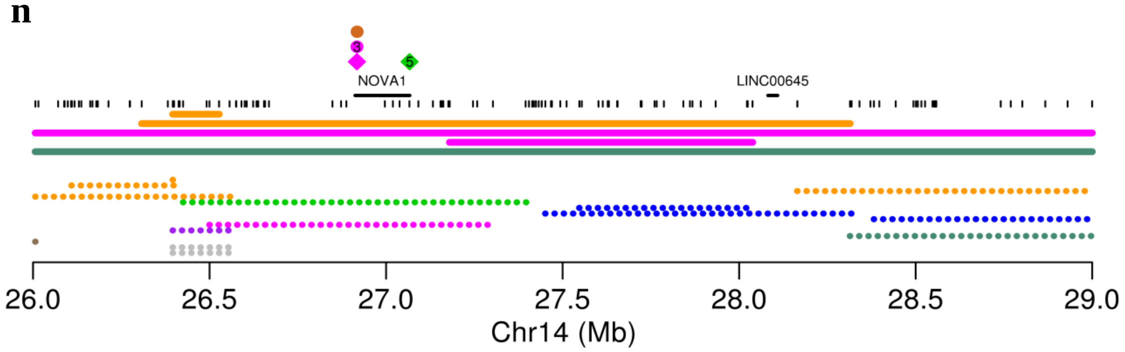
NOVA1
LINC00645
26.0
26.5
27.0
27.5
28.0
28.5
29.0
Chr14 (Mb)

o

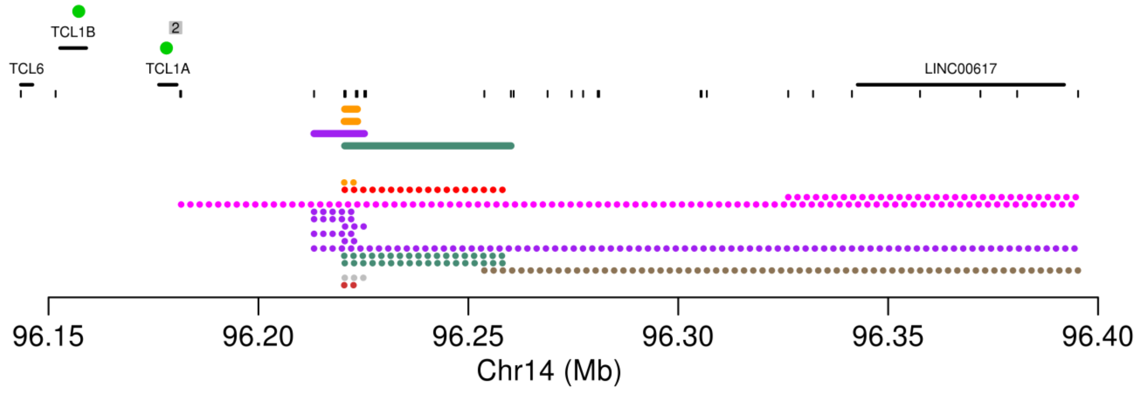

2
TCL1B
TCL6
TCL1A
LINC00617
96.15
96.20
96.25
96.30
96.35
96.40
Chr14 (Mb)

p

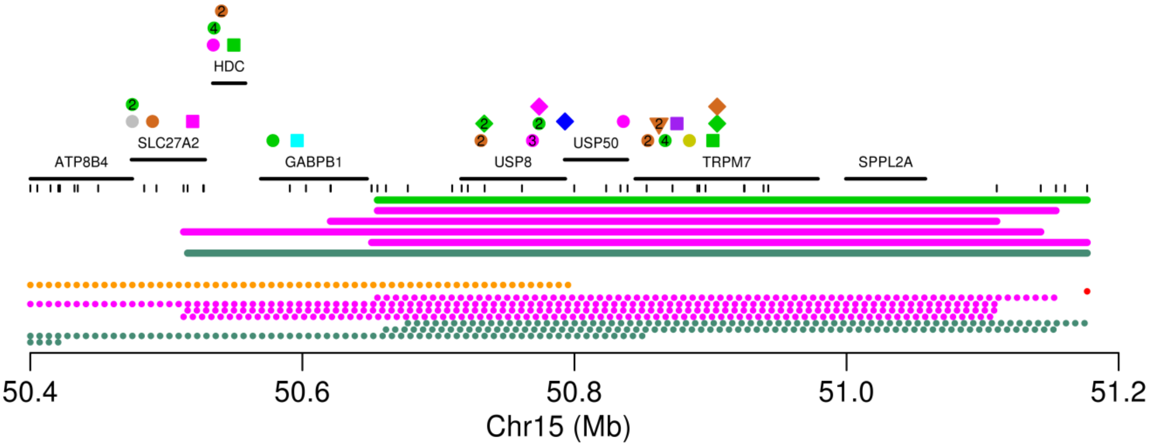

HDC
SLC27A2
ATP8B4
GABPB1
USP8
USP50
TRPM7
SPPL2A
50.4
50.6
50.8
51.0
51.2
Chr15 (Mb)

q

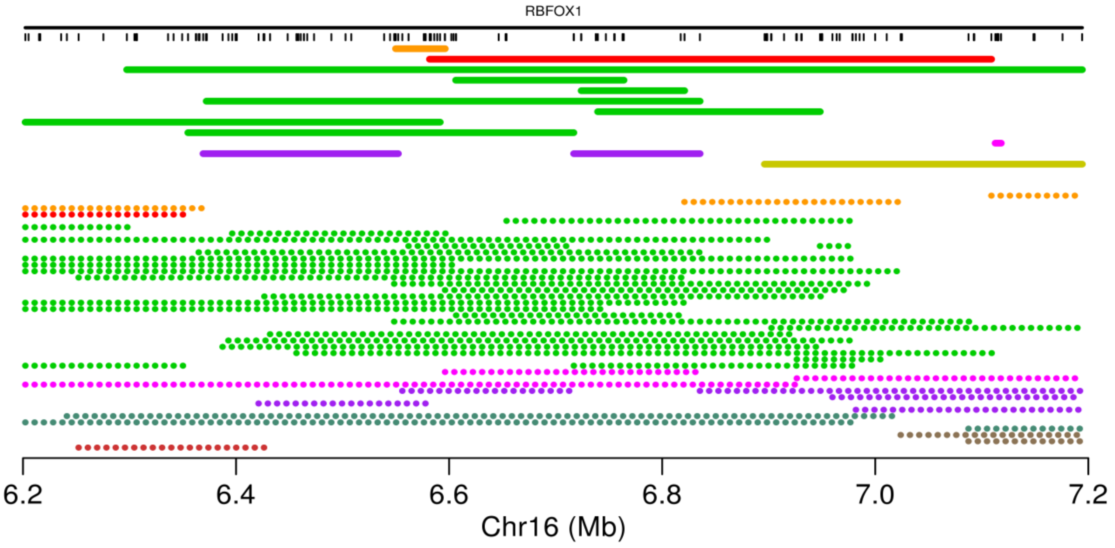

RBFOX1
6.2
6.4
6.6
6.8
7.0
7.2
Chr16 (Mb)

r

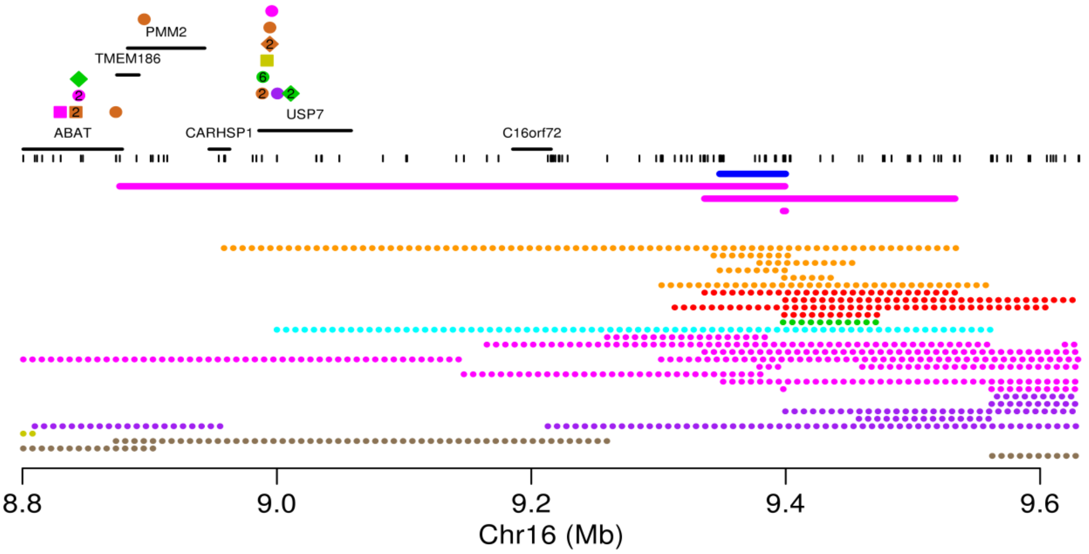
PMM2
TMEM186
ABAT
CARHSP1
USP7
C16orf72
8.8
9.0
9.2
9.4
9.6
Chr16 (Mb)

s

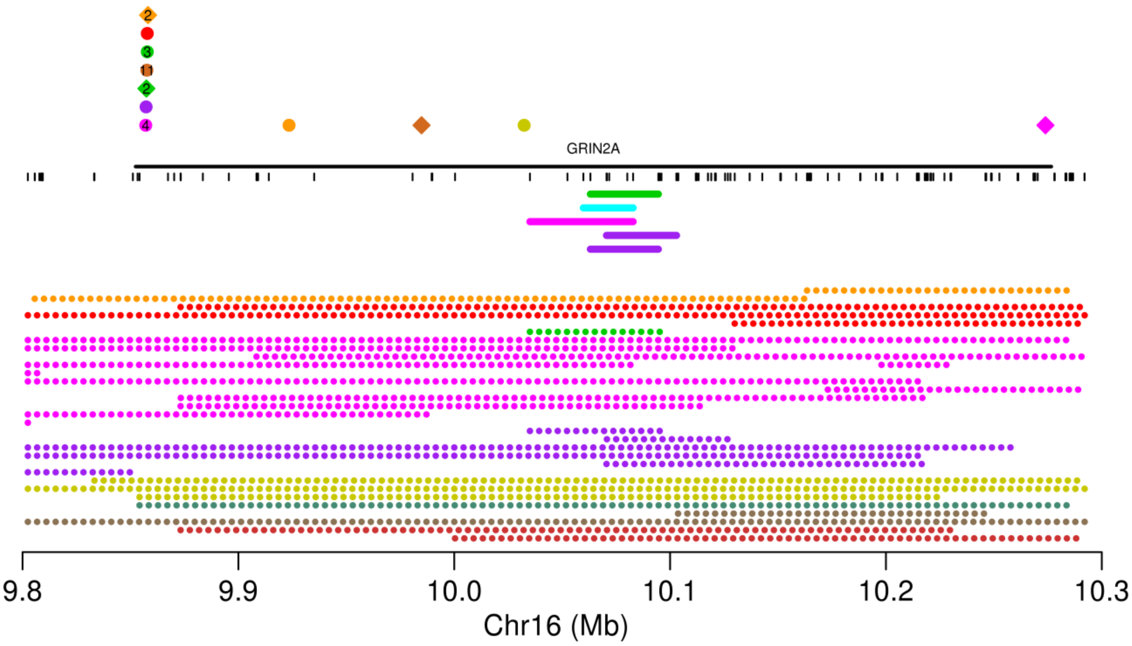
GRIN2A
9.8
9.9
10.0
10.1
10.2
10.3
Chr16 (Mb)

t

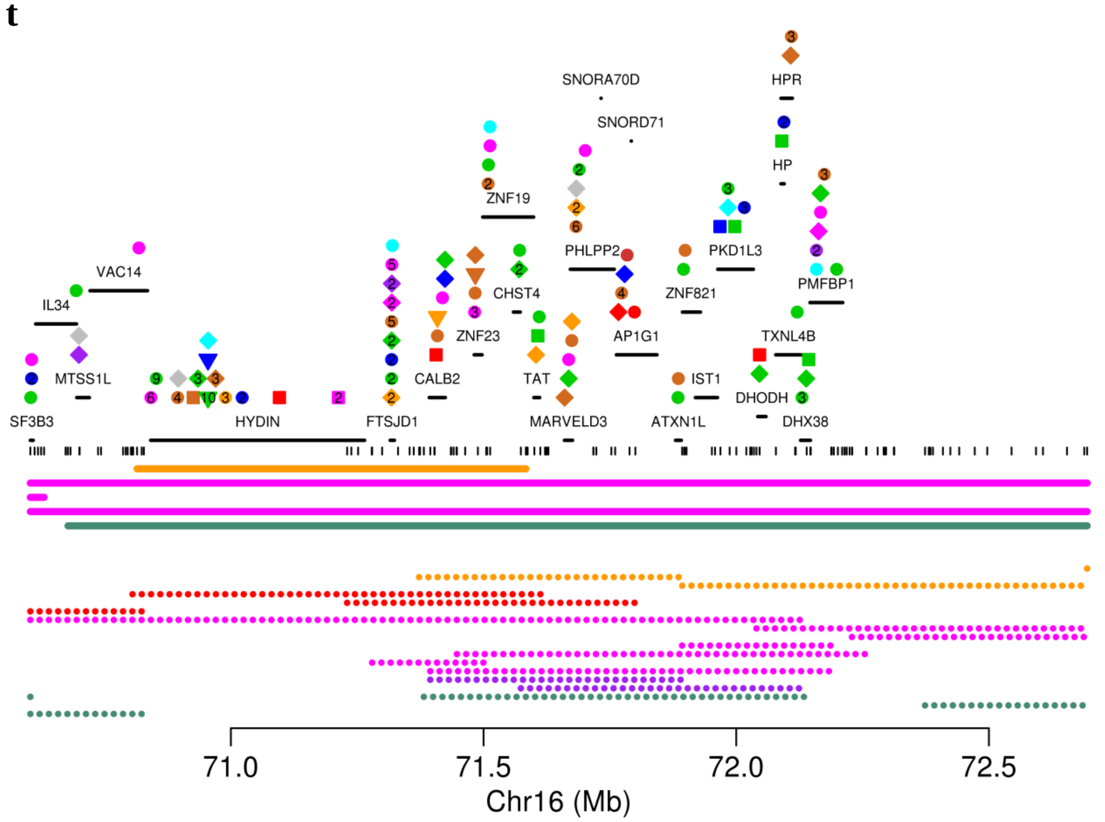
SNORA70D
HPR
SNORD71
HP
ZNF19
VAC14
IL34
PHLPP2
PKD1L3
PMFBP1
CHST4
ZNF821
ZNF23
AP1G1
TXNL4B
MTSS1L
CALB2
TAT
IST1
DHODH
SF3B3
HYDIN
FTSJD1
MARVELD3
ATXN1L
DHX38
71.0
71.5
72.0
72.5
Chr16 (Mb)

u

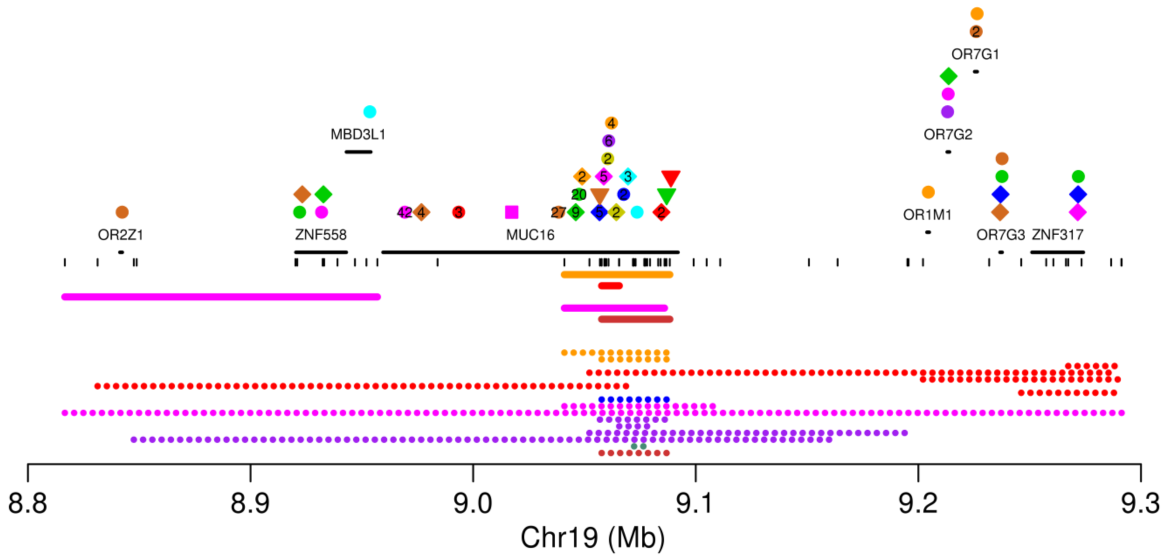
OR7G1
MBD3L1
OR7G2
OR1M1
OR2Z1
ZNF558
MUC16
OR7G3
ZNF317
8.8
8.9
9.0
9.1
9.2
9.3
Chr19 (Mb)

v

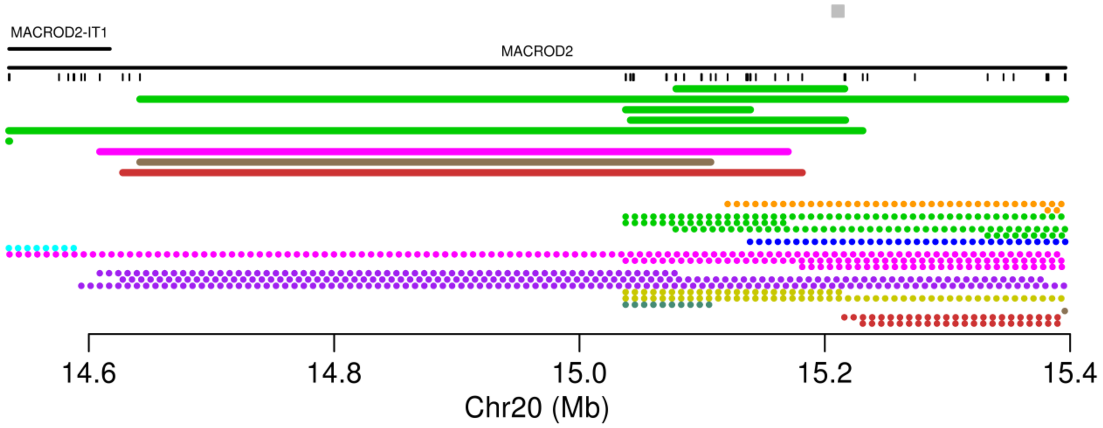

MACROD2-IT1
MACROD2
14.6
14.8
15.0
15.2
15.4
Chr20 (Mb)

w

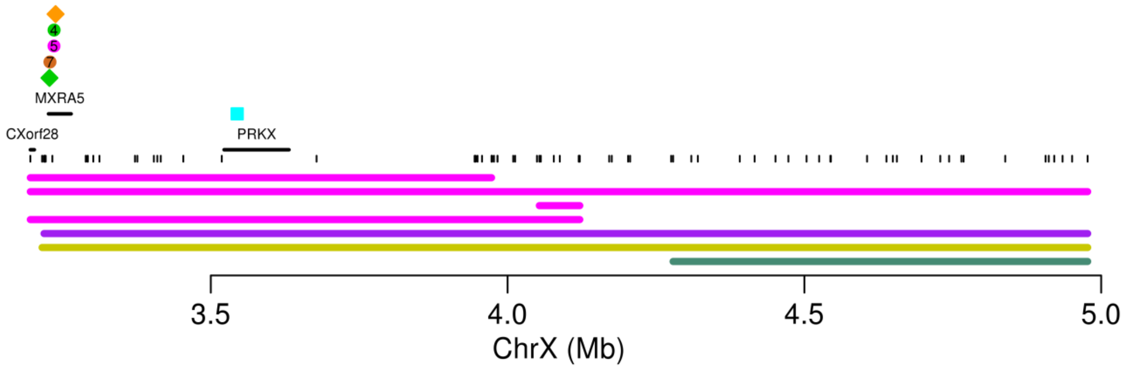

MXRA5
CXorf28
PRKX
3.5
4.0
4.5
5.0
ChrX (Mb)

x

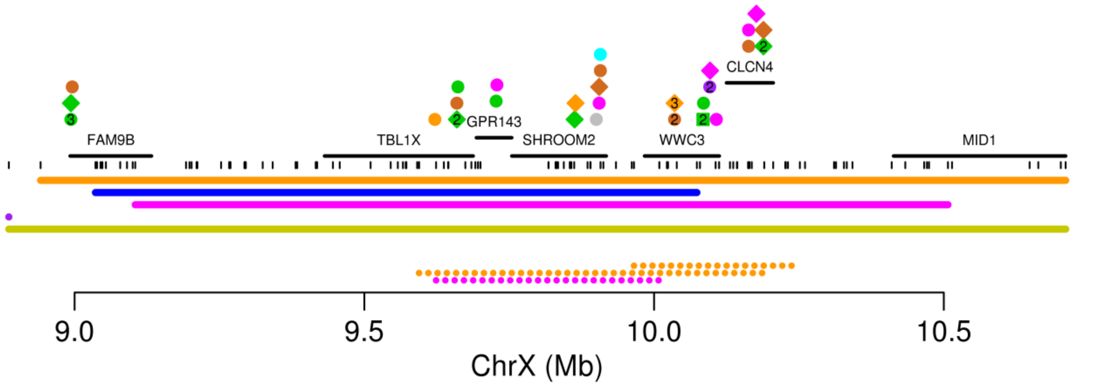

CLCN4
GPR143
FAM9B
TBL1X
SHROOM2
WWC3
MID1
9.0
9.5
10.0
10.5
ChrX (Mb)

Supplementary Fig. 7: Homozygous deletions targeting 9 unstable (sub)telomeres

Positions of genes are indicated (black lines), as well as truncating mutations annotated in COSMIC, coloured according to tumour type and with symbols according to mutation type (nonsense, essential splice site, frame-shift insertion or deletion, in-frame insertion or deletion). When multiple somatic mutations in the same tumour type are annotated close together in COSMIC, their numbers are shown. Array probe positions are depicted below the genes. Homozygous deletions are shown as bold lines and small hemizygous deletions as dotted lines, both coloured according to tumour type. (**a**) chr2:0.92-0.94, (**b**) chr6:170.76-170.91, (**c**) chr7:158.91-159.13, (**d**) chr8:0.42-0.78, (**e**) chr9:0.76-0.88, (**f**) chr13:113.09-115.05, (**g**) chr17:80.94-81.01, (**h**) chr18:76.71-77.80, (**i**) chrX:0.10-1.

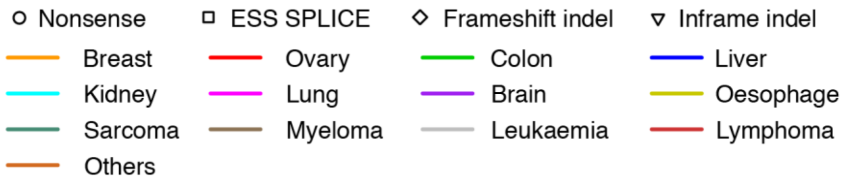

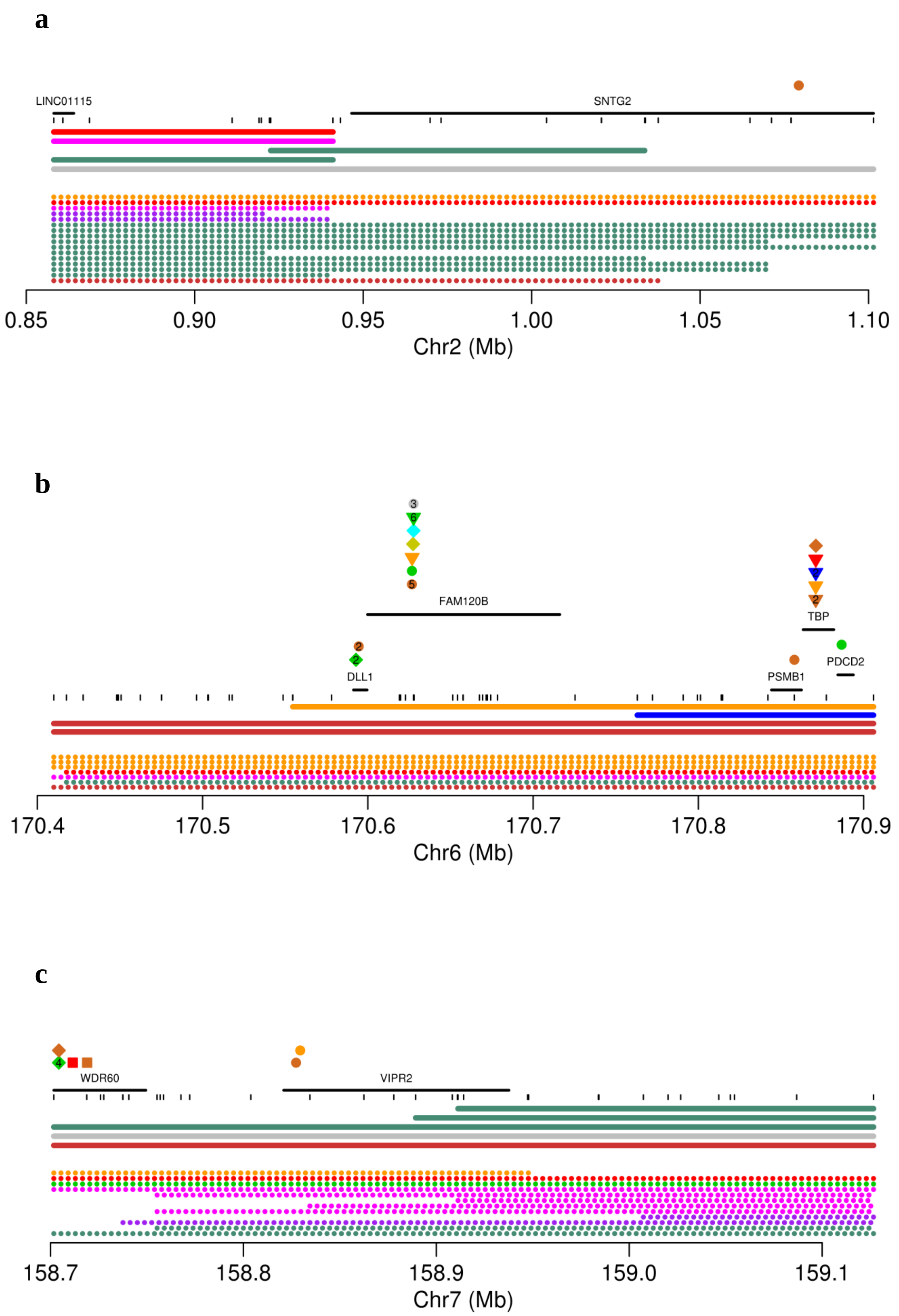
a
LINC01115
SNTG2
0.85
0.90
0.95
1.00
1.05
1.10
Chr2 (Mb)
b
FAM120B
TBP
DLL1
PSMB1
PDCD2
170.4
170.5
170.6
170.7
170.8
170.9
Chr6 (Mb)
c
WDR60
VIPR2
158.7
158.8
158.9
159.0
159.1
Chr7 (Mb)

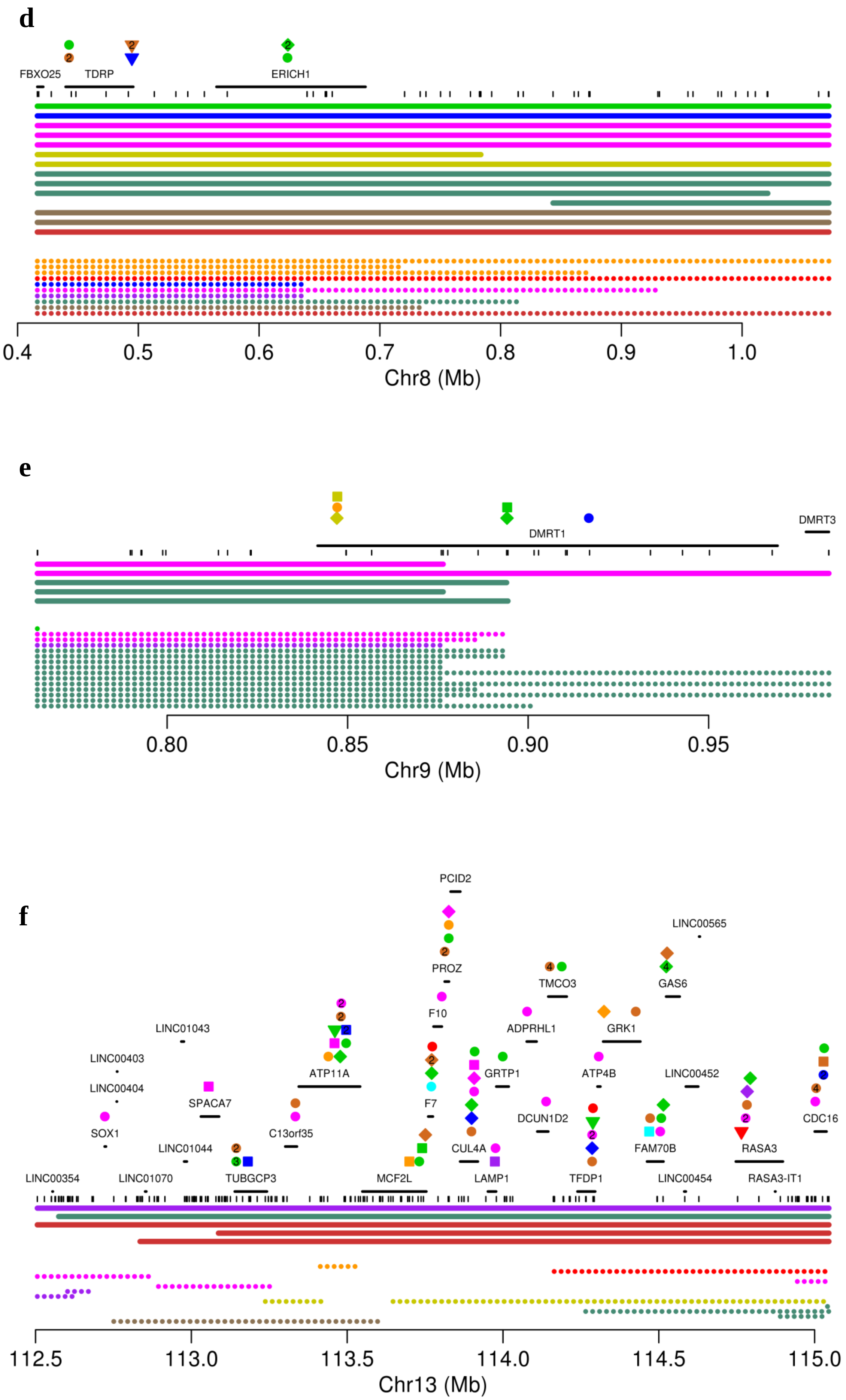
d
FBXO25
TDRP
ERICH1
0.4
0.5
0.6
0.7
0.8
0.9
1.0
Chr8 (Mb)
e
DMRT1
DMRT3
0.80
0.85
0.90
0.95
Chr9 (Mb)
f
PCID2
LINC00565
PROZ
TMCO3
GAS6
F10
ADPRHL1
GRK1
LINC01043
LINC00403
ATP11A
GRTP1
ATP4B
LINC00452
LINC00404
SPACA7
F7
DCUN1D2
CDC16
SOX1
C13orf35
CUL4A
FAM70B
RASA3
LINC01044
TUBGCP3
MCF2L
LAMP1
TFDP1
LINC00454
RASA3-IT1
LINC00354
LINC01070
112.5
113.0
113.5
114.0
114.5
115.0
Chr13 (Mb)

g

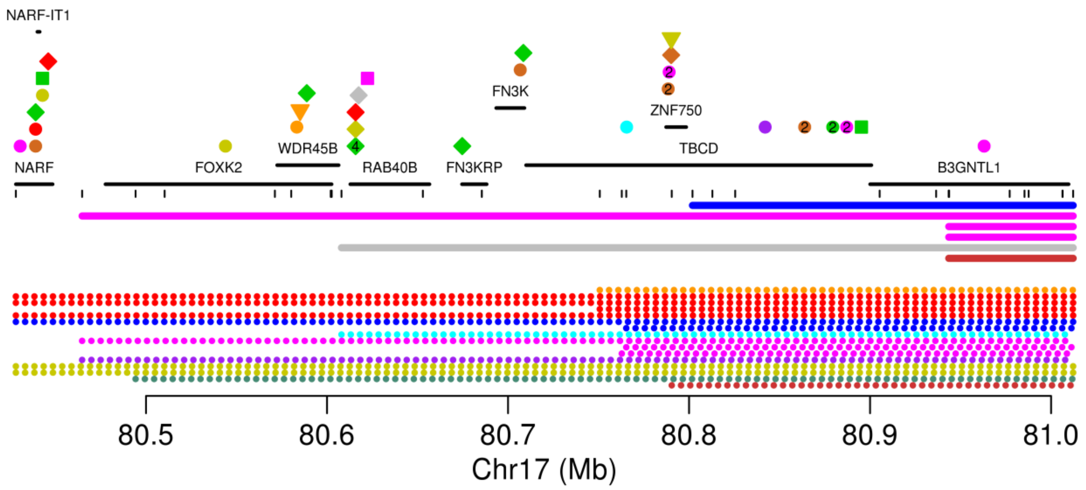
NARF-IT1
NARF
FOXK2
WDR45B
RAB40B
FN3KRP
FN3K
ZNF750
TBCD
B3GNTL1
80.5
80.6
80.7
80.8
80.9
81.0
Chr17 (Mb)

h

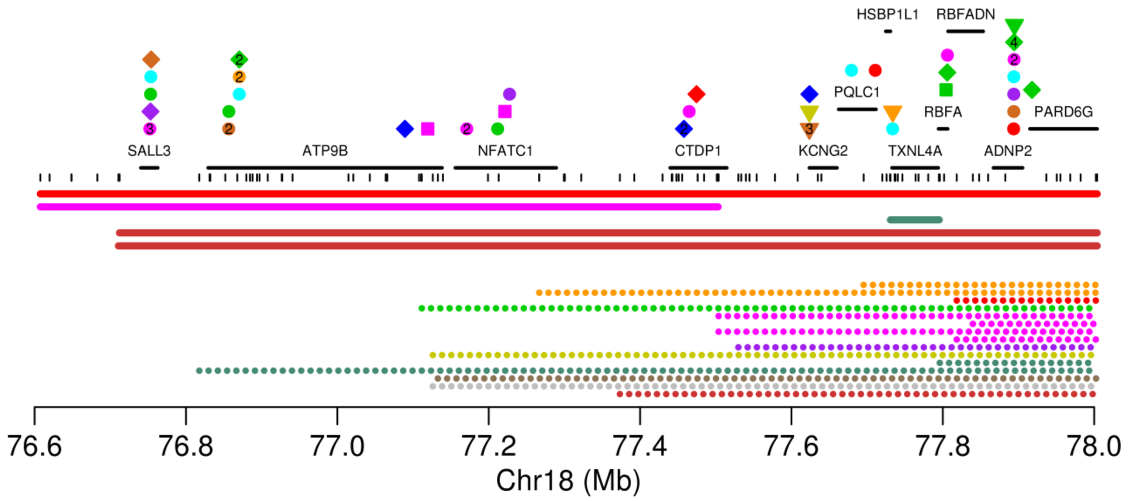
HSBP1L1
RBFADN
PQLC1
RBFA
PARD6G
SALL3
ATP9B
NFATC1
CTDP1
KCNG2
TXNL4A
ADNP2
76.6
76.8
77.0
77.2
77.4
77.6
77.8
78.0
Chr18 (Mb)

i

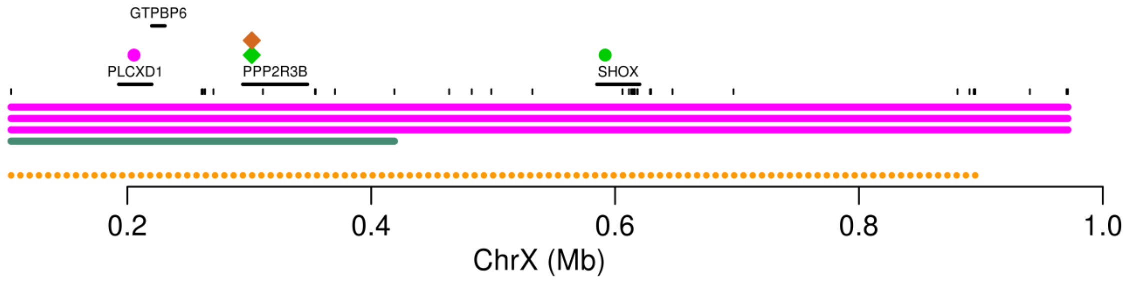
GTPBP6
PLCXD1
PPP2R3B
SHOX
0.2
0.4
0.6
0.8
1.0
ChrX (Mb)

Supplementary Fig. 8: Homozygous deletions targeting 6 regions showing signatures of positive selection but without a clear target gene

Positions of genes are indicated (black lines), as well as truncating mutations annotated in COSMIC, coloured according to tumour type and with symbols according to mutation type (nonsense, essential splice site, frame-shift insertion or deletion, in-frame insertion or deletion). When multiple somatic mutations in the same tumour type are annotated close together in COSMIC, their numbers are shown. Array probe positions are depicted below the genes. Homozygous deletions are shown as bold lines and small hemizygous deletions as dotted lines, both coloured according to tumour type. (**a**) chr4:99.06-99.14, (**b**) chr4:182.34-182.70, (**c**) chr8:34.29-34.30, (**d**) chr13:20.34-20.45, (**e**) chr13:39.54-39.54, (**f**) chr17:0.01-0.05.

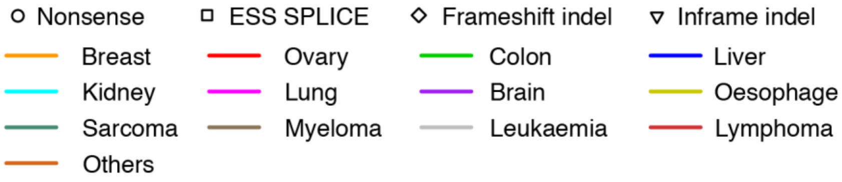

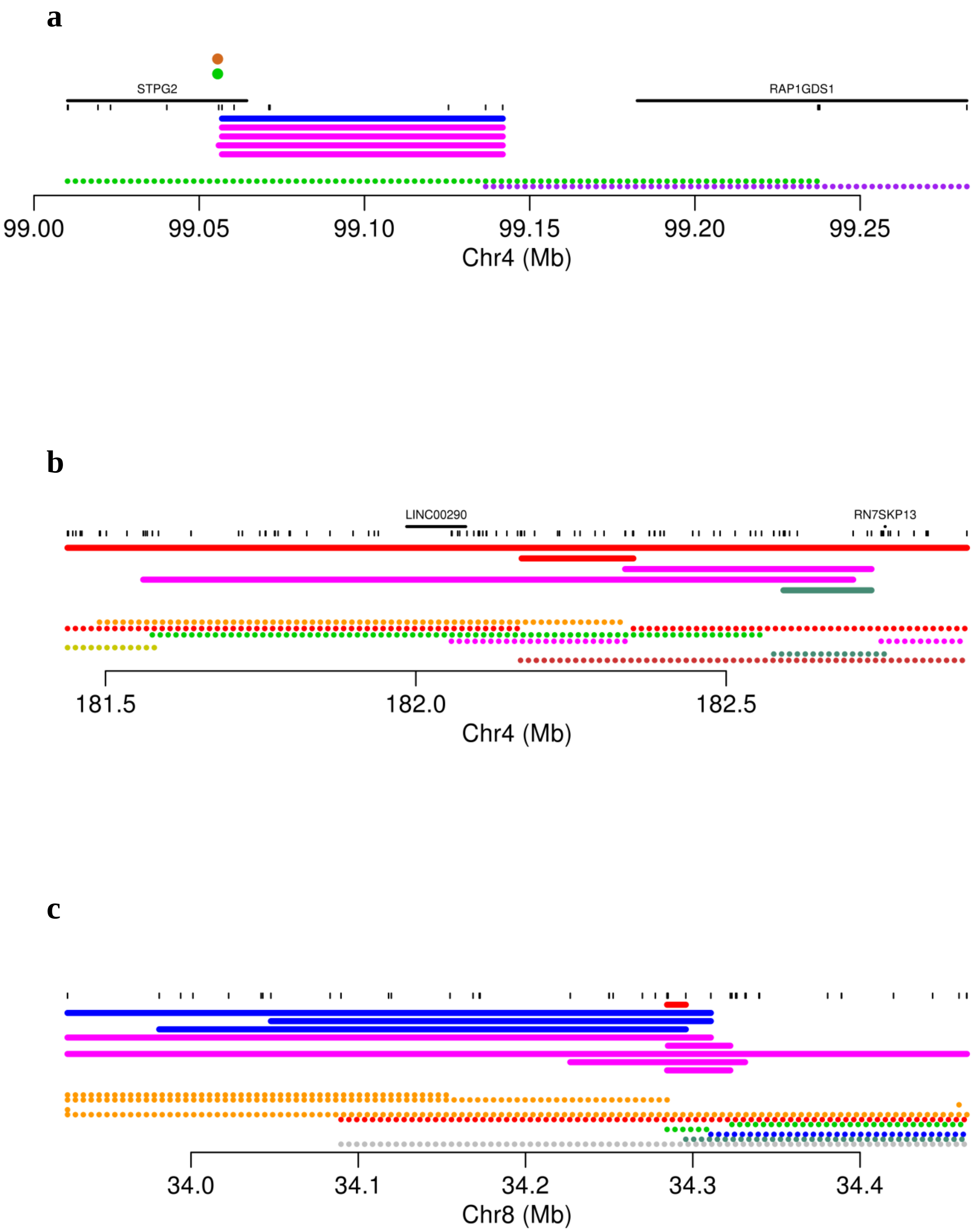
a
STPG2
RAP1GDS1
99.00
99.05
99.10
99.15
99.20
99.25
Chr4 (Mb)
b
LINC00290
RN7SKP13
181.5
182.0
182.5
Chr4 (Mb)
c
34.0
34.1
34.2
34.3
34.4
Chr8 (Mb)

d

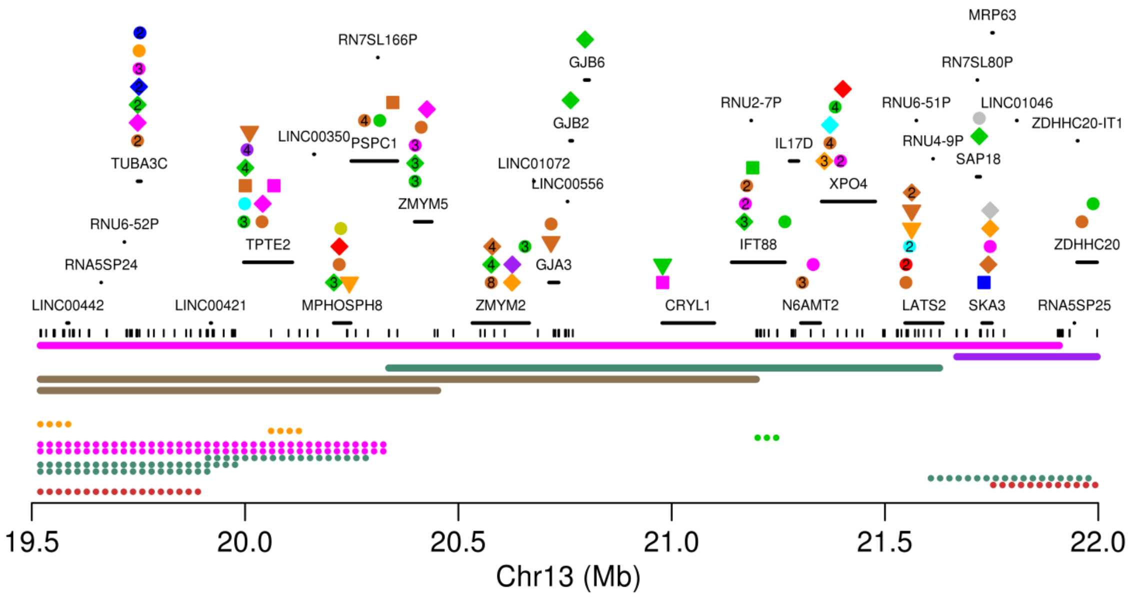
MRP63
RN7SL166P
GJB6
RN7SL80P
LINC01046
ZDHHC20-IT1
RNU2-7P
RNU6-51P
RNU4-9P
GJB2
IL17D
SAP18
TUBA3C
LINC00350
PSPC1
LINC01072
LINC00556
XPO4
ZMYM5
RNU6-52P
TPTE2
IFT88
ZDHHC20
RNA5SP24
GJA3
LINC00442
LINC00421
MPHOSPH8
ZMYM2
CRYL1
N6AMT2
LATS2
SKA3
RNA5SP25
19.5
20.0
20.5
21.0
21.5
22.0
Chr13 (Mb)

e

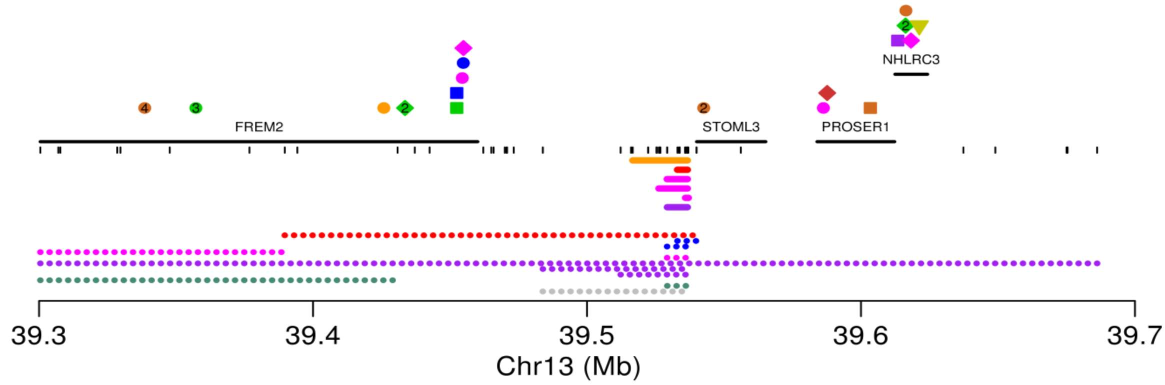
NHLRC3
FREM2
STOML3
PROSER1
39.3
39.4
39.5
39.6
39.7
Chr13 (Mb)

f

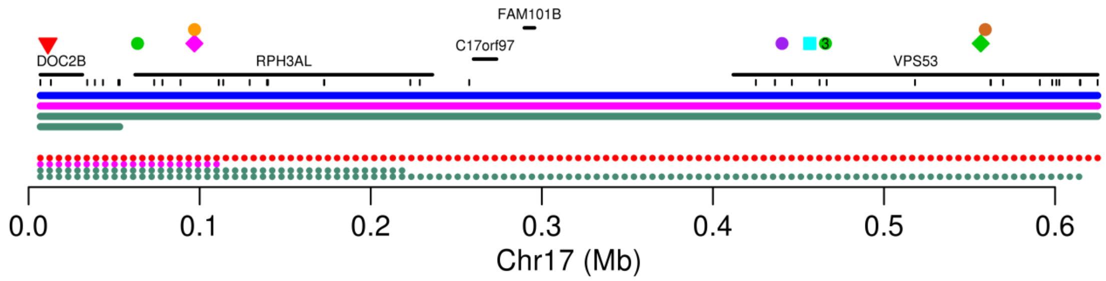
FAM101B
C17orf97
DOC2B
RPH3AL
VPS53
0.0
0.1
0.2
0.3
0.4
0.5
0.6
Chr17 (Mb)

Supplementary Fig. 9: Homozygous deletions targeting 27 candidate tumour suppressors

Positions of genes are indicated (black lines), as well as truncating mutations annotated in COSMIC, coloured according to tumour type and with symbols according to mutation type (nonsense, essential splice site, frame-shift insertion or deletion, in-frame insertion or deletion). When multiple somatic mutations in the same tumour type are annotated close together in COSMIC, their numbers are shown. Array probe positions are depicted below the genes. Homozygous deletions are shown as bold lines and small hemizygous deletions as dotted lines, both coloured according to tumour type. (**a**) *CASP9*, (**b**) *ARHGEF10L*, (**c**) *IGF2BP2*, (**d**) *N4BP*, (**e**) *HELQ* and *FAM175A*, (**f**) *CASP3*, (**g**) *LINC01060*, (**h**) *PDE4D*, (**i**) *RAD17*, (**j**) *ARHGEF10*, (**k**) *LEPROTL1*, (**l**) *PTPRD*, (**m**) *KAT6B*, (**n**) *CPEB3*, (**o**) *MGMT*, (**p**) *KIAA1551*, (**q**) *USP44*, (**r**) *SETD1B*, (**s**) *LINC00375*, (**t**) *GPC5*, (**u**) *SOX21*, (**v**) *BAZ1A*, (**w**) *MIDEAS* (**x**) *RFWD3*, (**y**) *MAFTRR* and (**z**) *LINC00662*.

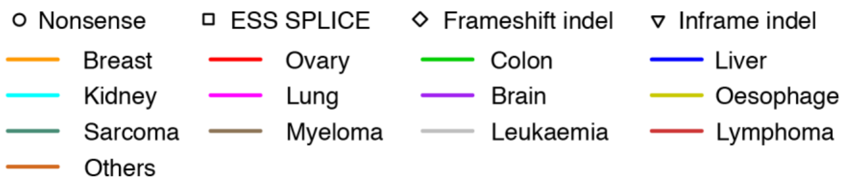

a *CASP9*

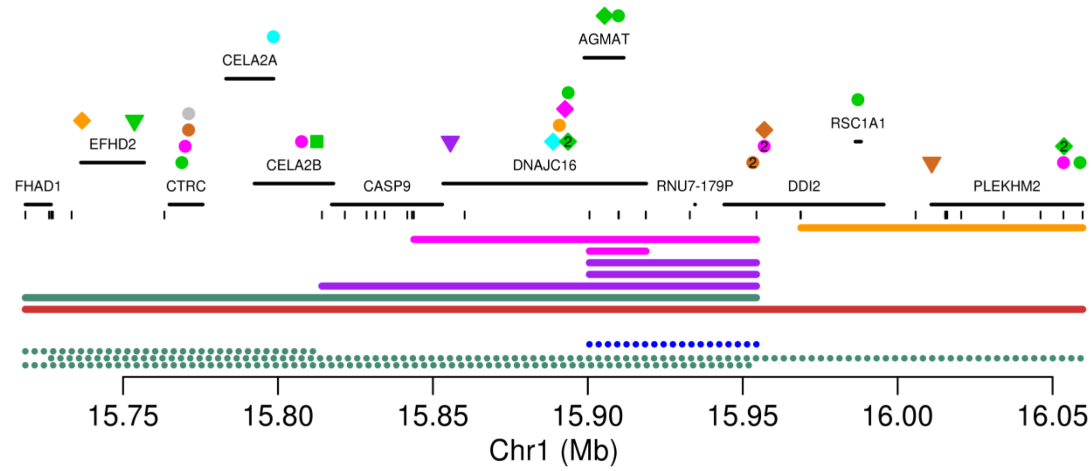

b *ARHGEF10L*

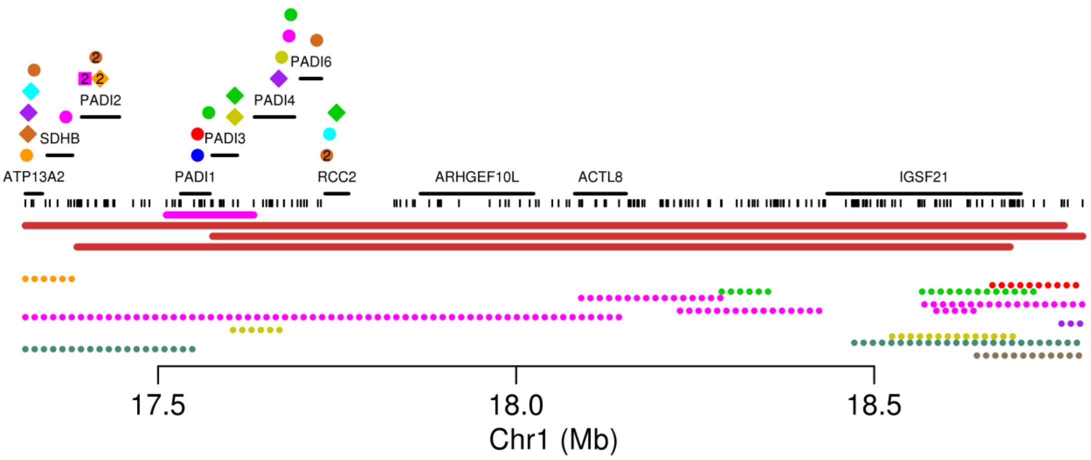

c *IGF2BP2*

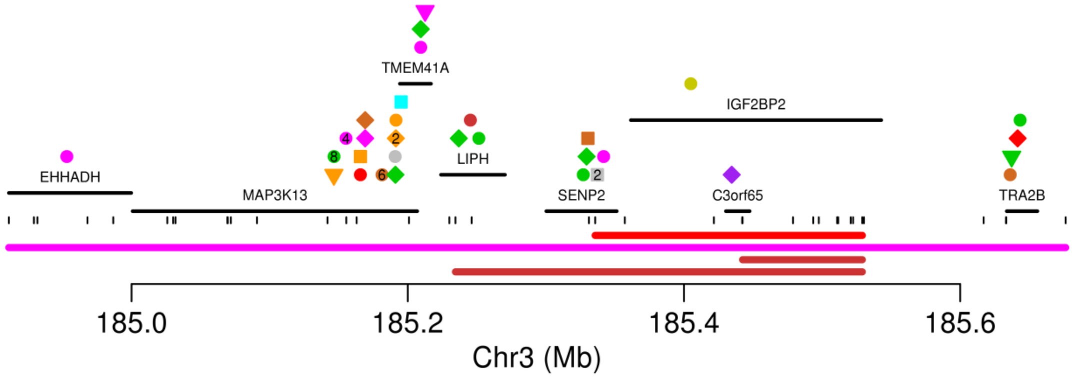

d *N4BP*

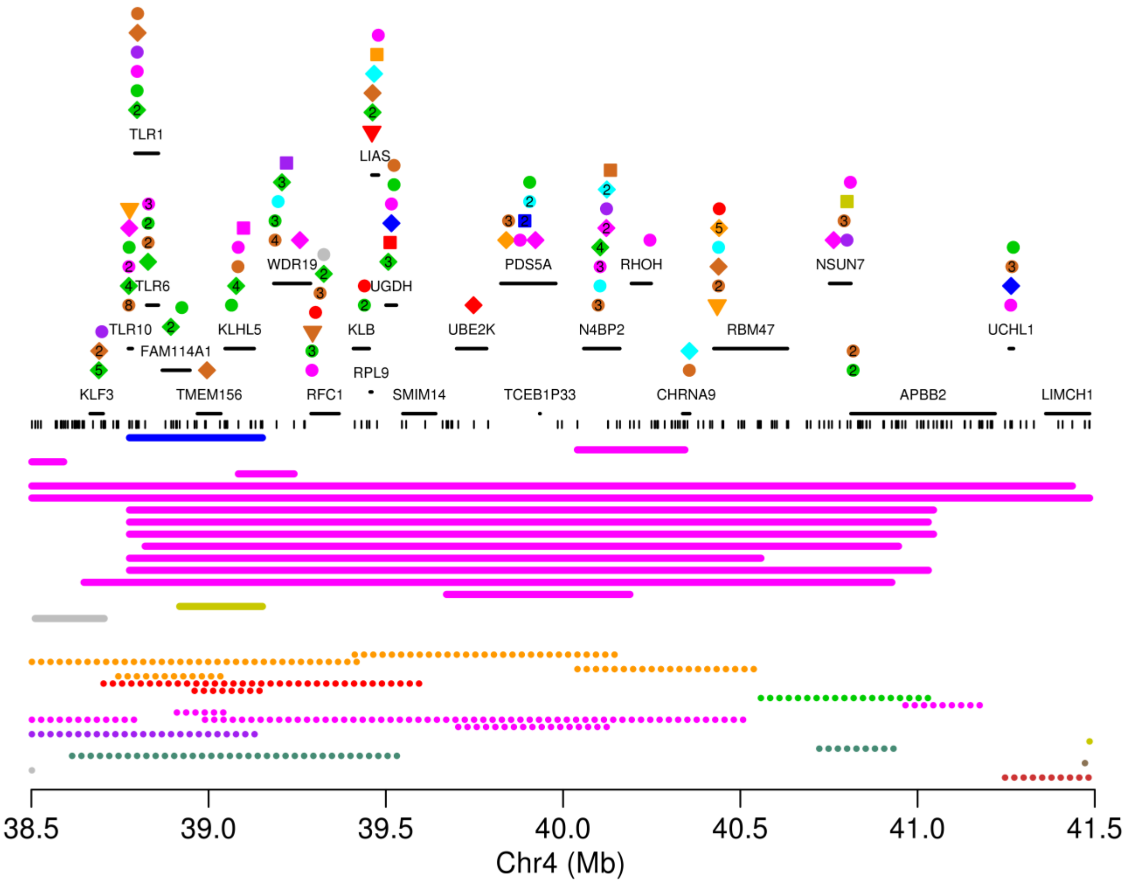

e *HELQ/FAM175A*

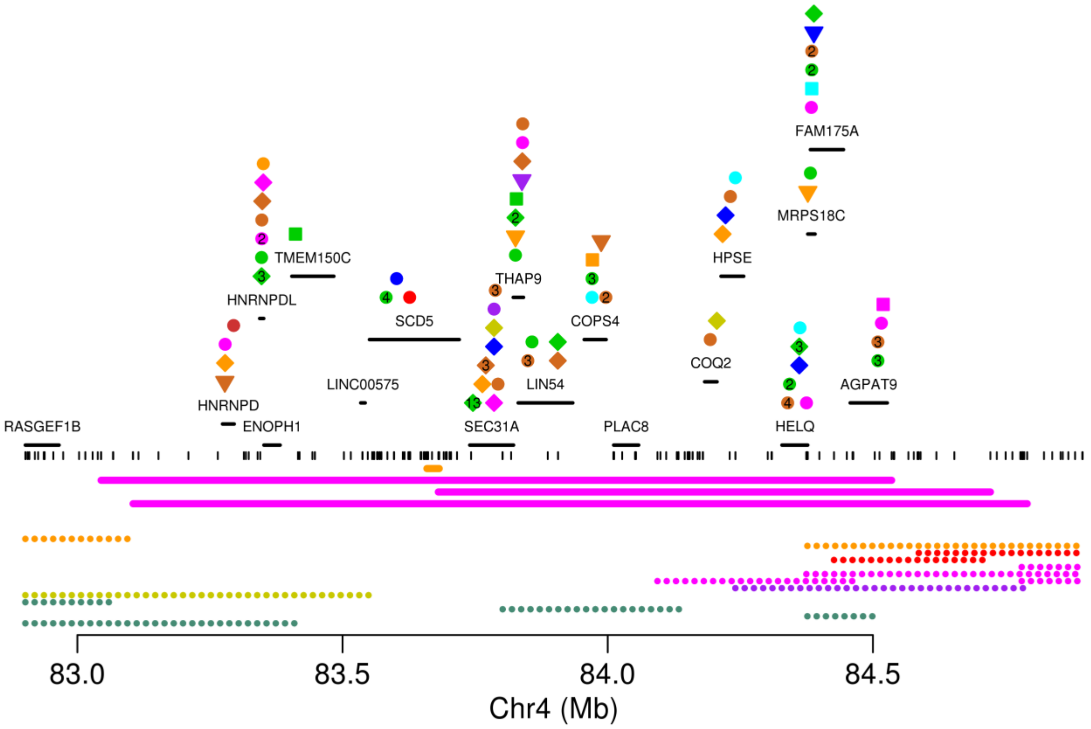

f *CASP3*

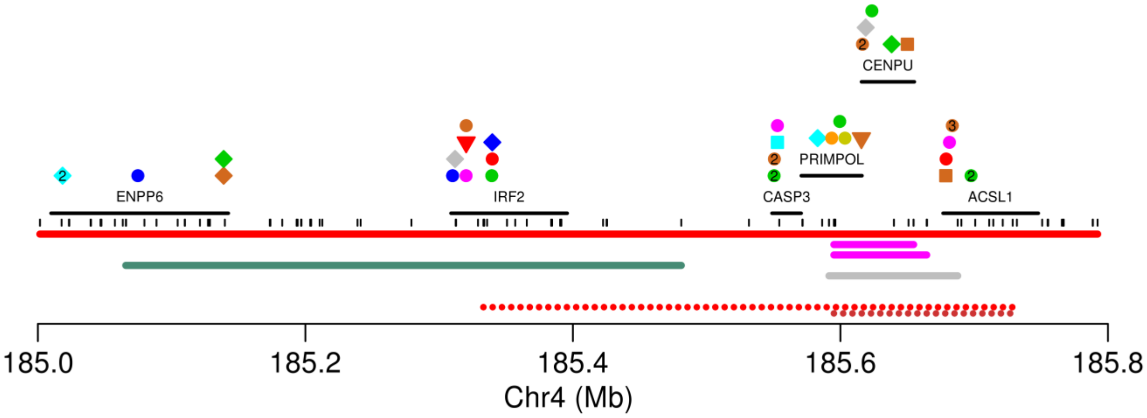

g *LINC01060*

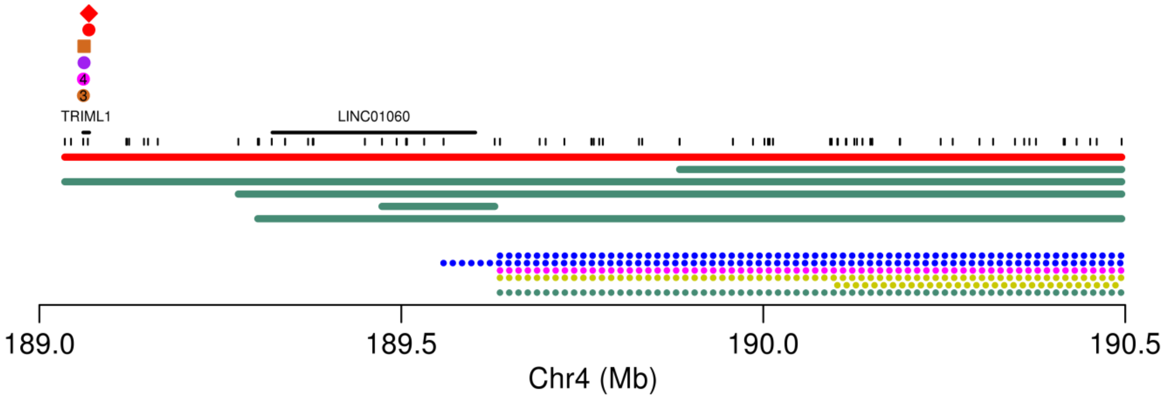

h *PDE4D*

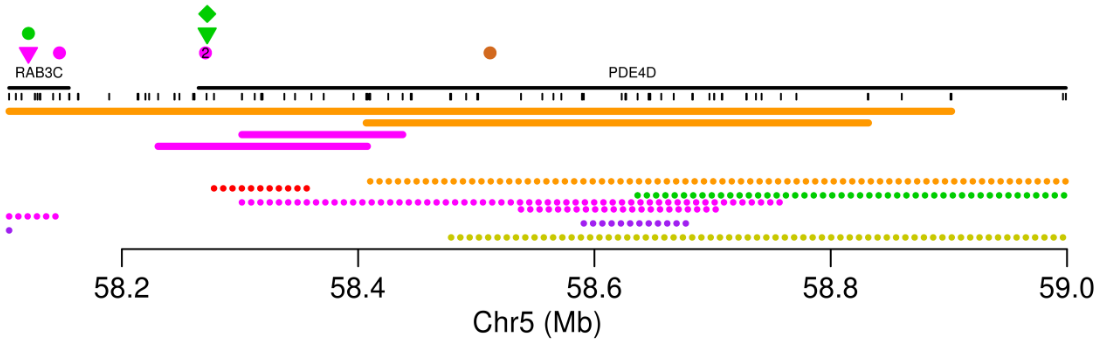

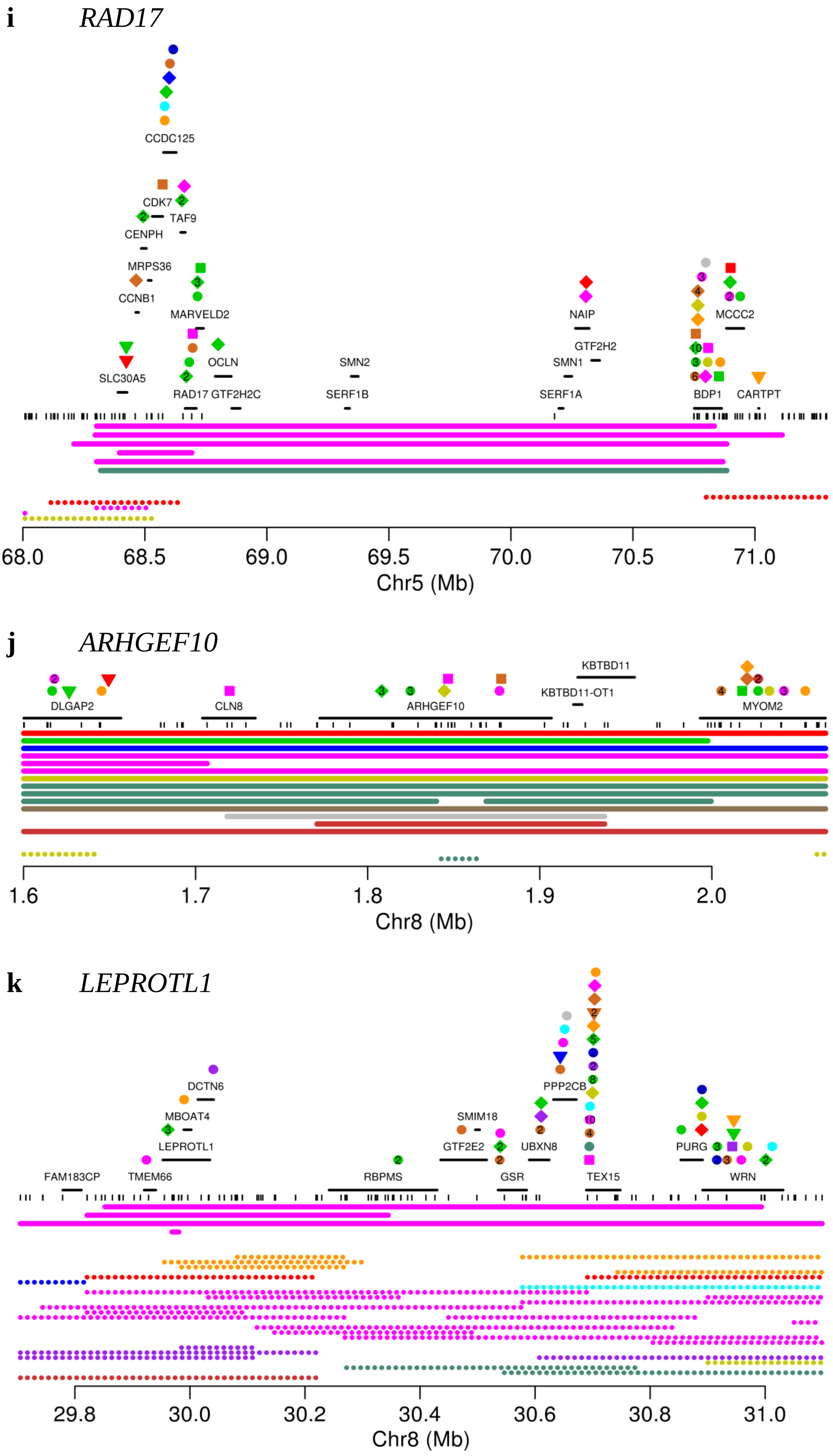
i RAD17
CCDC125
CDK7
TAF9
CENPH
MRPS36
CCNB1
MARVELD2
NAIP
MCCC2
GTF2H2
OCLN
SMN2
SMN1
SLC30A5
RAD17 GTF2H2C
SERF1B
SERF1A
BDP1
CARTPT
68.0
68.5
69.0
69.5
70.0
70.5
71.0
Chr5 (Mb)
j ARHGEF10
KBTBD11
DLGAP2
CLN8
ARHGEF10
KBTBD11-OT1
MYOM2
1.6
1.7
1.8
1.9
2.0
Chr8 (Mb)
k LEPROTL1
DCTN6
PPP2CB
MBOAT4
SMIM18
LEPROTL1
GTF2E2
UBXN8
PURG
FAM183CP
TMEM66
RBPMS
GSR
TEX15
WRN
29.8
30.0
30.2
30.4
30.6
30.8
31.0
Chr8 (Mb)

l *PTPRD*

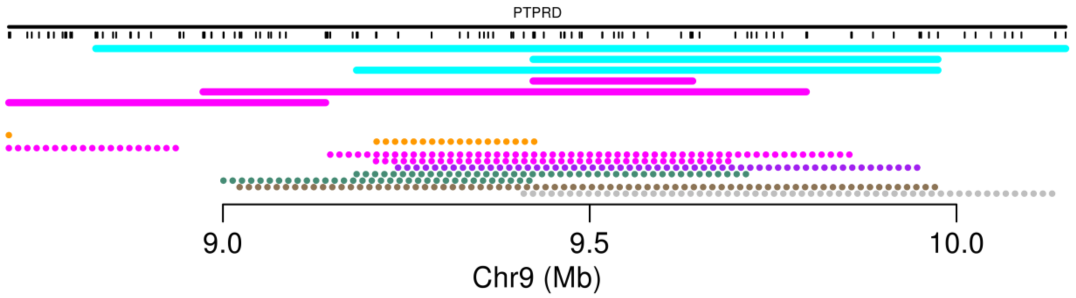

m *KAT6B*

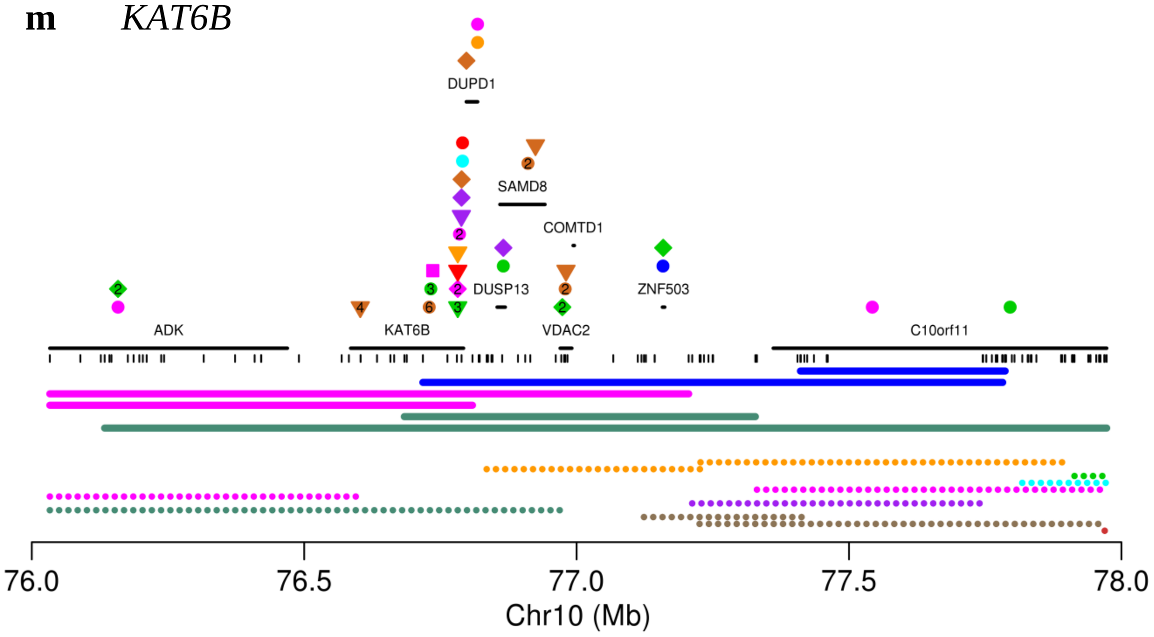

n *CPEB3*

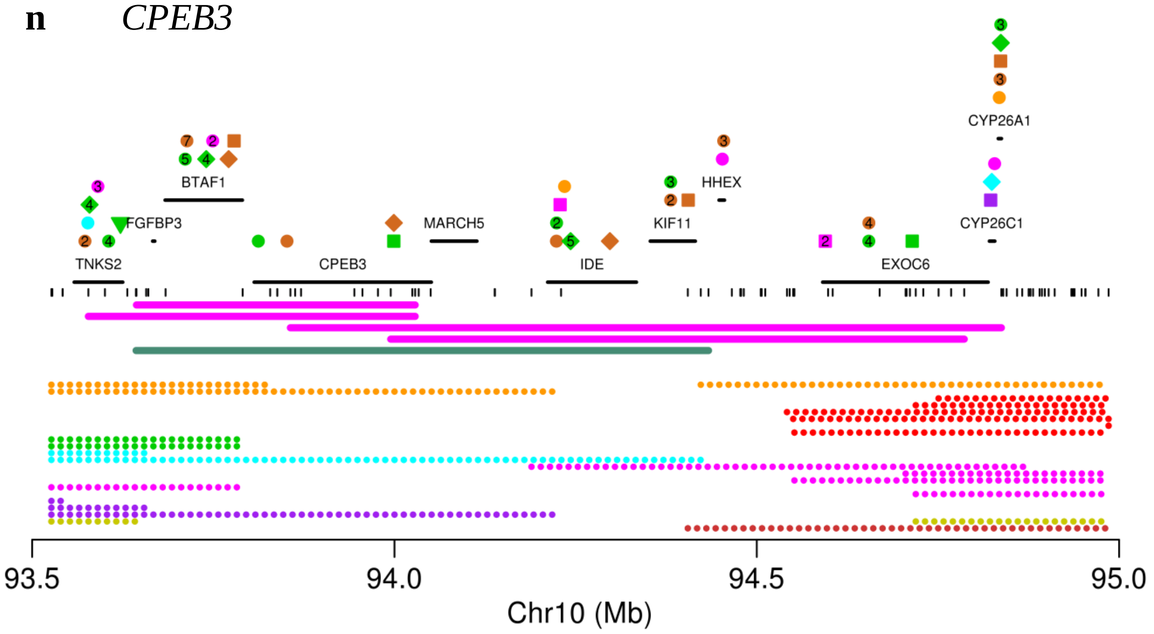

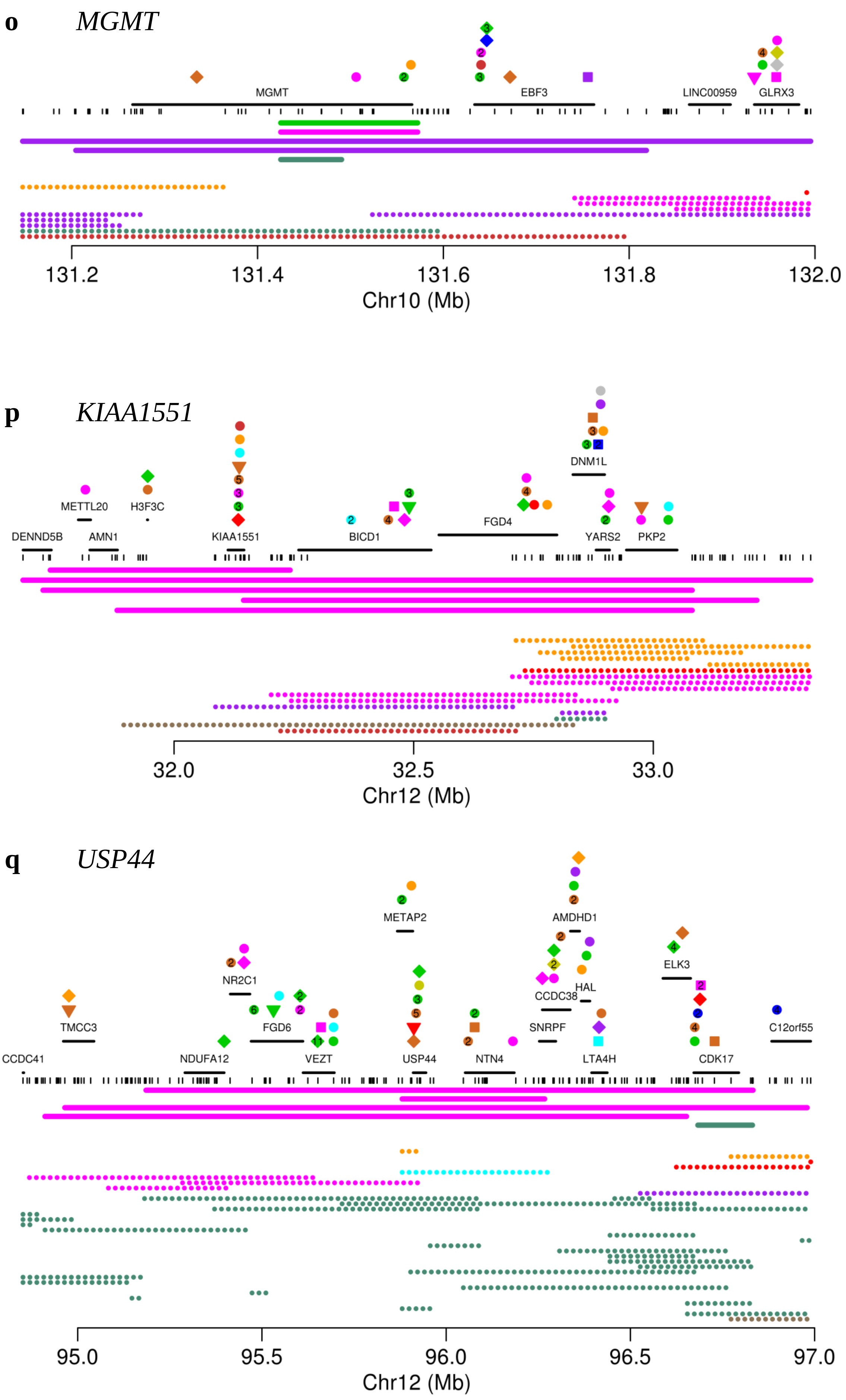

o
MGMT
MGMT
EBF3
LINC00959
GLRX3
131.2
131.4
131.6
131.8
132.0
Chr10 (Mb)
p
KIAA1551
DNM1L
METTL20
H3F3C
FGD4
DENND5B
AMN1
KIAA1551
BICD1
YARS2
PKP2
32.0
32.5
33.0
Chr12 (Mb)
q
USP44
METAP2
AMDHD1
NR2C1
ELK3
HAL
CCDC38
TMCC3
FGD6
SNRPF
C12orf55
CCDC41
NDUFA12
VEZT
USP44
NTN4
LTA4H
CDK17
95.0
95.5
96.0
96.5
97.0
Chr12 (Mb)

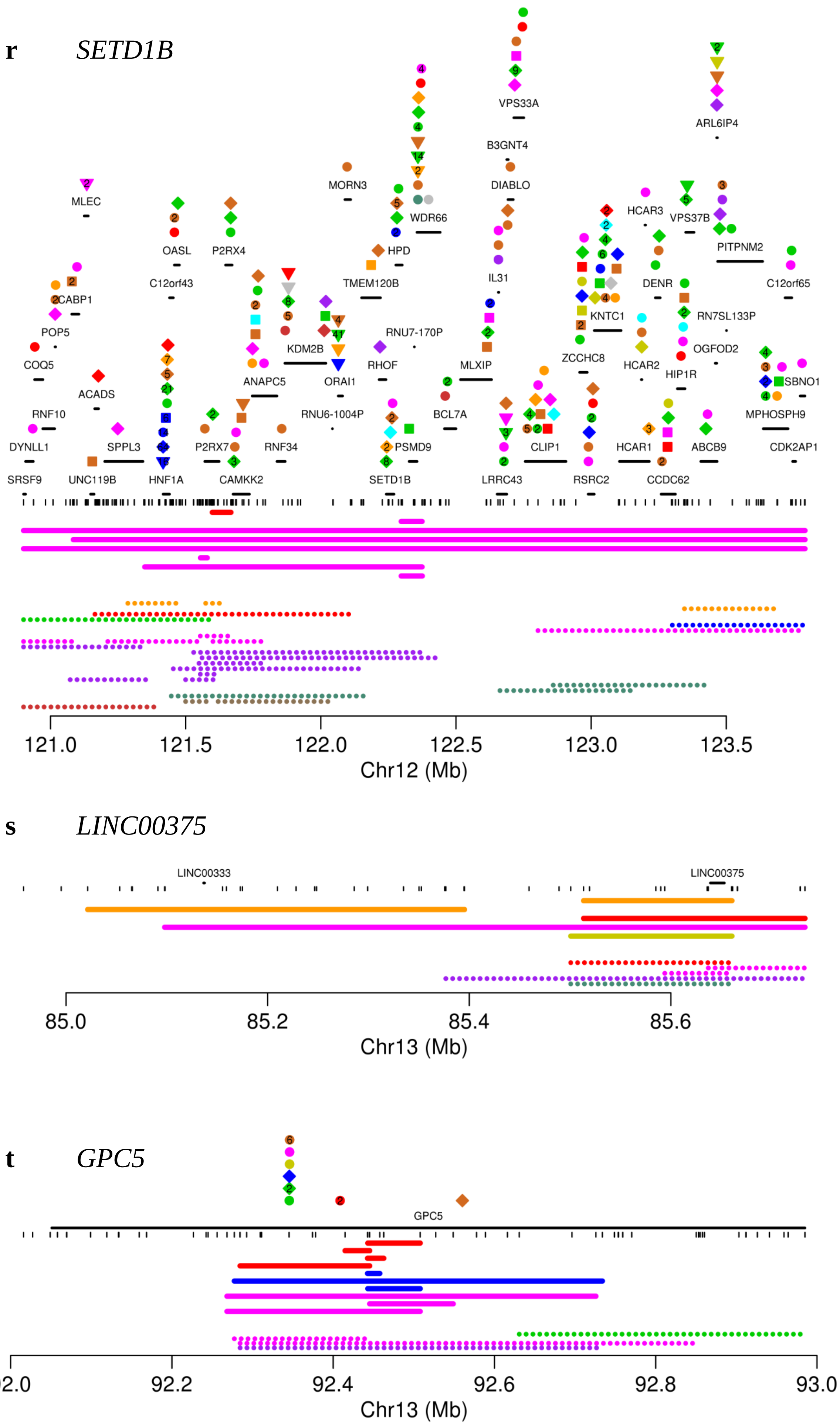
r
SETD1B
VPS33A
ARL6IP4
B3GNT4
MORN3
DIABLO
MLEC
HCAR3
VPS37B
OASL
P2RX4
WDR66
HPD
PITPNM2
C12orf43
TMEM120B
IL31
DENR
C12orf65
CABP1
KNTC1
RN7SL133P
POP5
RNU7-170P
KDM2B
OGFOD2
COQ5
RHOF
MLXIP
ZCCHC8
HCAR2
HIP1R
ANAPC5
ORAI1
SBNO1
ACADS
RNF10
RNU6-1004P
BCL7A
MPHOSPH9
DYNLL1
SPPL3
P2RX7
RNF34
PSMD9
CLIP1
HCAR1
ABCB9
CDK2AP1
SRSF9
UNC119B
HNF1A
CAMKK2
SETD1B
LRRC43
RSRC2
CCDC62
121.0
121.5
122.0
122.5
123.0
123.5
Chr12 (Mb)
s
LINC00375
LINC00333
LINC00375
85.0
85.2
85.4
85.6
Chr13 (Mb)
t
GPC5
GPC5
92.0
92.2
92.4
92.6
92.8
93.0
Chr13 (Mb)

u *SOX21*

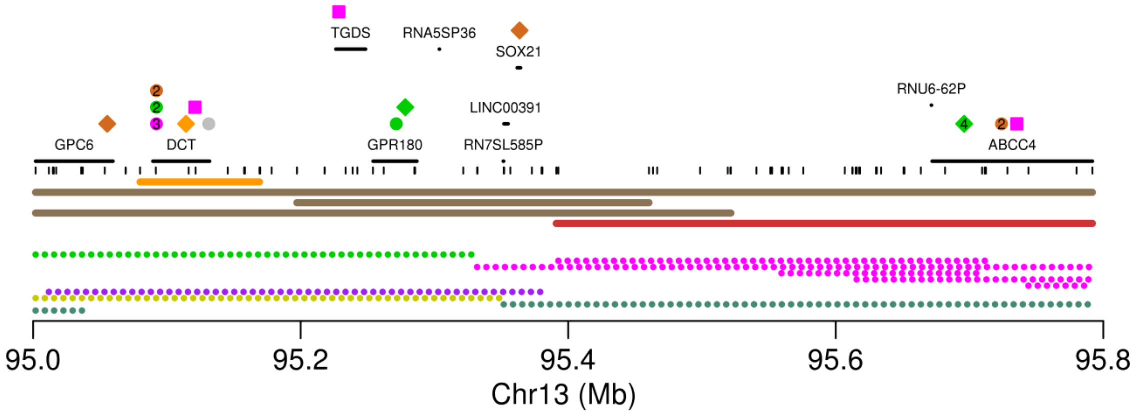

v *BAZ1A*

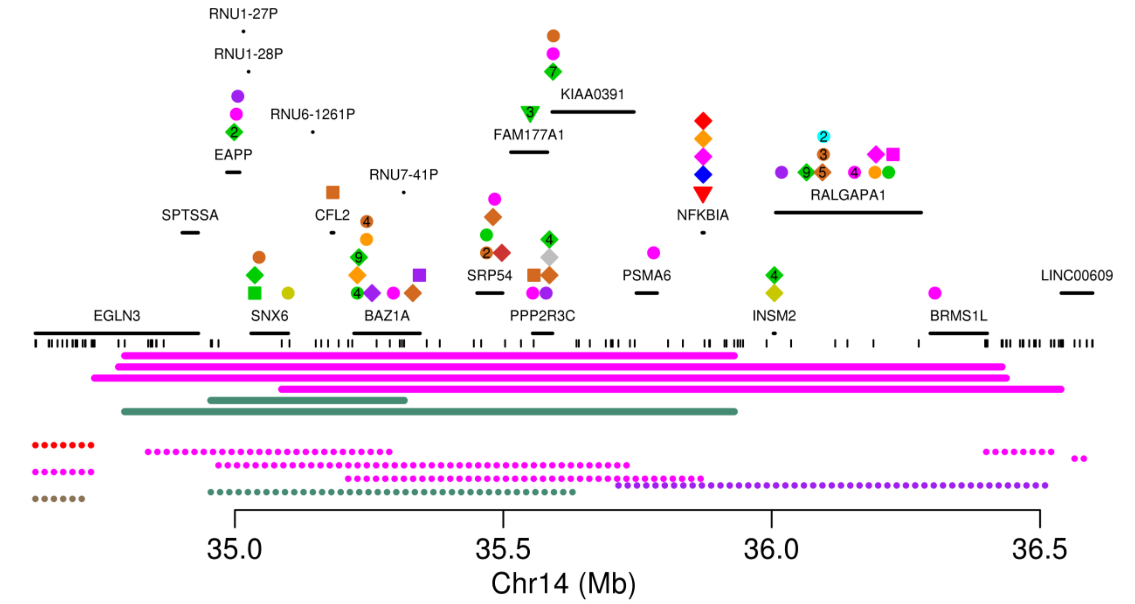

w *MIDEAS*

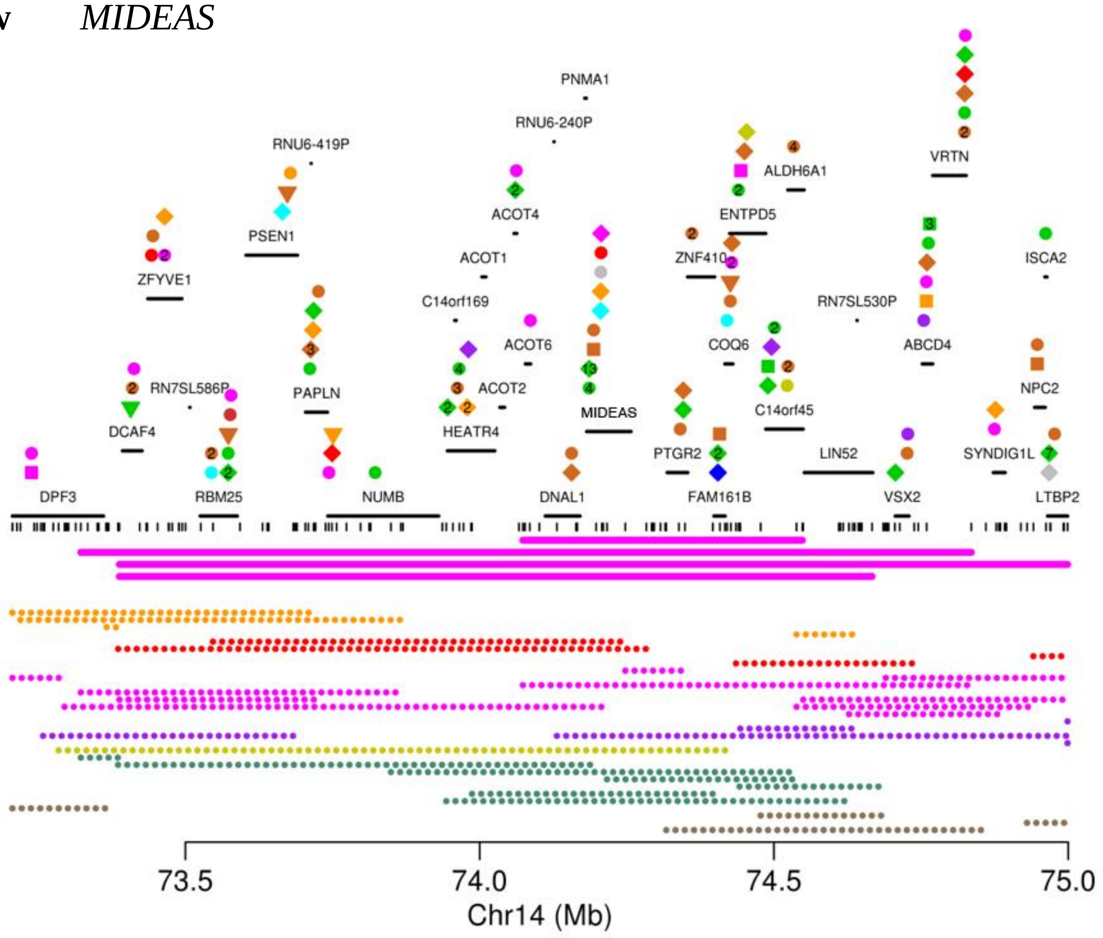

x *RFWD3*

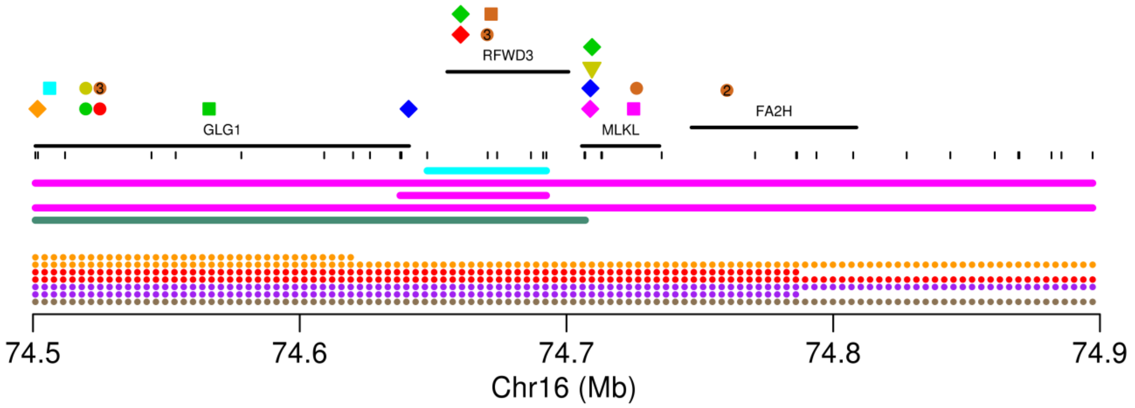

y *MAFTRR*

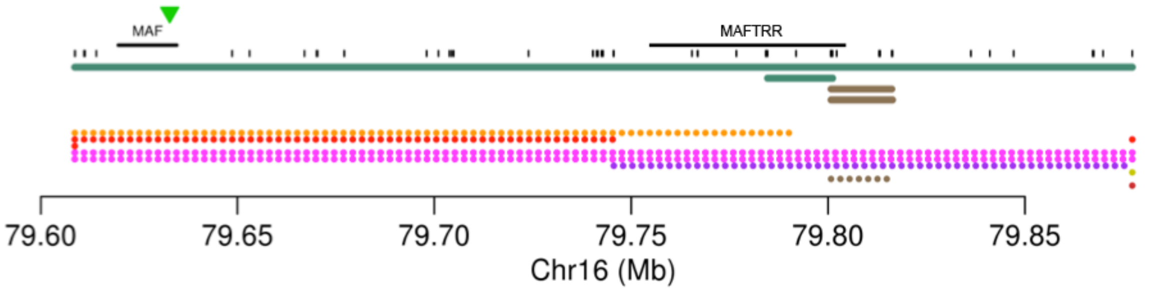

z *LINC00662*

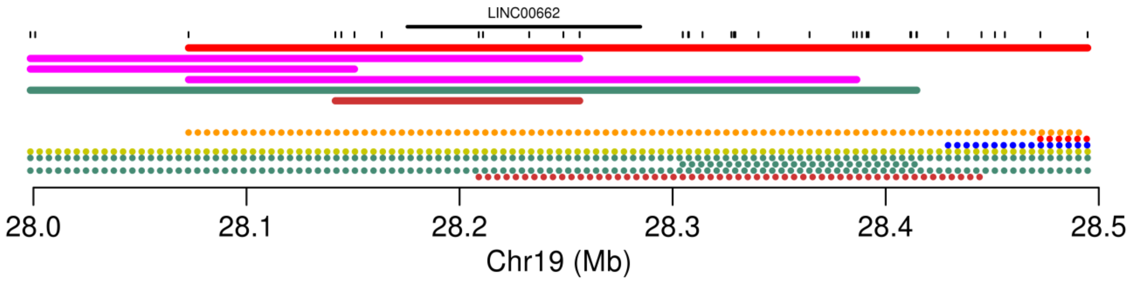

Supplementary Table 1: Overview of studies included in our compendium

Cancer type	Study	Number of samples
Breast cancer	Haverty *et al*., 2008[1]	46
	Kadota *et al*., 2009[2]	39
	Hawthorn *et al*., 2010[3]	19
	Beroukhim *et al*., 2010[4]	92
Ovarian cancer	Haverty *et al*., 2009[5]	29
	Beroukhim *et al*., 2010[4]	96
	Wertz *et al*., 2011[6]	16
Colorectal cancer	Firestein *et al*., 2008[7]	121
	Beroukhim *et al*., 2010[4]	8
Hepatocellular carcinoma	Chiang et al., 2008[8]	100
	Beroukhim *et al*., 2010[4]	7
Renal cancer	Beroukhim *et al*., 2009[9]	66
	Beroukhim *et al*., 2010[4]	10
Lung cancer	Weir *et al*., 2007[10]	167
	Bass *et al*., 2009[11]	46
	Beroukhim *et al*., 2010[4]	207
Tumours of the brain or nervous system	Northcott *et al*., 2009[12]	120
	Li *et al*., 2009[13]	37
	Chen *et al*., 2010[14]	26
	Beroukhim *et al*., 2010[4]	141

Oesophageal cancer	Bass *et al*., 2009[11]<br>Yang *et al*., 2010[15]	28<br>30
Sarcoma	Barretina *et al*., 2010[16]<br>Christensen *et al*., 2010[17]<br>Beroukhim *et al*., 2010[4]	206<br>23<br>19
Multiple myeloma	Avet-Loiseau *et al*., 2009[18]<br>Walker *et al*., 2010[19]	192<br>30
Leukaemia	Paulsson *et al*., 2008[20]<br>Tosello *et al*., 2009[21]<br>Bullinger *et al*., 2010[22]<br>Lilljebjörn *et al*., 2010[23]<br>Green *et al*., 2011[24]	45<br>9<br>67<br>23<br>10
Lymphoma	Kato *et al*., 2009[25]<br>Hartmann *et al*., 2010[26]<br>Scholtysik *et al*., 2010[27]<br>Green *et al*., 2011[24]	17<br>47<br>39<br>40
**Total unique primary cancer samples**		**2218**

Supplementary Table 2

Peaks of homozygous deletions over established tumour suppressors, T-cell receptor and immunoglobulin regions, known (named) fragile sites, predicted intra-chrosomsomal fragile sites, telomeric regions showing increased genomic instability, candidate tumour suppressors and regions showing a signature of positive selection for homozygous deletions but without a clear target gene. Each region's genomic position is shown, the number of homozygous deletions (HDs), a *p*-value (and multiple testing-corrected *q*-value) indicating the probability that the enrichment in homozygous deletions is due to increased genomic instability (rather than due to positive selection), the established or candidate target tumour suppressor gene or fragile site name and a reference to the supplementary figure showing that region.

Known tumour suppressors

Peak region	# of HDs	*p*-value (*q*-value)	Tumour suppressor	Supp. Fig.
chr1:51.58-53.53	4	4.16×10^{-2} (7.42×10^{-2})	*CDKN2C*	3a
chr3:10.18-10.20	6	0.229 (0.293)	*FANCD2/VHL*	3b
chr4:187.75-187.90	4	2.57×10^{-5} (2.82×10^{-4})	*FAT1*	3c
chr9:22.02-22.02	108	1.30×10^{-3} (4.38×10^{-3})	*CDKN2A (/CDKN2B)* [a]	3d
chr10:70.05-70.95	8	7.37×10^{-5} (6.09×10^{-4})	*TET1*	3e
chr10:89.74-89.83	16	6.05×10^{-9} (5.51×10^{-7})	*PTEN*	3f
chr11:101.95-102.05	6	4.87×10^{-4} (1.85×10^{-3})	*BIRC3 (/BIRC2)* [a]	3g
chr13:32.92-33.05	4	3.18×10^{-8} (1.45×10^{-6})	*BRCA2*	3h
chr13:49.04-49.09	23	1.52×10^{-7} (4.61×10^{-6})	*RB1*	3i
chr16:50.72-50.94	5	2.00×10^{-3} (6.11×10^{-3})	*CYLD*	3j
chr16:68.64-69.95	6	9.46×10^{-3} (2.39×10^{-2})	*CDH1* [b]	3k
chr17:7.58-7.58	4	2.08×10^{-2} (4.21×10^{-2})	*TP53*	3l
chr17:11.96-12.09	6	3.78×10^{-3} (1.07×10^{-2})	*MAP2K4* [b]	3m
chr17:29.55-29.83	4	2.02×10^{-3} (6.11×10^{-3})	*NF1*	3n
chr22:24.19-24.48	6	3.29×10^{-4} (1.50×10^{-3})	*SMARCB1*	3o

T-cell receptor and immunoglobulin loci

**Peak region**	**# of HDs**	***p*-value (*q*-value)**	**Locus**	**Supp. Fig.**
chr14:22.35-22.45	5	0.218 (0.288)	*TCRA*	4a
chr14:106.97-107.25	8	2.79×10^{-5} (2.82×10^{-4})	*IGH*	4b
chr22:22.91-23.14	18	2.53×10^{-2} (4.95×10^{-2})	*IGL*	4c

Known (named) fragile sites

**Peak region**	**# of HDs**	***p*-value (*q*-value)**	**Fragile site**	**Supp. Fig.**
chr1:214.97-214.97	17	0.445 (0.512)	FRA1H	5a
chr2:141.94-142.08	6	0.529 (0.587)	FRA2F	5b
chr3:60.41-60.45	7	0.823 (0.871)	FRA3B	5c
chr6:14.59-14.61	5	0.629 (0.69)	FRA6A	5d
chr6:33.06-33.07	6	0.233 (0.295)	FRA6H	5e
chr6:74.11-74.32	6	0.197 (0.268)	FRA6D	5f
chr6:109.59-109.59	6	0.153 (0.224)	FRA6F	5g
chr10:123.60-123.68	4	0.227 (0.293)	FRA10F	5h
chr12:114.60-114.63	7	0.852 (0.891)	FRA12C	5i
chr16:68.64-69.95	6	9.46×10^{-3} (2.39×10^{-2})	FRA16B [b]	5j
chr16:78.72-78.94	5	0.272 (0.335)	FRA16D	5k
chr17:11.96-12.09	6	3.78×10^{-3} (1.07×10^{-2})	FRA17A [b]	5l
chr19:31.87-31.89	4	0.407 (0.475)	FRA19A	5m
chrX:6.89-7.07	1	1 [c]	FRAXB	5n
chrX:32.81-33.16	1	1 [c]	FRAXC	5o

Predicted intrachromosomal fragile sites

**Peak region**	**# of HDs**	***p*-value (*q*-value)**		**Supp. Fig.**
chr2:61.83-61.96	6	4.85 x 10^{-2} (8.48 x 10^{-2})		6a
chr3:73.00-73.04	4	5.6 x 10^{-2} (9.52 x 10^{-2})		6b
chr3:116.69-116.83	4	0.197 (0.268)		6c
chr4:78.17-78.21	5	0.143 (0.22)		6d
chr6:18.17-18.21	4	0.213 (0.285)		6e
chr6:40.56-40.61	5	0.907 (0.928)		6f
chr8:3.32-3.39	6	6.45 x 10^{-2} (0.107)	[d]	6g
chr8:6.38-6.43	2	3.9 x 10^{-2} (7.10 x 10^{-2})	[d]	6h
chr8:98.84-98.85	8	0.458 (0.521)		6i
chr9:121.64-121.65	8	0.355 (0.425)		6j
chr9:133.39-133.62	4	0.166 (0.239)		6k
chr10:12.21-12.46	6	3.33 x 10^{-2} (6.32 x 10^{-2})		6l
chr13:28.25-28.26	5	9.53 x 10^{-2} (0.155)		6m
chr14:26.40-28.04	4	0.150 (0.224)		6n
chr14:96.22-96.22	4	0.758 (0.812)		6o
chr15:50.66-51.11	6	0.151 (0.224)		6p
chr16:6.74-6.76	7	0.918 (0.929)		6q
chr16:9.40-9.40	4	0.406 (0.475)		6r
chr16:10.07-10.08	5	0.964 (0.964)		6s
chr16:70.81-71.58	4	0.140 (0.219)		6t
chr19:9.06-9.07	4	0.520 (0.585)		6u
chr20:15.08-15.11	8	0.168 (0.239)		6v
chrX:3.22-4.12	3	1 [c]		6w
chrX:9.10-10.97	1	0.742 [c]		6x

Telomeric regions showing increased instability

**Peak region**	**# of HDs**	***p*-value (*q*-value)**		**Supp. Fig.**
chr2:0.92-0.94	5	0.900 (0.928)		7a
chr6:170.76-170.91	4	0.140 (0.219)		7b
chr7:158.91-159.13	5	0.248 (0.309)		7c
chr8:0.42-0.78	8	5.65×10^{-2} (9.52×10^{-2})	[d]	7d
chr9:0.76-0.88	5	0.680 (0.736)		7e
chr13:113.09-115.05	5	3.62×10^{-2} (6.72×10^{-2})		7f
chr17:80.94-81.01	6	0.331 (0.401)		7g
chr18:76.71-77.80	4	0.176 (0.247)		7h
chrX:0.10-1	4	0.167 [c]		7i

Regions showing a signature of positive selection for homozygous deletions but without a clear target gene

**Peak region**	**# of HDs**	***p*-value (*q*-value)**		**Supp. Fig.**
chr4:99.06-99.14	5	9.11×10^{-5} (6.91×10^{-4})	unknown	8a
chr4:182.34-182.70	4	1.68×10^{-3} (5.47×10^{-3})	intergenic	8b
chr8:34.29-34.30	9	2.28×10^{-4} (1.16×10^{-3})	intergenic	8c
chr13:20.34-20.45	4	3.9×10^{-4} (1.66×10^{-3})	unknown	8d
chr13:39.54-39.54	6	1.83×10^{-2} (3.97×10^{-2})	unknown	8e
chr17:0.01-0.05	4	2.08×10^{-3} (6.11×10^{-3})	unknown	8f

Candidate tumour suppressors

**Peak region**	**# of HDs**	***p*-value (*q*-value)**		**Sup. Fig.**
chr1:15.90-15.92	7	4.39×10^{-4} (1.74×10^{-3})	*CASP9*	9a
chr1:17.58-17.63	4	1.99×10^{-4} (1.16×10^{-3})	*ARHGEF10L*	9b
chr3:185.44-185.53	4	1.85×10^{-6} (3.37×10^{-5})	*IGF2BP2*	9c
chr4:39.08-39.15	12	8.87×10^{-4} (3.23×10^{-3})	*N4BP2*	9d
chr4:83.68-83.68	4	1.04×10^{-6} (2.36×10^{-5})	*HELQ/FAM175A*	9e
chr4:185.60-185.65	4	2.43×10^{-4} (1.16×10^{-3})	*CASP3*	9f
chr4:189.47-190.50	5	1.04×10^{-2} (2.56×10^{-2})	*LINC01060*	9g
chr5:58.41-58.41	4	4.00×10^{-4} (1.66×10^{-3})	*PDE4D* [e]	9h
chr5:68.40-68.69	6	9.32×10^{-4} (3.26×10^{-3})	*RAD17*	9i
chr8:1.77-1.94	8	4.68×10^{-6} (7.09×10^{-5})	*ARHGEF10* [d]	9j
chr8:29.97-29.98	4	1.63×10^{-2} (3.72×10^{-2})	*LEPROTL1*	9k
chr9:9.42-9.64	5	7.36×10^{-3} (1.97×10^{-2})	*PTPRD*	9l
chr10:76.72-76.81	5	3.59×10^{-5} (3.27×10^{-4})	*KAT6B*	9m
chr10:93.99-94.03	5	1.91×10^{-4} (1.16×10^{-3})	*CPEB3*	9n
chr10:131.42-131.49	5	1.74×10^{-4} (1.16×10^{-3})	*MGMT*	9o
chr12:32.15-32.24	5	1.29×10^{-2} (3.01×10^{-2})	*KIAA1551*	9p
chr12:95.88-96.27	4	2.01×10^{-2} (4.16×10^{-2})	*USP44*	9q
chr12:122.30-122.37	6	2.21×10^{-5} (2.82×10^{-4})	*SETD1B*	9r
chr13:85.51-85.66	4	1.81×10^{-2} (3.97×10^{-2})	*LINC00375*	9s
chr13:92.45-92.45	10	2.16×10^{-4} (1.16×10^{-3})	*GPC5*	9t
chr13:95.39-95.46	4	2.35×10^{-4} (1.16×10^{-3})	*SOX21*	9u
chr14:35.09-35.32	6	1.94×10^{-2} (4.11×10^{-2})	*BAZ1A*	9v
chr14:74.07-74.55	4	2.55×10^{-2} (4.95×10^{-2})	*MIDEAS*	9w
chr16:74.65-74.69	5	4.65×10^{-3} (1.28×10^{-2})	*RFWD3*	9x
chr16:79.80-79.80	4	7.58×10^{-3} (1.97×10^{-2})	*MAFTRR*	9y
chr19:28.14-28.15	6	1.18×10^{-2} (2.83×10^{-2})	*LINC00662*	9z

[a]*CDKN2B* and *BIRC2* are candidate tumour suppressor genes with a high level of evidence. They are always lost together with *CDKN2A* and *BIRC3*, respectively, and likely contribute to positive selection of the homozygous deletions.

[b]Known fragile sites that contain a known tumour suppressor are shown twice in the table.

[c]*p*-values for regions on the X chromosome derive from testing tumour-type specificity in females only.

[d]The region 1-8Mb on chromosome 8 contains 4 peaks. In order to separate the effects of the different regions, 5 large homozygous deletions (> 5 Mb) overlapping all four peaks are not included in the counts input into the statistical model to separate tumour suppressors from fragile sites.

[e]*PDE4D* shows intragenic homozygous deletions, suggesting these deletions may be gain-of-function rather than loss-of-function mutations.

Supplementary References

1 Haverty, P. M. *et al.* High-resolution genomic and expression analyses of copy number alterations in breast tumors. *Genes Chromosomes Cancer* **47**, 530-542 (2008).

2 Kadota, M. *et al.* Identification of novel gene amplifications in breast cancer and coexistence of gene amplification with an activating mutation of PIK3CA. *Cancer Res* **69**, 7357-7365 (2009).

3 Hawthorn, L., Luce, J., Stein, L. & Rothschild, J. Integration of transcript expression, copy number and LOH analysis of infiltrating ductal carcinoma of the breast. *BMC Cancer* **10**, 460 (2010).

4 Beroukhim, R. *et al.* The landscape of somatic copy-number alteration across human cancers. *Nature* **463**, 899-905 (2010).

5 Haverty, P. M., Hon, L. S., Kaminker, J. S., Chant, J. & Zhang, Z. High-resolution analysis of copy number alterations and associated expression changes in ovarian tumors. *BMC Med Genomics* **2**, 21 (2009).

6 Wertz, I. E. *et al.* Sensitivity to antitubulin chemotherapeutics is regulated by MCL1 and FBW7. *Nature* **471**, 110-114 (2011).

7 Firestein, R. *et al.* CDK8 is a colorectal cancer oncogene that regulates beta-catenin activity. *Nature* **455**, 547-551 (2008).

8 Chiang, D. Y. *et al.* Focal gains of VEGFA and molecular classification of hepatocellular carcinoma. *Cancer Res* **68**, 6779-6788 (2008).

9 Beroukhim, R. *et al.* Patterns of gene expression and copy-number alterations in von-hippel lindau disease-associated and sporadic clear cell carcinoma of the kidney. *Cancer Res* **69**, 4674-4681 (2009).

10 Weir, B. A. *et al.* Characterizing the cancer genome in lung adenocarcinoma. *Nature* **450**, 893-898 (2007).

11 Bass, A. J. *et al.* SOX2 is an amplified lineage-survival oncogene in lung and esophageal squamous cell carcinomas. *Nat Genet* **41**, 1238-1242 (2009).

12 Northcott, P. A. *et al.* Multiple recurrent genetic events converge on control of histone lysine methylation in medulloblastoma. *Nat Genet* **41**, 465-472 (2009).

13 Li, M. *et al.* Frequent amplification of a chr19q13.41 microRNA polycistron in aggressive primitive neuroectodermal brain tumors. *Cancer Cell* **16**, 533-546 (2009).

14 Chen, R. *et al.* A hierarchy of self-renewing tumor-initiating cell types in glioblastoma. *Cancer Cell* **17**, 362-375 (2010).

15 Yang, H. H. *et al.* Influence of genetic background and tissue types on global DNA methylation patterns. *PLoS One* **5**, e9355 (2010).

16 Barretina, J. *et al.* Subtype-specific genomic alterations define new targets for soft-tissue sarcoma therapy. *Nat Genet* **42**, 715-721 (2010).

17 Christensen, B. C. *et al.* Integrated profiling reveals a global correlation between epigenetic and genetic alterations in mesothelioma. *Cancer Res* **70**, 5686-5694 (2010).

18 Avet-Loiseau, H. *et al.* Prognostic significance of copy-number alterations in multiple myeloma. *J Clin Oncol* **27**, 4585-4590 (2009).

19 Walker, B. A. *et al.* A compendium of myeloma-associated chromosomal copy number abnormalities and their prognostic value. *Blood* **116**, e56-65 (2010).

20 Paulsson, K. *et al.* Microdeletions are a general feature of adult and adolescent acute lymphoblastic leukemia: Unexpected similarities with pediatric disease. *Proc Natl Acad Sci U S A* **105**, 6708-6713 (2008).

21 Tosello, V. *et al.* WT1 mutations in T-ALL. *Blood* **114**, 1038-1045 (2009).

22 Bullinger, L. *et al.* Identification of acquired copy number alterations and uniparental disomies in cytogenetically normal acute myeloid leukemia using high-resolution single-nucleotide polymorphism analysis. *Leukemia* **24**, 438-449 (2010).

23 Lilljebjorn, H. *et al.* The correlation pattern of acquired copy number changes in 164 ETV6/RUNX1-positive childhood acute lymphoblastic leukemias. *Hum Mol Genet* **19**, 3150-3158 (2010).

24 Green, M. R. *et al.* Integrative genomic profiling reveals conserved genetic mechanisms for tumorigenesis in common entities of non-Hodgkin's lymphoma. *Genes Chromosomes Cancer* **50**, 313-326 (2011).

25 Kato, M. *et al.* Frequent inactivation of A20 in B-cell lymphomas. *Nature* **459**, 712-716 (2009).

26 Hartmann, S. *et al.* High resolution SNP array genomic profiling of peripheral T cell lymphomas, not otherwise specified, identifies a subgroup with chromosomal aberrations affecting the REL locus. *Br J Haematol* **148**, 402-412 (2010).

27 Scholtysik, R. *et al.* Detection of genomic aberrations in molecularly defined Burkitt's lymphoma by array-based, high resolution, single nucleotide polymorphism analysis. *Haematologica* **95**, 2047-2055 (2010).
