# Supplementary material for: Pan-cancer analysis of homozygous deletions in primary tumours uncovers rare tumour suppressors
Source: Nat Commun. 2017 Oct 31;8:1221. doi: 10.1038/s41467-017-01355-0 (PMC5663922; doi:10.1038/s41467-017-01355-0)
Supplement: Supplementary file 2 — Description of Additional Supplementary Files [file 41467_2017_1355_MOESM2_ESM.pdf]

Description of Additional Supplementary Files

File Name: Supplementary Data 1
Description: List of samples included in this study

File Name: Supplementary Data 2
Description: List of homozygous deletions identified in this study

File Name: Supplementary Data 3
Description: Results of permutation analyses to identify regions with excess homozygous deletions
